# Stir-Frying Reshapes the Chemical Profile and Metabolic Effects of *Raphani Semen* in Diabetic Mice: Integrated Analysis of Hepatic Metabolism and Gut Microbiota

**DOI:** 10.3390/foods15203587

**Published:** 2026-10-09

**Authors:** Chenxi Bai, Fazhi Su, Minghao Sun, Meng Liu, Yanping Sun, Qiuhong Wang, Haixue Kuang

**Affiliations:** 1 Key Laboratory of Basis and Application Research of Beiyao, Ministry of Education, Heilongjiang University of Chinese Medicine, Harbin 150040, China; 2 School of Chinese Materia Medica and Health, Bozhou University, Bozhou 236800, China; 3 School of Traditional Chinese Medicine, Guangdong Pharmaceutical University, Guangzhou 510006, China

**Keywords:** *Raphani Semen*, stir-frying, functional food, glucose homeostasis, insulin resistance, thermal processing, multi-omics

## Abstract

Stir-frying can alter the chemical composition and biological functionality of *Raphani Semen* (RS), but whether this processing improves its glucose-regulating properties remains unclear. In this study, a representative laboratory-scale stir-frying condition (160 °C for 3 min) was selected through exploratory chromatographic, macroscopic, and organoleptic assessment. Raw RS and stir-fried *Raphani Semen* (SF-RS) were subsequently compared using chemical and serum-exposure profiling, metabolic phenotyping, liver histopathology, hepatic proteomics and metabolomics, gut microbiota analysis, and targeted molecular validation in a diet/streptozotocin-induced mouse model of type 2 diabetes. Stir-frying altered relative LC-MS chemical response patterns and serum-detectable feature profiles, with selected glucosinolate-related signals showing the most consistent processing-associated differences. Functionally, SF-RS produced significantly greater reductions in terminal fasting glucose, insulin, and HOMA-IR than raw RS at all three matched doses (*p* < 0.05), while its advantage in OGTT AUC was particularly evident at the low dose. SF-RS also enhanced hepatic PAS-positive glycogen restoration across the tested dose range and improved lipid accumulation and collagen deposition at selected doses. Multi-omics analyses indicated processing-dependent regulation of PI3K–Akt/FoxO, PPAR, lipid, and cofactor metabolic pathways, together with distinct remodeling of the gut microbial community. Targeted validation further showed stronger regulation of PI3K-Akt signaling, Forkhead Box O1 (FoxO1) nuclear accumulation, and PPAR-α/CPT1A/ACOX1-associated fatty-acid metabolism after stir-frying. These findings indicate that thermal processing strengthens the glucose- and insulin-related functionality of *Raphani Semen* and enhances its potential value as a functional food ingredient for metabolic health.

## 1. Introduction

Type 2 diabetes mellitus (T2DM) is a major global metabolic disease. Its growing prevalence reflects the combined effects of genetic, demographic, environmental, and lifestyle factors, including population aging, physical inactivity, unhealthy diets, and the increasing prevalence of overweight and obesity [1,2]. T2DM is not limited to persistent hyperglycemia. It is often accompanied by dyslipidemia, impaired hepatic metabolism, chronic low-grade inflammation, and cardiovascular and renal complications, which can gradually damage multiple organs [1,2]. Healthy dietary patterns and regular physical activity remain central to T2DM care, together with appropriate pharmacotherapy. This need has encouraged research on food-derived bioactive materials that may regulate glucose and lipid metabolism through several complementary pathways.

Plants traditionally used as both foods and medicines are important sources of compounds with potential metabolic benefits. A recent systematic review summarized experimental evidence showing that polysaccharides from these plants may affect glucose and lipid metabolism, gut microbial composition, inflammatory responses, and oxidative stress in T2DM models [3]. Some food-derived materials have also progressed to clinical evaluation. *Momordica charantia* L. (bitter melon), for example, has been widely studied for its glucose-regulating effects, and a systematic review and meta-analysis reported changes in glycemic outcomes among individuals with T2DM [4]. Mulberry-derived alkaloids have been evaluated in a multicenter randomized clinical trial [5], and the available clinical evidence was subsequently assessed in a systematic review and meta-analysis [6]. Together, these studies suggest that food-compatible bioactive materials may have translational value. They also show that biological effects should be linked to defined chemical constituents, processing methods, systemic exposure, and plausible molecular mechanisms.

Reliable experimental models are needed to compare metabolic effects and investigate mechanisms before human translation. Genetic, diet-induced, and chemically induced models are commonly used in diabetes research, but each reproduces only part of the human disease and has specific limitations [7]. Feeding animals a high-fat or high-sugar/high-fat diet (HSHFD) in combination with low-dose streptozotocin (STZ) produces a phenotype in which diet-induced insulin resistance is accompanied by partial pancreatic β-cell dysfunction [8]. Mice with a c57BL/6 genetic background are particularly susceptible to metabolic abnormalities caused by obesogenic diets, as shown in early studies of diet-induced glucose intolerance and diabetes [9]. More recently, high-fat-diet feeding combined with low-dose STZ was shown to induce fasting hyperglycemia, hyperinsulinemia, and insulin resistance in male c57BL/6J mice. That study also showed that sex and experimental conditions can strongly affect the resulting phenotype [10]. Diet/STZ-based models are therefore useful for evaluating the glucose-regulating effects of food- and plant-derived materials, although they do not fully reproduce the complexity of human T2DM [7,10].

*Raphanus sativus* L. is a widely consumed cruciferous vegetable with a diverse nutritional and phytochemical composition [11]. Its dried mature seeds, known as *Raphani Semen* or Lai Fu Zi, are used at the interface between food and traditional medicine. In China, *Raphani Semen* was included in the Ministry of Health list of substances traditionally used as both foods and medicines [12]. Chemical studies have identified glucosinolates and their degradation products, phenolic compounds, flavonoids, alkaloids, lipids, and several other classes of constituents in *Raphani Semen*. Experimental studies have also reported effects related to gastrointestinal function, hepatic metabolism, inflammation, and metabolic disorders [13]. Recent findings provide more direct evidence of the metabolic activity of radish seeds. A glucosinolate-rich radish-seed preparation attenuated high-fat-diet-induced obesity and lipid metabolic abnormalities and was associated with changes in gut microbial composition and fecal metabolites [14]. In another study, an aqueous radish-seed extract and its characteristic isothiocyanate, sulforaphene, improved glucose homeostasis and insulin sensitivity in high-fat-diet-fed mice. These treatments also promoted adipocyte mitochondrial remodeling and increased markers related to thermogenesis [15]. These findings support the view that radish seeds are biologically active cruciferous materials with potential value in metabolic health research.

Food processing may also influence the biological activity of *Raphani Semen*, but this aspect has received less attention. Stir-frying is a traditional thermal treatment of *Raphani Semen*, and raw and stir-fried products have long been considered to have different functional properties. Modern chemical studies support this distinction. Comparative HPLC and UPLC–Q-TOF-MS analyses have shown that thermal processing changes several representative constituents of *Raphani Semen* [16]. Later qualitative and quantitative studies further described changes in component levels and possible transformation pathways during stir-frying [17]. These chemical changes are relevant from a food-science perspective because heating may alter compound stability, release from the food matrix, extractability, and conversion into new products. Such changes may, in turn, affect which constituents are absorbed after oral administration. However, previous studies of processed *Raphani Semen* have mainly described its chemical transformation [16,17] or examined mechanisms related to traditional digestive indications, such as functional dyspepsia [18]. It remains unclear whether processing-related chemical changes alter the constituents detectable in circulation, whether they modify metabolic efficacy, and which molecular pathways may account for any difference between raw and stir-fried preparations.

Addressing these questions requires methods that examine several biological levels together. T2DM involves coordinated changes in metabolic pathways, signaling networks, inflammatory processes, and host–microbial interactions. Proteomic, metabolomic, and microbiome-based methods have therefore become useful for characterizing these changes at the systems level [19]. For complex botanical preparations, integrated multi-omics analysis can also help connect changes in chemical exposure with tissue-level pathways, microbial features, and physiological outcomes [20].

We therefore tested the hypothesis that stir-frying improves the metabolic activity of *Raphani Semen* and investigated the mechanisms that might explain this effect. A representative stir-frying condition was first selected, after which the phytochemical profiles of raw and stir-fried *Raphani Semen* were compared. Constituents detected in serum after administration were also analyzed to determine whether processing changed both the administered material and the compounds available in circulation. The effects of the two preparations on glucose homeostasis, insulin resistance, lipid metabolism, and hepatic pathology were then compared in a diet/STZ-induced mouse model of T2DM. Hepatic proteomics and metabolomics were integrated with 16S rRNA gene sequencing of the gut microbiota to identify processing-responsive pathways and host–microbial associations related to the activity of stir-fried *Raphani Semen*. Finally, selected metabolic and insulin-signaling pathways were experimentally validated, including the subcellular regulation of FoxO1.

## 2. Materials and Methods

### 2.1. Chemical and Reagents

Raw *Raphani Semen* (RS) seed samples, consisting of the dried mature seeds of *Raphanus sativus* L. (*Brassicaceae*), were purchased from Jilin Province Yushu Trading Co., Ltd. (Yushu, China, batch number 2023101801). It has been authenticated by Professor Su Lianjie from the Department of Traditional Chinese Medicine Resources of Heilongjiang University of Chinese Medicine as the dried mature seeds of *Raphanus sativus* L., a cruciferous plant. The surface is reddish-brown, the seed coat is thin and brittle, and it is oily. This conforms to the provisions of the 2020 edition of the Chinese Pharmacopoeia. The sample specimens (voucher number 20240921004) are stored in the Key Laboratory of Basic and Applied Research of *Beiyao*, Ministry of Education, Heilongjiang University of Chinese Medicine. Foreign matter, visibly damaged seeds, and other extraneous materials were manually removed. All samples used for preliminary processing-condition screening and subsequent experiments originated from the same batch of raw material. Before processing, the seeds were thoroughly mixed and stored in sealed containers under dry conditions at room temperature.

The SPF-grade high-sugar and high-fat diet, consisting of 66.5% standard chow, 10% lard, 20% sucrose, 2.5% cholesterol, and 1% sodium cholate, was provided by Xiao Shu You Tai (Beijing, China) Biotechnology Co., Ltd. (SCXK-Jing-2023-0010, Beijing, China). Streptozocin (STZ, BS180-100 mg, purity ≥ 98%) was purchased from Lan Jie Ke Technology Co., Ltd. (Jinan, China). The blood glucose test strips and blood glucose meters were both produced by the Roche Group (Basel, Switzerland).

For all the analyses based on liquid chromatography-mass spectrometry (LC-MS), whether they are proteomics, metabolomics or component analysis, the specific information of the reagents is listed as follows: formic acid (A117-50), trifluoroacetic acid (TFA, No. 85183), Bond-Breaker^TM^ TCEP solution (TCEP, No. 77720), methanol (A452-4) and acetonitrile (A998-4) were supplied by Thermo Fisher Scientific (Waltham, MA, USA). Iodoacetemide (IAM, I6125-5G, purity ≥ 99%) and triethylammonium bicarbonate buffer (TEAB, T7 408–500 mL, LC-MS grade) were provided by Sigma-Aldrich Chemical Company (St. Louis, MO, USA).

### 2.2. Screening and Selection of Stir-Frying Conditions for Raphani Semen

A preliminary screening experiment was conducted to select a representative laboratory-scale stir-frying condition for preparing stir-fried *Raphani Semen* (SF-RS) for subsequent biological experiments. This screening was not designed as a formal process-optimization or process-validation study. Stir-frying without excipients was performed with reference to the clean stir-frying procedure described in General Rule 0213 of the Pharmacopoeia of the People’s Republic of China (2020 edition) [21].

Processing was conducted using a CZ30 type superconducting electric heating stir-frying machine (Beijing Huo’s Machinery Manufacturing Co., Beijing, China). Two processing factors were examined, comprising three nominal temperatures of 140, 160, and 180 °C and five processing durations of 1, 3, 5, 7, and 9 min. The frequency of drum rotation was set at 30 Hz. The complete combination of these factors generated 15 processed samples. The samples were designated according to processing temperature and duration, for example RS-140-1, RS-160-5 and RS-180-9. A single processing run was performed for each temperature-time combination during this exploratory screening.

For each processing run, 250 g of cleaned RS was accurately weighed. The stir-frying apparatus was preheated to the designated temperature and maintained at the target setting for 3 min before sample loading. The temperatures reported in this screening refer to the temperature settings displayed by the apparatus. The batch load and agitation conditions were maintained consistently across all processing runs at 250 g and 30 Hz, respectively. Processing time was counted after the displayed apparatus temperature returned to the designated setting following sample loading.

At the designated processing time, the sample was immediately removed from the apparatus and spread as a single thin layer on a clean stainless-steel tray. The sample was allowed to cool to room temperature under ambient laboratory conditions to minimize continued thermal transformation after discharge. The processing apparatus was cleaned and allowed to return to the designated operating condition before the subsequent run. An unheated portion of the same raw-material batch was retained as the raw reference.

### 2.3. Qualitative Assessment of Appearance and Aroma

Following cooling, the 15 stir-fried samples were subjected to a standardized macroscopic and organoleptic assessment to support selection of a representative processing condition. Equal amounts of each sample were placed in identical white porcelain dishes, randomly coded, and evaluated independently by three assessors familiar with the processing characteristics of *Raphani Semen*. Evaluations were performed under standardized D65 illumination against a white background. Surface color was recorded descriptively, while color uniformity, apparent swelling, seed-coat integrity, crispness, roasted aroma, and burnt odor were rated using four-point ordinal scales from 0 to 3 according to predefined criteria. The results are reported as the median and interquartile range of the three individual ratings. These assessments were used only as screening criteria for processing-condition selection and were not intended as formal sensory or consumer-acceptance testing.

### 2.4. HPLC Analysis

#### 2.4.1. Exploratory Chromatographic Comparison

The raw sample and the 15 stir-fried samples were subjected to chromatographic analysis. The samples were analyzed using a high-performance liquid chromatograph (HPLC, Arc HPLC, Waters, Milford, MA, USA), with absorbance measured at 225 and 326 nm [22]. Then, 5 g of samples were ground into powder and placed in a 50 mL centrifuge tube. Then, 40 mL of 70% methanol-water solution was added, and ultrasonic extraction was performed twice, each time for 30 min. The two extraction solutions were combined and concentrated to 1 g/mL under low pressure, then they were stored in a 4 °C refrigerator for further analysis. Subsequently, 1 mL of each sample was taken and placed in a 15 mL centrifuge tube. Next, 10 mL of pure methanol was added, and the mixture was vortexed for 1 min. Then, the tube was centrifuged at 4 °C and 5000 rpm for 10 min. The supernatant was filtered through a 0.22 μm membrane for further HPLC injection.

The analysis was performed on an Arc HPLC system equipped with a Waters Symmetry C18 column (5 μm, 4.6 × 250 mm, No. WAT054275) maintained at 35 °C. The mobile phase was composed of A (water) and B (acetonitrile), with gradient elution program as follows: 0–9 min, 95–87% A; 9–33 min, 87–83.4% A; 33–40 min, 83.4–77% A; 40–50 min, 77–73% A; 50–56 min, 73–60% A; 56–56.01 min, 60–95% A; 56.01–60 min, 95% A. The flow rate was set at 0.8 mL/min, and the injection was 10 μL.

#### 2.4.2. Selection of the Representative Processing Condition

The processing condition used to prepare SF-RS for subsequent experiments was selected by integrating the macroscopic and organoleptic assessment with the exploratory HPLC profiles. The predefined selection criteria were: (i) moderate and relatively uniform surface darkening without visible charring; (ii) slight swelling and increased crispness; (iii) development of a mild roasted aroma without a pronounced burnt odor; and (iv) retention of the principal chromatographic signals observed in raw RS without extensive loss of chromatographic complexity. The chromatographic comparison was used as supportive evidence rather than as a quantitative optimization criterion. Accordingly, the selected condition was regarded as a representative processing condition within the tested temperature–time range, rather than an experimentally optimized processing parameter.

To assess the reproducibility of the stir-frying procedure, three independent SF-RS preparations were performed using the same representative processing conditions. Each preparation was conducted independently rather than as a technical replicate. A more comprehensive evaluation of processing reproducibility was conducted separately and will be reported in detail elsewhere; summary reproducibility data relevant to the present study are provided in Supplementary Materials.

### 2.5. Preparation of Aqueous Extract

For decoction preparation, accurately weighed (500 g) RS or SF-RS samples were separately mixed with distilled water at a solid-to-liquid ratio of 1:10 (*v*/*v*) and soaked for 1 h at room temperature. The soaked material was subsequently decocted under gentle boiling for 2 h. The extract was filtered, and the remaining residue was re-extracted once with fresh distilled water under the same conditions for another 2 h. The two aqueous extracts from each material were combined, respectively, filtered through a Buchner funnel, and concentrated to obtain a final concentration of 1 g crude material equivalent/mL. The prepared decoctions were designated as raw *Raphani Semen* aqueous decoction and stir-fried *Raphani Semen* aqueous decoction, respectively, and were stored at 4 °C prior to use.

### 2.6. UPLC-Q-Exactive-MS/MS-Based Chemical Profiling of Raw and Stir-Fried Raphani Semen and Analysis of Absorbed Constituents in Serum

The blood-absorbed constituents of RS and SF-RS were characterized using an ultra-performance liquid chromatography–high-resolution tandem mass spectrometry strategy (UPLC-HRMS). Briefly, RS and SF-RS samples were separately extracted according to the preparation procedure (Section 2.5) used for administration, and the resulting extracts were filtered through a 0.22 μm membrane before injection. Experimental animals (SPF-grade SD rats, 8 weeks old, 200–240 g) were randomly assigned to blank, RS-treated, and SF-RS-treated groups. After an appropriate fasting period with free access to water, animals in the treatment groups were orally administered the corresponding extract at a dosage of 1.4 g/kg for 14 consecutive days, whereas the blank group received the same volume of vehicle. Blood samples were collected 2 h after administration and centrifuged at 4 °C (4000 rpm for 10 min) to obtain serum samples.

For sample pretreatment, serum samples were mixed with ice-cold extracting solvent (acetonitrile:methanol = 1:1, *v*/*v*) containing internal standard (L-2-Chlorophenylalanine, 0.02 mg/mL). The mixtures were vortexed thoroughly (30 s), ultrasonically extracted (4 °C, 40 kHz, 30 min), and centrifuged at high speed at 4 °C (13,000 g, 15 min). The supernatants were collected and evaporated to dryness under a gentle nitrogen stream or by vacuum concentration. The residues were reconstituted with specific resolution (acetonitrile: water = 1:1, *v*/*v*), ultrasonically extracted (4 °C, 40 kHz, 30 min), centrifuged again (13,000× *g*, 15 min, 4 °C), and the final supernatants were transferred to autosampler vials for UPLC–MS/MS analysis. Quality-control samples were prepared by pooling equal aliquots from all processed biological samples and were injected periodically to monitor analytical stability. The detailed parameters for UPLC-HRMS were listed in Table S1.

Serum-associated exogenous constituents were screened by comparison of serum samples collected after RS or SF-RS administration with blank serum. Features showing treatment-associated enrichment relative to the corresponding blank-serum background were retained for subsequent structural annotation. Prototype constituents were defined as serum-detectable compounds that could be directly matched to constituents detected in the corresponding administered RS or SF-RS extract. Assignment of a compound as a putative prototype required concordance in compound annotation, accurate precursor *m*/*z*, formula, ionization mode/adduct, retention time, and characteristic MS/MS fragment ions between the serum and corresponding extract. Structural annotations were further evaluated against the HMDB and the commercial spectral database provided by Shanghai Majorbio Bio-Pharm Technology Co., Ltd. (Shanghai, China).

### 2.7. Animal Experiment and Sample Collection

**Figure 1:**
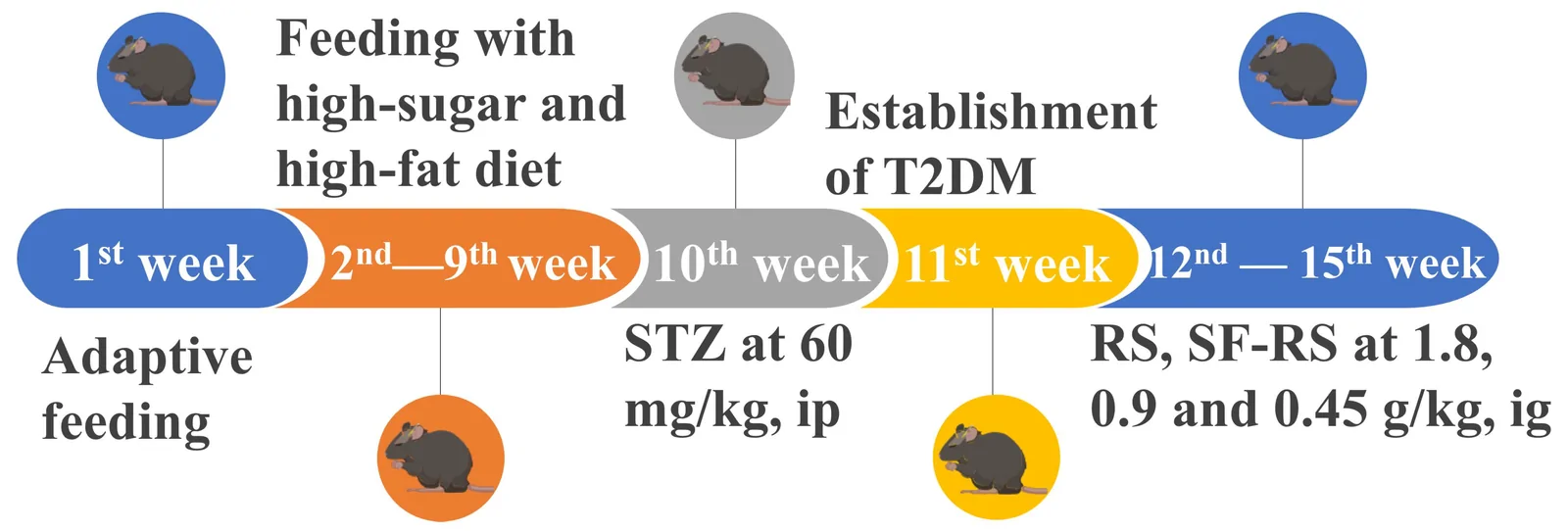
Experimental design and treatment schedule for evaluating raw and stir-fried *Raphani Semen* in a high-sugar/high-fat diet- and STZ-induced mouse model of T2DM.

The classical rodent model was used to evaluate the glucose-lowering efficacy of RS and SF-RS, as well as to explore their potential mechanisms of action, namely a diet/streptozotocin-induced type 2 diabetic mouse model. The overall experimental workflow is shown in Figure 1.

Seventy-two specific pathogen-free (SPF) male c57BL/6 mice, aged 8 weeks and weighing 18–22 g, were purchased from Liaoning Changsheng Biotechnology Co., Ltd. (Benxi, China) [SCXK (LIAO) 2025-0001]. All animals were maintained in the SPF animal facility of Heilongjiang University of Chinese Medicine under controlled environmental conditions: temperature, 23 ± 2 °C, relative humidity, 40–60%, and a 12 h light/12 h dark cycle. Animals were allowed to acclimatize for 1 week before the start of the experiments. All procedures were conducted in accordance with the NIH Guide for the Care and Use of Laboratory Animals and were approved by the Institutional Animal Ethics Committee of Heilongjiang University of Chinese Medicine (approval No. 2024062001). The choice of chemically induced model is consistent with recent recommendations emphasizing that this model can comprehensively recapitulate the pathogenesis of human type 2 diabetes and its complications [7].

After a period of seven-day adaptive feeding, all the experimental animals were initially divided into two groups: the blank control group (8 mice) and the T2DM model group (64 mice). Mice in the control group were fed a standard chow diet throughout the experiment, whereas mice in the remaining group were fed a high-sugar/high-fat diet for 8 weeks to induce obesity-associated metabolic stress and insulin resistance. After dietary induction, mice received repetitive intraperitoneal injections (once a day, 7 days) of STZ freshly dissolved in 0.1 mol/L citrate buffer at pH 4.5 at a dose of 60 mg/kg. Three days after STZ injection, fasting blood glucose was measured from the tail vein, and this was recorded as the initial blood glucose measurement. Similarly, three days later, the fasting blood glucose levels of the mice were measured again, and this was recorded as the second set of blood glucose readings. It was noted that both blood glucose tests were conducted during the day, with the same testing time and sequence. During both blood glucose monitoring sessions, the subjects were required to fast but not restrict water intake. The fasting duration for both sessions was 5 h. Mice were considered successfully diabetic when fasting blood glucose exceeded 11.1 mmol/L in two consecutive measurements. In the experiment, mice with blood glucose levels continuously exceeding 30.0 mmol/L or being lower than 11.1 mmol/L were excluded. Subsequently, based on the blood glucose data, all the model mice were grouped in an S-shaped manner, and they were classified as follows: model, metformin, RS-H, RS-M, RS-L, SF-RS-H, SF-RS-M, and SF-RS-L. Additionally, the mortality rate of the animals during the modeling period was recorded in detail.

The mice received the corresponding interventions by oral gavage once daily for 4 consecutive weeks. The control and model groups received an equivalent volume of vehicle. The positive control group received metformin at 300 mg/kg. The RS-treated groups received RS at 1.8, 0.9, and 0.45 g/kg, respectively, corresponding to the high-, medium-, and low-dose groups. Similarly, the SF-RS-treated groups received SF-RS at 1.8, 0.9, and 0.45 g/kg, respectively, corresponding to the high-, medium-, and low-dose groups. The doses were selected with reference to the clinically recommended dosage of *Raphani Semen*. According to the Chinese Pharmacopoeia, the recommended dose is 5–12 g/day. Taking the upper clinical dose of 12 g/day and a standard adult body weight of 60 kg gives a human dose of 0.2 g/kg. Based on the body-surface-area equivalent-dose conversion coefficients used in the present study, the corresponding doses were calculated as 1.4 g/kg for rats and 1.8 g/kg for mice. Thus, 1.8 g/kg was used as the high dose in mice, and the medium and low doses were set at 0.9 and 0.45 g/kg, respectively, at a ratio of 4:2:1.

Body weight, food intake, and fasting blood glucose were recorded once weekly throughout the intervention period. At the end of the 4-week treatment period, animals were fasted overnight with free access to water and then anesthetized with pentobarbital sodium. Blood samples were collected and centrifuged (4 °C, 4000 rpm, 10 min) to obtain serum. Liver tissues were rapidly excised, rinsed with ice-cold physiological saline, blotted dry, and divided into two portions. One portion was fixed in 4% paraformaldehyde for histopathological analysis, whereas the remaining tissue was snap-frozen and stored at −80 °C for subsequent biochemical and molecular assays [23].

### 2.8. Histological Analysis (HE, Oil Red O, Masson, PAS Staining of Liver Tissues)

HE staining: Mouse liver tissues were processed for routine histopathological examination according to standard fixation, embedding, and sectioning procedures. Paraffin-embedded liver sections were deparaffinized in environmentally friendly dewaxing solution I and II for 20 min each, followed by sequential rehydration in absolute ethanol I, absolute ethanol II, and 75% ethanol for 5 min each. After rinsing with tap water, sections were treated with the H&E pretreatment solution for 1 min and stained with hematoxylin for 3–5 min. The sections were then rinsed, differentiated, blued, and washed under running water. Eosin counterstaining was performed after brief dehydration in 95% ethanol for 1 min, followed by staining in eosin solution for 15 s. Finally, the sections were dehydrated through graded absolute ethanol, cleared in n-butanol and xylene, mounted with neutral resin, and examined under a light microscope for image acquisition.

Oil red O staining: Oil Red O staining was performed on frozen liver sections to detect neutral lipid accumulation. OCT-embedded frozen liver sections were removed from −20 °C storage, equilibrated to room temperature, and air-dried. The Oil Red O working solution was prepared by mixing six parts saturated Oil Red O solution with four parts distilled water, followed by overnight incubation at 4 °C and filtration before use. Sections were incubated with freshly filtered Oil Red O working solution for 8–10 min in the dark. Background staining was differentiated by sequential immersion in two changes of 60% isopropanol for 3 s and 5 s, respectively, followed by brief rinsing in distilled water. Nuclei were counterstained with hematoxylin for 0.5–3 min, differentiated for 2–8 s, blued briefly, and rinsed with tap water. The sections were mounted with glycerol gelatin mounting medium and imaged under a light microscope. Lipid droplets were identified by orange-red to bright-red staining, with nuclei counterstained blue.

Masson staining: Masson’s trichrome staining was performed to assess collagen deposition in liver tissue. Paraffin sections were deparaffinized and rehydrated sequentially in environmentally friendly dewaxing solution I and II for 15 min each, absolute ethanol I and II for 5 min each, and 75% ethanol for 5 min, followed by rinsing in tap water. The sections were incubated in Masson solution A at 65 °C for 30 min and washed with running water. Masson solutions D and F were prewarmed in a 55 °C water bath before use. Sections were stained with an equal-volume mixture of Masson solutions B and C for 1 min, briefly differentiated, and then stained with Masson solution D for 6 min. After rinsing, the sections were incubated in Masson solution E for 1 min and directly stained with Masson solution F for 2–30 s without water washing. Differentiation was performed with 1% acetic acid, followed by dehydration in absolute ethanol, clearing in xylene, and mounting with neutral resin. Collagen fibers were visualized in blue, whereas muscle fibers, fibrin, and erythrocytes appeared red.

PAS staining: PAS staining was used to visualize hepatic glycogen in liver tissues. Liver sections were deparaffinized and rehydrated as described above. After rinsing, sections were incubated with PAS staining solution B for 15 min, washed with tap water and distilled water, and then treated with PAS staining solution A for 30 min in the dark. After washing under running water for 5 min, nuclei were counterstained with PAS staining solution C for 30 s. The sections were subsequently differentiated in hydrochloric acid solution, blued in ammonia solution, rinsed, dehydrated through absolute ethanol, cleared in xylene, and mounted with neutral resin. PAS-positive structures appeared magenta to purplish red, whereas nuclei were lightly stained blue.

### 2.9. Biochemical Analysis

Serum biochemical indices were measured using commercially available assay kits according to the manufacturers’ instructions. Lipid metabolic parameters, including triglyceride (TG, A110-1-1), total cholesterol (TC, A111-1-1), low-density lipoprotein cholesterol (LDL-C, A113-1-1), and high-density lipoprotein cholesterol (HDL-C, A112-1-1), were determined to evaluate diabetes-associated dyslipidemia. Hepatic injury-related enzymes, including alanine aminotransferase (ALT, C009-2-1), aspartate aminotransferase (AST, C010-2-1), and alkaline phosphatase (ALP, A059-2-2), were measured to assess systemic metabolic liver stress [24,25].

To further evaluate renal safety, serum creatinine (Cr, C011-2-1), blood urea nitrogen (BUN, C013-3-1), and cystatin C (CysC, MM-0780M1) were measured in serum samples collected at the end of the intervention. Cr, BUN, and CysC concentrations were determined using commercially available assay kits according to the manufacturer’s instructions.

### 2.10. Oral Glucose Tolerance Test (OGTT), Insulin Level and Insulin Resistance Index Examination

OGTT was performed near the end of the intervention period to assess whole-body glucose handling capacity. After the final oral administration, animals were fasted for 12 h with free access to water. Baseline blood glucose was measured from the tail vein and recorded as the 0 min value. Subsequently, animals received an oral glucose load at 2 g/kg body weight using a freshly prepared 20% glucose solution. Blood glucose concentrations were then measured at 30, 60, and 120 min after glucose administration. The dynamic glucose excursion curve was used to evaluate the ability of each animal to dispose of an acute glucose challenge [26]. The area under the glucose curve was calculated to provide an integrated quantitative index of glucose tolerance.

Fasting serum insulin concentrations were determined using species-specific enzyme-linked immunosorbent assay kits (ELISA). Mice insulin was measured in serum samples from c57BL/6 mice. All assays were performed strictly according to the manufacturers’ protocols.

Insulin resistance (IR) was estimated using the homeostasis model assessment of insulin resistance. The insulin resistance index was calculated according to the following equation:

HOMA-IR = fasting blood glucose (mmol/L) × fasting insulin (mU/L)/22.5

This index was used as a surrogate indicator of systemic insulin resistance. In the present study, fasting glucose, fasting insulin, and HOMA-IR were jointly analyzed. This combined interpretation is particularly important in the T2DM model, in which changes in glucose levels alone may not fully distinguish between improved insulin action and altered insulin secretion [27].

### 2.11. Proteomics Analysis

Before conducting the experiment, we evaluated the results of the biochemical and pathological analyses. Finally, we selected representative samples from the control, model, RS-H, and SF-RS-H groups for subsequent analysis. Tissue proteins were extracted using an SDC-based protein extraction and digestion kit (Majorbio SDC ProPrep Kit, Majorbio, Shanghai, China). Briefly, tissue samples were ground for three cycles of 180 s each. The lysates were incubated at 95 °C for 5 min with shaking at 1500 rpm and then centrifuged at 14,000× *g* for 10 min. The supernatants were collected as total protein extracts. Protein concentration and sample quality were assessed using the BCA assay.

For peptide preparation, an aliquot of protein was selected according to the measured protein concentration. Each sample was adjusted to a final volume of 20 μL with SDC buffer. Proteins were reduced and alkylated using the kit-supplied reduction/alkylation reagent at 45 °C for 5 min with shaking at 1000 rpm, followed by enzymatic digestion with trypsin-containing digestion reagent at 37 °C for 4 h to overnight with shaking at 1000 rpm. The digests were acidified using the kit-supplied acidification reagent, during which visible precipitation formed. After centrifugation at 2000× *g* for 10 min, 150 μL of the peptide-containing supernatant was transferred to a new tube for desalting. Peptides were desalted using C18 StageTips. The eluates were dried by vacuum centrifugation and reconstituted in LC–MS loading buffer. Peptide concentration was determined by ultraviolet spectrophotometry using a NanoDrop One spectrophotometer (Thermo Scientific, Waltham, MA, USA).

Equal amounts of peptides from each sample were analyzed by data-independent acquisition liquid chromatography–tandem mass spectrometry (DIA-LC–MS/MS). Peptides were separated on a Vanquish Neo ultra-high-performance liquid chromatography system (Thermo Fisher Scientific) equipped with a homemade analytical column (15 cm × 100 μm, 1.7 μm). Mobile phase A consisted of 2% acetonitrile and 0.1% formic acid in water, and mobile phase B consisted of 80% acetonitrile and 0.1% formic acid. Chromatographic separation was performed at a flow rate of 2.6 μL/min using a 3.8 min gradient as follows: 8% B at 0 min, 10% B at 0.1 min, 10.5% B at 0.15 min, 28% B at 2.25 min, 50% B at 3.25 min, 99% B at 3.5 min, and 99% B at 3.8 min.

Mass spectrometric analysis was performed on an Astral Zoom mass spectrometer (Thermo Fisher Scientific) operated in positive-ion DIA mode, with data acquisition controlled by Thermo Xcalibur 4.7. The ion source voltage was set to 1.8 kV. MS1 spectra were acquired over an *m*/*z* range of 380–980, and MS/MS spectra were acquired over an *m*/*z* range of 150–2000. DIA acquisition was conducted using the predefined isolation-window scheme in the instrument method; the window table provided in the experimental report covered sequential *m*/*z* windows beginning at 380.422805–382.4237145 and ending at 550.5001125–552.501022. Raw data were recorded in Thermo raw format for subsequent database searching and quantitative proteomic analysis.

Proteomics data were processed using a standardized label-free quantitative workflow. RAW LC-MS/MS files were searched against the Uniprot protein database using Proteome Discoverer v3.3 software, with a peptide-spectrum match and protein-level false discovery rate of <1%. Protein abundance values were filtered to remove low-confidence identifications and features with excessive missingness, followed by log2 transformation and normalization. Differentially expressed proteins were defined according to false discovery rate (FDR)-adjusted *p* value < 0.05 and fold change > 1.5 or <0.67. Principal component analysis (PCA), hierarchical clustering, and volcano plots were used to evaluate proteomic variation among groups. Functional annotation of differentially expressed proteins was conducted using KEGG and GSEA analyses.

### 2.12. Metabolomics Analysis

Liver metabolomic profiling was performed using a UPLC-Orbitrap Fusion Lumos-MS/MS-based platform. Approximately 50 mg of liver tissue from each sample was accurately weighed. Metabolites were extracted by adding 300 μL of methanol/acetonitrile/water extraction solvent at a ratio of 2:2:1 (*v*/*v*/*v*), followed by thorough homogenization to ensure efficient disruption of liver tissue and release of intracellular metabolites.

The homogenates were vortexed for 1 min, then incubated at −20 °C for 2 h to facilitate metabolite extraction and protein precipitation. After incubation, the samples were centrifuged at 14,000 rpm for 10 min. The collected extracts were evaporated to dryness and subsequently reconstituted in 200 μL of acetonitrile. A pooled quality-control (QC) sample was prepared by combining equal aliquots (20 μL) from all individual extracts and was injected periodically (every 4 samples, inject one QC sample) throughout the analytical sequence to monitor system stability and data reproducibility. The specific LC-MS-related parameters were recorded in Table S2.

For metabolomics datasets, raw mass spectrometry files were processed for peak detection, deconvolution, alignment, normalization, and missing-value filtering before downstream analysis through Progenesis QI software (version 2.0). Features detected in less than 50% of samples within at least one experimental group or with a relative standard deviation greater than 30% in quality-control samples were excluded to ensure analytical reliability. The remaining variables were log-transformed according to the distribution characteristics of the dataset. Unsupervised PCA was first used to evaluate intrinsic clustering patterns, analytical stability, and the separation tendency among groups. Supervised orthogonal partial least-squares discriminant analysis (OPLS-DA) was subsequently performed to further characterize inter-group metabolic discrimination. Model fitness and predictive ability were assessed using R^2^X, R^2^Y, and Q^2^ values, and model overfitting was examined by permutation testing with 200 iterations. Differential metabolites were selected according to a combined criterion of variable importance in projection (VIP) > 1.0, fold change > 1.5 or <0.67, and FDR-adjusted *p* value < 0.05. Metabolite annotations were assigned according to the Metabolomics Standards Initiative (MSI) confidence levels. Differential metabolites were identified by matching experimental MS/MS spectra against reference spectra in the Human Metabolome Database (HMDB) using Progenesis QI. Because the identifications were based on MS/MS spectral matching rather than authentic chemical standards, the reported metabolites were classified as MSI Level 2 (putatively annotated compounds). Pathway enrichment and topology analyses were performed using MetaboAnalyst 6.0 online website, and pathways with *p* < 0.05 and pathway impact values above 0.10 were considered biologically relevant [28].

### 2.13. 16S rRNA Sequencing

Gut microbial profiling was performed by 16S rRNA gene amplicon sequencing. Microbial genomic DNA was extracted from the collected gut microbiota samples using the CTAB-based extraction method. The integrity, purity, and concentration of the extracted DNA were assessed by agarose gel electrophoresis. Qualified DNA samples were diluted with sterile water to a final concentration of 1 ng/μL before downstream amplification.

The bacterial 16S rRNA gene was amplified using sample-specific barcoded primers targeting the V4 hypervariable region. For bacterial community profiling, the 515F/806R primer pair was used according to the sequencing region specified in the experimental workflow. PCR amplification was performed using Phusion^®^ High-Fidelity PCR Master Mix with GC Buffer (New England Biolabs, Ipswich, MA, USA) and a high-fidelity DNA polymerase to improve amplification accuracy and minimize sequence errors. PCR products were examined by 2% agarose gel electrophoresis. Amplicons with the expected fragment size were purified using magnetic beads and quantified. Purified PCR products from individual samples were pooled in equimolar amounts according to their concentrations. The pooled amplicons were further checked by 2% agarose gel electrophoresis, and the target bands were recovered using a Qiagen gel extraction kit (Qiagen, Venlo, The Netherlands).

Sequencing libraries were constructed using the TruSeq^®^ DNA PCR-Free Sample Preparation Kit (Illumina, San Diego, CA, USA). Library quality was evaluated by Qubit fluorometric quantification and quantitative PCR. Qualified libraries were subjected to paired-end sequencing on an Illumina NovaSeq 6000 platform using a PE250 sequencing strategy [29,30,31].

For gut microbiota sequencing data, α-diversity indices, including Chao1, observed species, Shannon, and Simpson, were calculated to evaluate microbial richness and community diversity. Differences in α-diversity among groups were analyzed using the Kruskal–Wallis test followed by Dunn’s post hoc test. β-diversity was assessed based on Bray–Curtis, weighted UniFrac, and unweighted UniFrac distances, and visualized by principal coordinates analysis (PCoA) or non-metric multidimensional scaling (NMDS). Group-level differences in microbial community structure were tested using permutational multivariate analysis of variance (PERMANOVA) with 999 permutations. Differentially abundant taxa were identified using linear discriminant analysis effect size (LEfSe), Wilcoxon rank-sum test, or DESeq2 analysis, as appropriate, with an LDA score threshold of 4.0 and FDR-adjusted *p* value < 0.05. Predicted microbial functions were inferred using PICRUSt2, and functional pathways showing significant differences between groups were further interpreted in relation to host metabolic alterations.

### 2.14. Immunohistochemistry

Immunohistochemical staining was performed on liver tissue sections using an HRP-DAB detection system. Paraffin sections were deparaffinized in environmentally friendly dewaxing solution I, II, and III for 10 min each and rehydrated through three changes in absolute ethanol for 5 min each, followed by distilled water washing. Antigen retrieval was performed using the appropriate retrieval buffer according to the antibody information table (as listed in Table S3), and sections were allowed to cool naturally. After washing three times in PBS for 5 min each, endogenous peroxidase activity was blocked with 3% methanol-hydrogen peroxide solution for 25 min at room temperature in the dark. Sections were then washed and blocked with 3% BSA for 30 min at room temperature.

After removal of the blocking solution, sections were incubated with the indicated primary antibodies diluted in PBS overnight at 4 °C in a humidified chamber. The following day, slides were washed three times with PBS and incubated with the corresponding HRP-conjugated secondary antibody for 50 min at room temperature. DAB substrate was freshly prepared and applied to the sections, and color development was monitored microscopically. The reaction was terminated by rinsing with tap water once brown-yellow positive staining became visible. Sections were counterstained with hematoxylin for approximately 3 min, differentiated, blued, dehydrated through graded ethanol and n-butanol, cleared in xylene, and mounted with neutral resin. Images were acquired using a light microscope, and positive immunoreactivity was defined as brown-yellow staining.

### 2.15. Immunofluorescence

Immunofluorescence staining of paraffin-embedded liver sections was performed using a tyramide signal amplification-based protocol. Sections were deparaffinized in environmentally friendly dewaxing solutions I, II, and III for 10 min each and rehydrated through absolute ethanol, followed by distilled water washing. Antigen retrieval was carried out in the appropriate retrieval buffer according to the antibody-specific conditions (as listed in Table S4), and sections were cooled naturally before washing three times in PBS. Endogenous peroxidase activity was quenched with 3% hydrogen peroxide for 25 min at room temperature in the dark. After washing, sections were blocked with 3% BSA for 30 min.

For the first round of staining, sections were incubated with the first primary antibody overnight at 4 °C in a humidified chamber, washed with PBS, and incubated with the corresponding HRP-conjugated secondary antibody for 50 min at room temperature. TSA fluorophore working solution was then applied for 10 min in the dark. After washing in TBST, the slides were subjected to microwave treatment in antigen retrieval buffer to remove non-covalently bound antibodies while preserving the TSA signal. For the second round of staining, sections were incubated with the second primary antibody overnight at 4 °C, followed by the corresponding fluorescent secondary antibody for 50 min at room temperature in the dark. Nuclei were counterstained with DAPI for 10 min. Tissue autofluorescence was quenched when required, and sections were mounted with anti-fade mounting medium. Fluorescence images were acquired using a fluorescence microscope. DAPI was detected in the blue channel, whereas target signals were visualized using the corresponding Cy5 fluorescence channels.

### 2.16. Statistical Analysis

All experiments were performed using independent biological replicates, and the exact sample size for each assay was indicated in the corresponding figure legends (Table S5). Unless otherwise stated, quantitative data are presented as the mean ± standard deviation (SD). For comparisons between two independent groups, normally distributed data with equal variance were analyzed using an unpaired two-tailed Student’s *t*-test, whereas data that did not meet the assumptions of normality were evaluated using the Mann–Whitney U test. For comparisons among multiple groups, one-way analysis of variance (ANOVA) followed by Dunnett’s post hoc test was used when each treatment group was compared with a single control or model group. For direct comparison of raw and stir-fried *Raphani Semen* across the three matched dose levels, data from the RS and SF-RS treatment groups were analyzed using two-sided Welch’s *t*-tests. Then, the Holm step-down procedure was applied to adjust for multiple testing and control the family-wise error rate. Statistical significance for these comparisons is determined on the basis of the Holm-adjusted *p*-values, with an adjusted *p*-value lower than 0.05 considered statistically significant. For parameters measured repeatedly over time, including body weight, fasting blood glucose, or oral glucose tolerance test, two-way repeated-measures ANOVA was conducted, followed by Bonferroni’s or Šídák’s post hoc correction where appropriate. Non-parametric multi-group comparisons were performed using the Kruskal–Wallis test followed by Dunn’s multiple-comparison test. Correlation between key metabolites, crucial proteins, and microbial taxa was analyzed using Spearman’s correlation analysis. Correlation matrices were visualized as heatmaps, and only correlations with |r| > 0.8 and adjusted *p* < 0.05 were considered statistically meaningful.

All statistical analyses were conducted using GraphPad Prism (version 10.0), SPSS (version 26.0), SIMCA (version 14.0), and R software (version 4.3.3), depending on the dataset type. Data visualization was performed using GraphPad Prism, R packages, and SIMCA software. A two-sided *p* value < 0.05 was considered statistically significant. Statistical significance was denoted as follows: * *p* < 0.05, ^#^ *p* < 0.05, **p < 0.01, ^##^ *p* < 0.01, unless otherwise specified.

## 3. Results

### 3.1. Integrated Chromatographic and Macroscopic Assessment Supports Selection of 160 °C for 3 Min as a Representative Stir-Frying Condition

**Figure 2:**
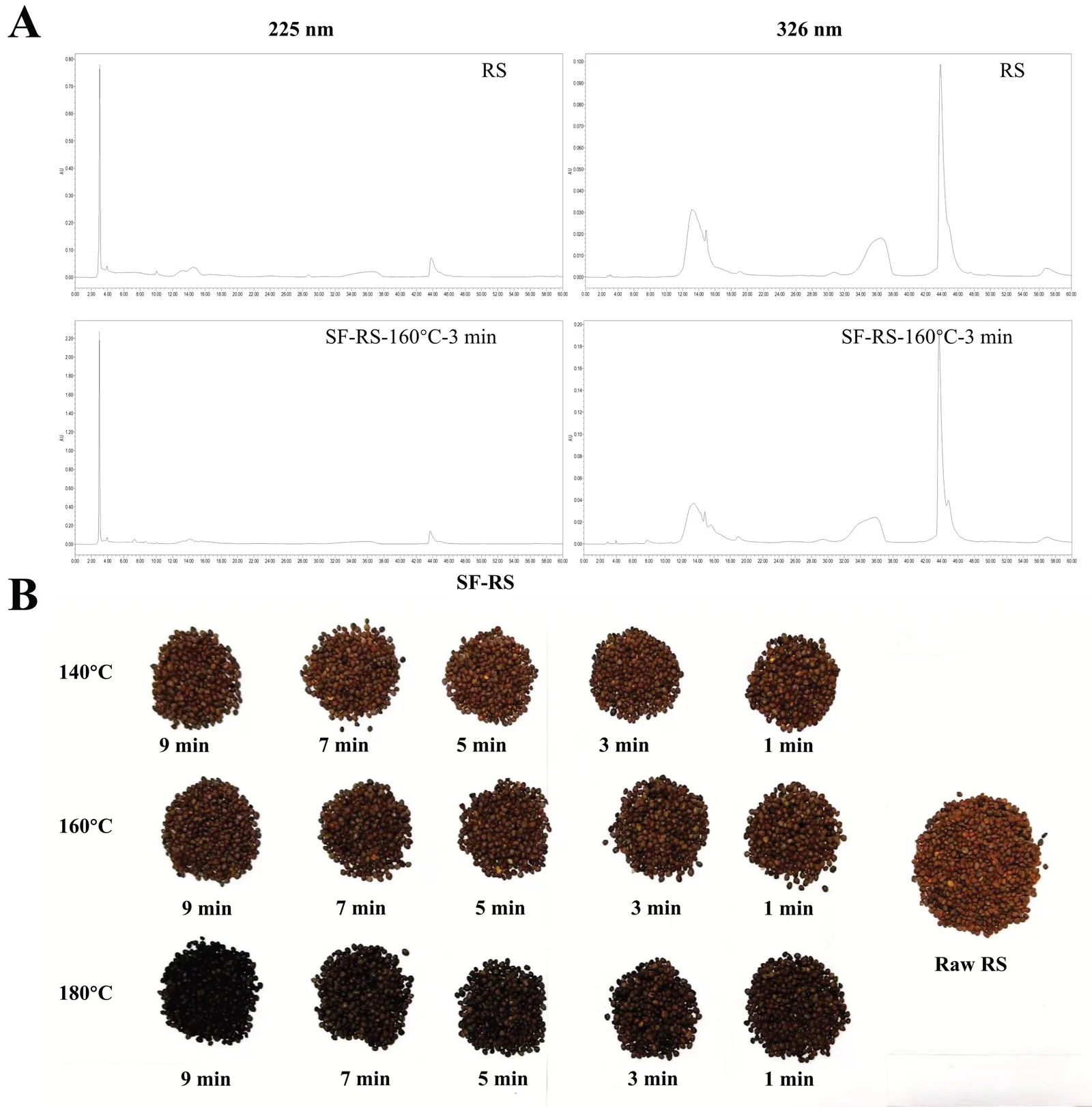
Exploratory screening of stir-frying conditions for *Raphani Semen*. (**A**) Representative HPLC chromatograms of RS and SF-RS samples at wavelengths of 225 nm and 326 nm. (**B**) Representative photographs of the RS samples under different stir-frying times and temperatures.

Exploratory screening of the 15 temperature–time combinations showed progressive changes in both the chromatographic profile and the physical characteristics of RS as processing intensity increased (Figure 2 and Supplementary Figures S1 and S2). At 140 °C, the overall appearance remained close to that of the raw material, although changes in the intensity of several UV-absorbing signals were already evident. Increasing the temperature and/or heating duration produced greater redistribution of chromatographic responses, particularly at 225 nm. The 140 °C/3 min sample showed a pronounced response at 326 nm, whereas prolonged treatment at 180 °C progressively weakened the dominant signal and generated additional low-intensity peaks. The most intensive condition, 180 °C for 9 min, was accompanied by broad attenuation and distortion of the chromatographic profile, consistent with excessive thermal processing.

The macroscopic and organoleptic observations showed a parallel progression (Figure 2B and Table S6). Raw RS had a heterogeneous reddish-brown surface. Samples processed at 140 °C showed only limited darkening, whereas treatment at 160 °C produced a more uniform brown-to-dark-brown appearance while largely preserving seed integrity. Further increases in processing intensity at 180 °C resulted in pronounced surface darkening, and the samples heated for 5–9 min showed characteristics inconsistent with the intended stir-fried endpoint, including brown-black coloration and stronger burnt characteristics. The assessor-based evaluation further indicated that the 160 °C/3 min sample exhibited moderate surface darkening, slight swelling, increased crispness, and a mild roasted aroma without evident charring or a pronounced burnt odor.

When the predefined physical characteristics were considered together with the exploratory HPLC profiles, 160 °C for 3 min was selected as the representative condition for preparation of SF-RS in the subsequent experiments. Under this condition, the material exhibited the expected stir-fried characteristics while retaining the principal chromatographic signals of raw RS and avoiding the extensive darkening and signal attenuation associated with more intensive heating. Because each temperature–time combination was represented by a single independent processing run, this selection should be interpreted as an exploratory identification of a representative condition within the tested range rather than as formal optimization of the stir-frying process.

The reproducibility of the stir-frying procedure was evaluated using three independently prepared SF-RS batches produced under the same representative processing conditions (160 °C for 3 min). Across the three batches (Table S7), the surface color, crispness, roasted aroma, and burnt odour were consistent with the original preparation. The content of the representative chemical marker sinapine (based on the China Pharmacopoeia, 2020 edition) varied from 5.1 to 5.5 mg/g, complying with the standards of the China Pharmacopoeia. In addition, the chromatographic profiles of the three batches were highly comparable, with fingerprint similarity values of 0.92–0.96, indicating acceptable batch-to-batch consistency under the applied processing conditions. A more comprehensive characterization of the processing reproducibility will be reported separately.

### 3.2. Stir-Frying Reconfigures the Chemical Composition of RS and Selectively Constrains Its Serum-Exposed Constituent Repertoire

#### 3.2.1. Stir-Frying Preserves Broad Chemical-Class Coverage While Redistributing the Abundance of Major Chemical Constituents in RS

**Figure 3:**
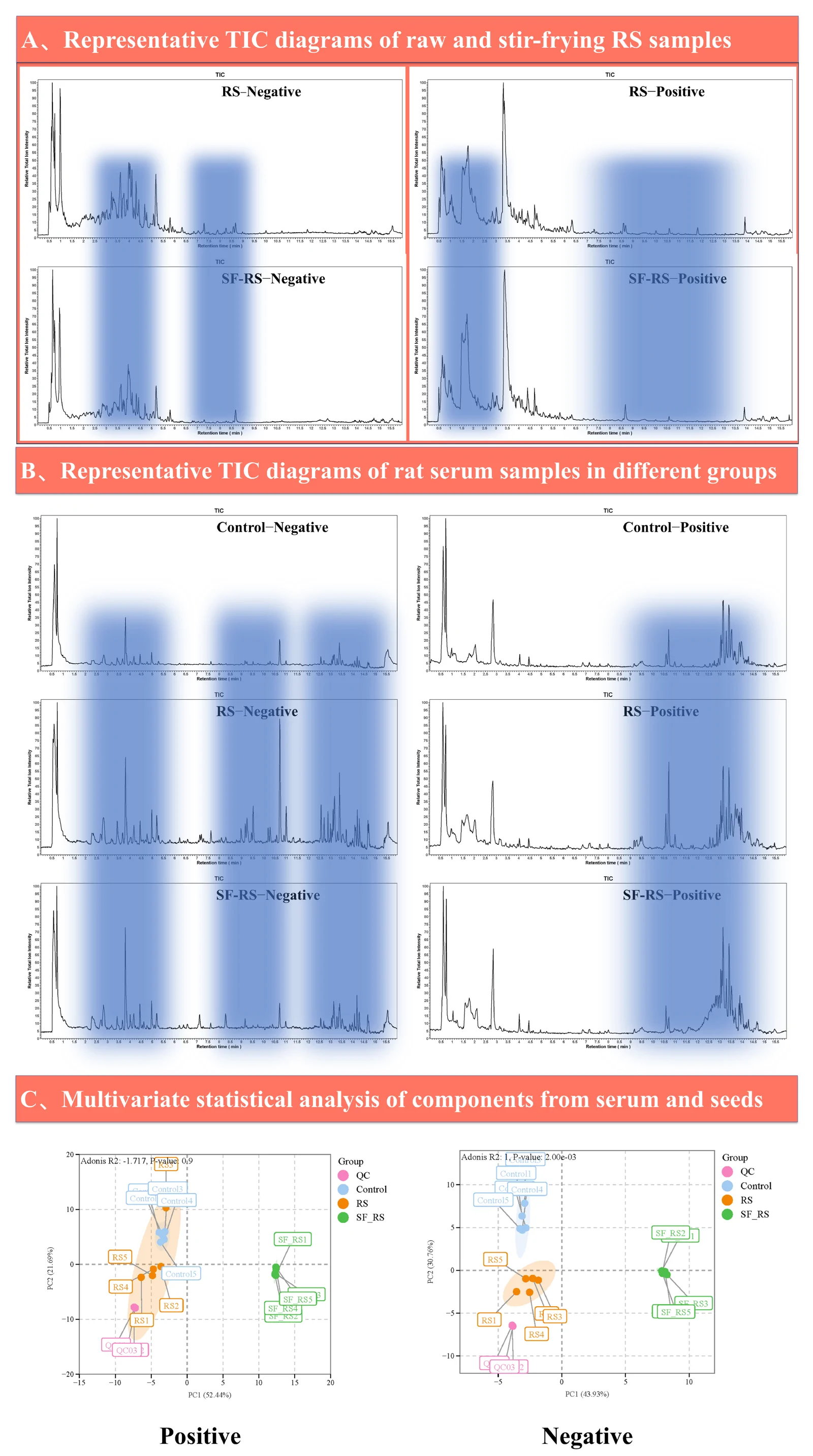
Stir-frying reshapes the chemical profile and serum-detectable constituent of *Raphani Semen* (n = 5). (**A**) Representative TICs of raw *Raphani Semen* and stir-fried *Raphani Semen* extracts acquired by UPLC-Q-Exactive-MS. (**B**) Representative TICs of blank control serum and RS- and SF-RS-treated serum collected from rats. (**C**) PCA score plots of different samples.

Representative total-ion chromatograms (TICs) of RS and SF-RS showed similar overall profiles in positive- and negative-ion modes, although the relative intensities of individual signals differed (Figure 3A). In negative-ion mode, RS showed a dense group of peaks in the early and middle retention-time regions, several of which had lower intensities after stir-frying. In positive-ion mode, the main early-eluting signals remained detectable after processing, but peak intensities changed across both early and late retention times. These chromatographic profiles suggest that stir-frying retained the broad chemical pattern of RS while changing the relative responses of specific features.

Feature-level annotation was consistent with this visual interpretation. UPLC–Q–Exactive–MS profiling yielded 772 and 780 annotated features in RS (as listed in Table S8) and SF-RS (as exhibited in Table S9), respectively, of which 748 were shared between the two preparations. Only 24 features were detected exclusively in the raw material and 32 exclusively in the stir-fried material. The modest difference in overall feature richness therefore contrasted with a much broader redistribution of signal abundance. Among the shared features, 104 showed relative LC-MS response ratios ≥ 2.0 in SF-RS compared with RS, whereas 104 showed ratios ≤ 0.5. These changes represent differences in relative analytical signal intensity and should not be interpreted as absolute concentration changes because compound-specific calibration was not performed.

At the class level, processing-associated changes were heterogeneous. Several glucosinolate-, lipid-, and terpenoid-related features showed higher relative LC-MS responses in SF-RS, whereas a larger proportion of amino-acid-, nucleotide-, phenolamide-, and flavonoid-related features showed lower relative responses. Because these observations were derived from non-targeted LC-MS peak areas without compound-specific calibration, they are interpreted as relative analytical response patterns rather than quantitative changes in total class concentrations.

Collectively, the TIC profiles and comprehensive annotation matrix demonstrate that stir-frying preserved the principal chemical-class architecture of RS while extensively altering the relative weighting of its constituent families. The processed material was characterized not by a wholesale gain or loss of chemical diversity, but by a transition from a profile richer in amino-acid-, nucleotide- and flavonoid-related responses towards one with greater contributions from glucosinolate-associated, lipid, terpenoid, carbohydrate and steroid-like features. This class-level reorganization provides the chemical context required to interpret the more selective changes subsequently observed in the serum-detectable constituent profile.

#### 3.2.2. RS and SF-RS Produce Distinct Serum-Detectable Chromatographic Feature Profiles

Representative serum chromatograms provided evidence that the serum-detectable chemical feature profile differed between RS- and SF-RS-treated animals (Figure 3B). Relative to blank serum, both administration groups exhibited additional or enhanced chromatographic signals in the early-, intermediate- and late-eluting regions. The differences were particularly evident in negative-ion mode, in which serum obtained after administration of raw RS displayed a denser and more complex distribution of signals than serum obtained after administration of the stir-fried material. In positive-ion mode, both administration groups exhibited prominent late-eluting signal clusters, although their relative intensities and peak distributions differed. Because total-ion chromatograms contain both endogenous and herb-derived ions, these visual differences were interpreted descriptively and were subsequently resolved by blank-serum filtering and cross-matching against the corresponding herbal extracts.

#### 3.2.3. Stir-Frying Selectively Reshapes the Serum-Detectable Profile of RS

Unsupervised multivariate analysis supported an overall processing-dependent shift in the serum chemical profile (Figure 3C). The positive-ion data showed clear separation among blank serum, RS-containing serum and SF-RS-containing serum, with biological replicates occupying distinct regions of the score space. Separation was less complete in negative-ion mode, particularly between the blank-serum and RS groups, whereas the SF-RS group remained visibly displaced along the principal axis. The two ionization modes therefore provided complementary information: positive-ion features contributed more strongly to global group discrimination, while negative-ion acquisition expanded the coverage of acidic and highly polar constituents.

Following blank-serum filtering and cross-matching against the corresponding administered extracts, 173 serum-detectable constituents after RS administration and 77 after SF-RS administration fulfilled the predefined criteria for putatively annotated prototype constituents (Tables S10 and S11). For each retained prototype, the serum feature could be matched to a constituent in the corresponding RS or SF-RS extract on the basis of compound annotation, accurate precursor *m*/*z*, formula, retention time, ionization characteristics, and MS/MS fragmentation information. Importantly, serum-associated signals were not classified as prototypes solely on the basis of their occurrence or enrichment after administration. Features without a corresponding constituent in the administered extract were evaluated separately as potential biotransformation products. The raw serum dataset additionally contained several features consistent with Phase-II conjugation products of phenolic constituents, including candidate glucuronide and sulfate conjugates of sinapic acid, caffeic acid, and isoferulic acid. These features were therefore separated from the prototype list and conservatively annotated as putative metabolites pending further confirmation of their diagnostic MS/MS fragmentation.

#### 3.2.4. Material-Serum Integration Identifies Candidate Glucosinolate-Related Processing Signals

Cross-matrix comparison showed that only a subset of the relative LC-MS changes observed in the administered materials was paralleled by serum-detectable feature patterns. Butenyl glucosinolate- and glucoraphanin-associated features showed higher relative signals in both SF-RS and the corresponding serum samples, whereas several phenolamide-, flavonoid-, and sinapoyl-related features showed lower relative signals or detection frequencies after SF-RS administration. These observations identify candidate processing-associated chemical features but do not establish absolute differences in constituent concentrations, systemic exposure, or absorption. Targeted quantitative and pharmacokinetic analyses will be required to determine whether these candidates contribute to the pharmacological differences between RS and SF-RS.

### 3.3. Stir-Frying Preferentially Enhances Glycemic Regulation and Broadens Selected Metabolic Responses of RS in T2DM Mice

A total of 72 animals entered the current study, of which 64 received STZ and HSHFD. During the induction and screening period, no animals died, as recorded in Table S12. Similarly, before the administration of the intervention, no experimental animals were excluded because, based on the results of two consecutive blood glucose tests (as listed in Table S13), no mice showed values lower than 11.1 mmol/L or higher than 30.0 mmol/L, which were within the exclusion criteria we had set.

**Figure 4:**
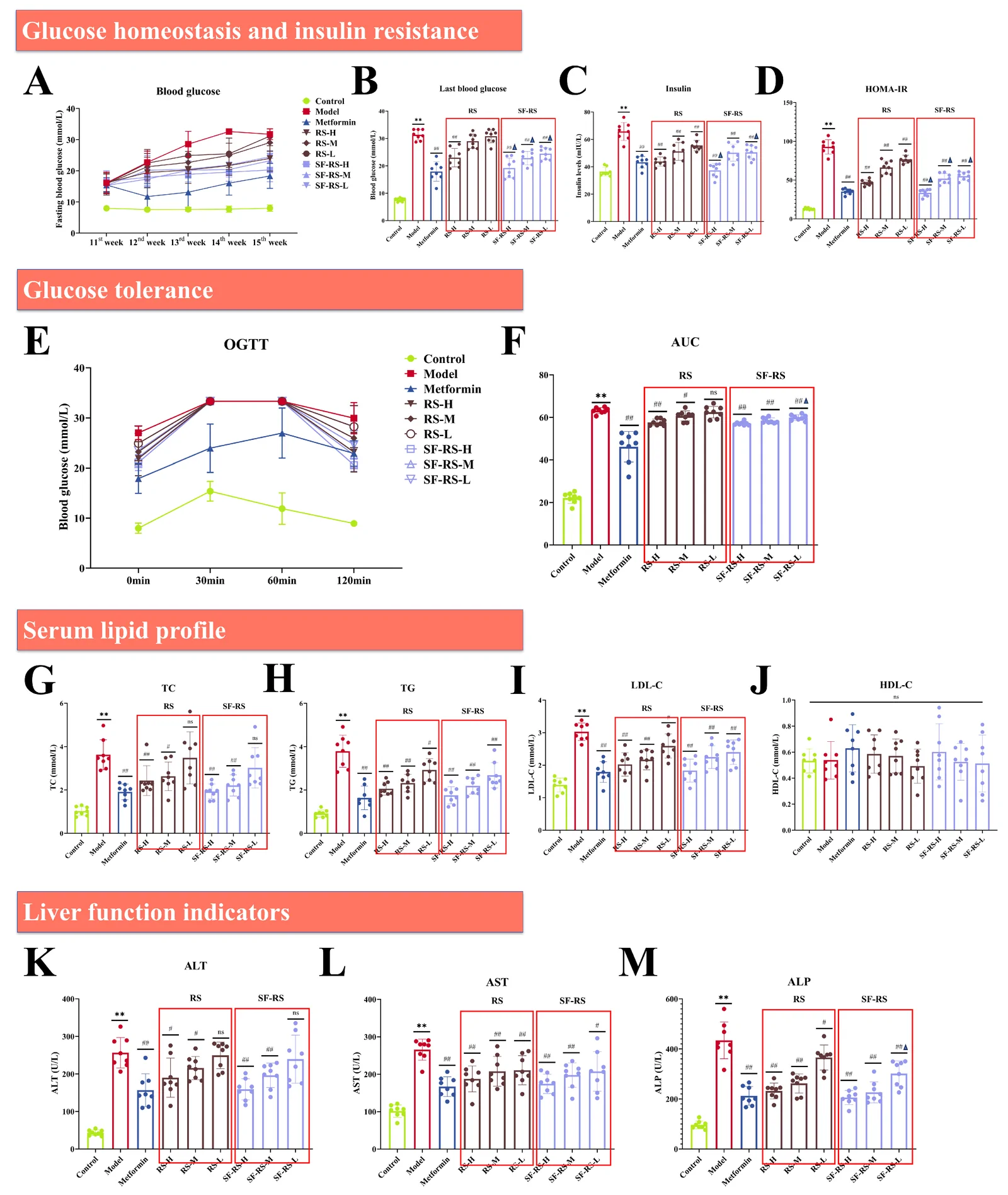
RS and SF-RS treatments improve glucose homeostasis, insulin resistance, serum lipid profiles, and liver function in T2DM mice (n = 8). (**A**) Changes in fasting blood glucose during the intervention. (**B**) Terminal fasting blood glucose levels. (**C**) Serum insulin concentrations. (**D**) HOMA-IR. (**E**) Blood glucose concentrations during the OGTT. (**F**) AUC of the OGTT. (**G**–**J**) Serum concentrations of TC, TG, LDL-C and HDL-C, respectively. (**K**–**M**) Serum activities of ALT, AST and ALP, respectively. Data are presented as mean ± SD, ** *p* < 0.01 versus control, # *p* < 0.05, ## *p* < 0.01 versus model, ns, not significant. The triangle indicates that, compared with the RS group, the *p* value of the SF-RS group is less than 0.05.

The diabetic model developed a sustained disturbance in glucose homeostasis, as reflected by the progressive elevation of fasting blood glucose during the intervention period and the marked increases in terminal fasting glucose, serum insulin, and HOMA-IR compared with the control group (*p* < 0.01, Figure 4A–D, Table S14). Administration of either RS or SF-RS attenuated these abnormalities, with greater responses generally observed at higher doses. Importantly, the matched-dose comparisons between the two preparations revealed a consistent processing-related difference. Terminal fasting glucose was significantly lower in the SF-RS-H, SF-RS-M, and SF-RS-L groups than in the corresponding RS-H, RS-M, and RS-L groups, respectively (*p* < 0.05, Figure 4B, Table S14). The same pattern was observed for serum insulin and HOMA-IR, for which SF-RS produced significantly lower values than raw RS at each of the three dose levels (*p* < 0.05, Figure 4C,D, Table S14). The high-dose SF-RS group also showed terminal glycemic and insulin-resistance indices approaching those observed in metformin-treated mice. These direct comparisons indicate that stir-frying strengthened the effects of RS on fasting glycemia and insulin resistance across the tested dose range.

Glucose tolerance was further assessed by OGTT (Figure 4E,F). Model mice exhibited a pronounced and sustained glucose excursion after glucose loading, whereas both RS and SF-RS promoted glucose clearance during the later phase of the test. Consistent with this response, high- and medium-dose RS significantly reduced the OGTT AUC relative to the model group (*p* < 0.01 and *p* < 0.05, respectively, Table S14), while the effect of low-dose RS was not significant (*p* > 0.05). In contrast, significant reductions in AUC were observed at all three SF-RS doses (*p* < 0.01 versus the model group, Table S14). Direct comparison of the matched doses showed a significant difference between SF-RS-L and RS-L (*p* < 0.05, Table S14), whereas the differences at the high and medium doses did not reach statistical significance. Thus, the principal processing-related advantage in glucose tolerance was evident at the low dose, at which stir-frying preserved an effect that was not detected with raw RS.

Diabetes induction was also accompanied by substantial dyslipidemia. Serum TC, TG, and LDL-C were significantly elevated in model mice relative to controls (*p* < 0.01), whereas HDL-C did not differ significantly among the experimental groups (Figure 4G–J, Table S14). RS reduced TC at the high and medium doses, while the low dose had no significant effect. A similar pattern was observed after SF-RS treatment, with significant reductions at the high and medium doses but not at the low dose. For TG and LDL-C, RS produced significant decreases across the three doses, although the magnitude of significance was weaker at the low dose. SF-RS, by comparison, significantly reduced both TG and LDL-C at all three dose levels (*p* < 0.01 versus the model group, Table S14). Despite this more uniform response to SF-RS relative to the diabetic model, none of the matched RS–SF-RS comparisons for TC, TG, LDL-C, or HDL-C reached statistical significance (*p* > 0.05, Table S14). The lipid data therefore support a broader response to the processed preparation across doses, but do not establish a general superiority of SF-RS over RS for individual circulating lipid parameters.

Changes in serum liver-associated enzymes were consistent with the hepatic metabolic stress induced by the diabetic model (Figure 4K–M, Table S14). ALT, AST, and ALP activities were all markedly increased in model mice compared with controls (*p* < 0.01, Table S14). RS significantly decreased ALT at the high and medium doses, whereas the low dose was ineffective. SF-RS also reduced ALT at the high and medium doses, with no significant response at the low dose. AST activity was lowered by all doses of both preparations, although the statistical strength of the response varied among doses. No significant differences in ALT or AST were detected between RS and SF-RS at matched dose levels. For ALP, all three RS doses reduced the model-associated elevation, whereas SF-RS produced significant reductions at each dose at *p* < 0.01. Notably, direct comparison at the low dose showed significantly lower ALP activity in SF-RS-L than in RS-L (*p* < 0.05, Table S14), while the high- and medium-dose pairs did not differ significantly.

Body weight and food intake were monitored weekly throughout the experimental period (Figure S3). During model establishment, the high-fat diet/STZ-induced groups exhibited a progressive increase in body weight, with broadly comparable body-weight trajectories among the model and subsequent treatment groups. During the treatment period, the body weight of the model group decreased slightly. In contrast, no pronounced body-weight loss was observed following metformin, RS, or SF-RS administration. Food intake was generally comparable among the model and treatment groups before intervention. From week 11 onward, food intake increased progressively in the model group. This increase was less pronounced in the metformin, RS-treated groups, and SF-RS-treated groups. Together with the blood glucose results, these data showed that the improvement in glycemic control was not accompanied by a marked reduction in body weight, although treatment-dependent differences in food intake emerged during the intervention period.

To further assess renal safety, serum Cr, BUN, and CysC were determined at the end of the intervention (Supplementary Table S15). Compared with the Control group, the Model group showed significantly increased serum Cr (*p* < 0.01) and BUN (*p* < 0.01), whereas CysC did not differ significantly between the two groups (*p* = 0.801). Compared with the Model group, serum Cr and BUN were significantly lower in the Metformin, RS-H, RS-M, RS-L, SF-RS-H, SF-RS-M, and SF-RS-L groups (*p* < 0.01 for all comparisons), whereas CysC did not differ significantly between the Model group and any treatment group (all *p* > 0.05). At the high dose, neither RS-H nor SF-RS-H showed biochemical evidence of worsening renal function relative to the diabetic model group. In the prespecified dose-matched comparisons, Cr was significantly lower in SF-RS-H than in RS-H (*p* < 0.05), whereas the corresponding differences in BUN and CysC were not statistically significant.

### 3.4. Stir-Frying Enhances the Protective Effects of RS Against Hepatic Lipid Accumulation, Glycogen Depletion, and Collagen Deposition in T2DM Mice

**Figure 5:**
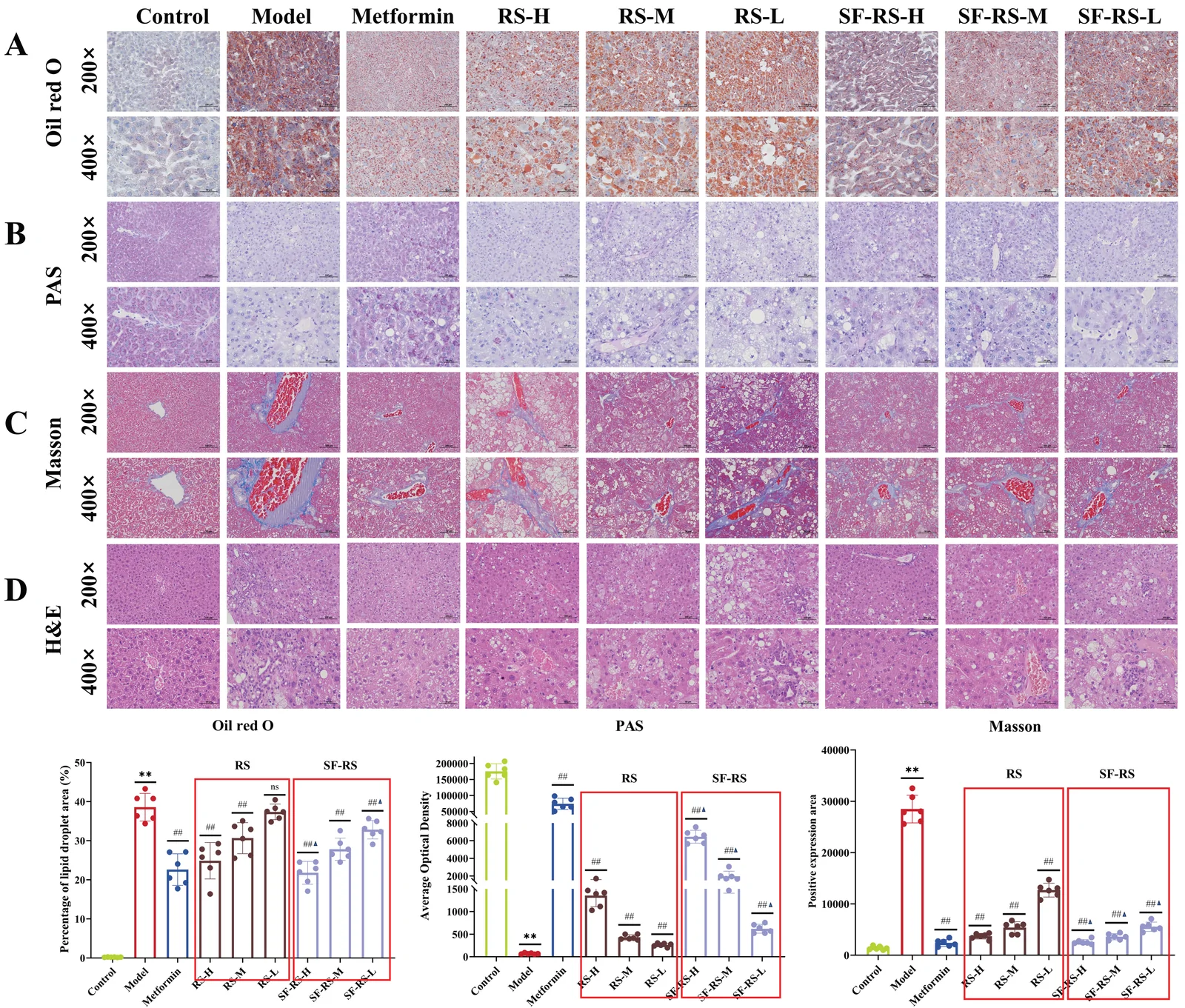
Raw and stir-fried *Raphani Semen* attenuate hepatic steatosis, glycogen depletion, collagen deposition, and histopathological injury in T2DM mice (n = 6, per group). (**A**) Representative oil red O-stained liver sections at 200× and 400× magnification. (**B**) PAS-stained liver sections at 200× and 400× magnification. (**C**) Representative Masson’s trichrome-stained liver sections at 200× and 400× magnification. (**D**) Representative H&E-stained liver sections at 200× and 400× magnification. Scale bars are indicated in the corresponding micrographs. Data are presented as the mean ± SD, ** *p* < 0.01 versus the control group; ## *p* < 0.01 versus the model group; ns, not significant. The triangle indicates that, compared with the RS group, the *p* value of the SF-RS group is less than 0.05.

Liver histology revealed pronounced structural and metabolic abnormalities in diabetic mice and showed differential responses to raw and stir-fried RS treatment (Figure 5). H&E staining of control liver sections showed preserved lobular architecture, regularly arranged hepatocytes, and minimal cytoplasmic vacuolation. In contrast, the model group exhibited extensive hepatocellular vacuolation, ballooning-like changes, disruption of hepatic cords, and prominent lipid-associated degeneration. Metformin markedly alleviated these histological abnormalities. Both RS and SF-RS improved hepatic morphology in a dose-related manner. Qualitatively, liver sections from SF-RS-treated mice showed less vacuolation and better preservation of hepatocyte organization than those from the corresponding RS groups, with the difference appearing more evident at the medium and low doses.

Oil Red O staining demonstrated a marked increase in hepatic neutral-lipid accumulation after T2DM induction (Figure 5A). The lipid-positive area reached approximately 38% in the model group, whereas only minimal staining was detected in control mice (*p* < 0.01). High- and medium-dose RS significantly reduced the lipid-positive area (*p* < 0.01 versus the model group), while the low dose produced no significant reduction. SF-RS reduced lipid accumulation at all three doses (*p* < 0.01 versus the model group). Matched-dose comparisons further showed that SF-RS-H produced a significantly smaller lipid-positive area than RS-H (*p* < 0.05). A significant processing-related difference was also observed at the low dose, where SF-RS-L reduced hepatic lipid accumulation relative to RS-L (*p* < 0.05). No significant difference was detected between the two medium-dose groups. These results indicate that stir-frying enhanced the lipid-lowering effect of RS at selected dose levels and, importantly, preserved significant activity at the low dose.

PAS staining showed a profound depletion of hepatic PAS-positive material in diabetic mice, with the model group exhibiting markedly lower staining intensity than the control group (*p* < 0.01; Figure 5B). Treatment with either preparation significantly increased the PAS signal relative to the model group at all tested doses (*p* < 0.01). However, the magnitude of recovery differed substantially between the raw and processed preparations. Average optical density values in the RS-H, RS-M, and RS-L groups were approximately 1.3 × 10^3^, 0.4 × 10^3^, and 0.25 × 10^3^, respectively, whereas the corresponding values after SF-RS treatment increased to approximately 6.5 × 10^3^, 2.0 × 10^3^, and 0.6 × 10^3^. Direct comparisons confirmed significantly higher PAS staining in SF-RS-H, SF-RS-M, and SF-RS-L than in their respective dose-matched RS groups (*p* < 0.05). Although none of the treatments restored the PAS signal to the level observed in control or metformin-treated mice, the consistent differences between matched preparations demonstrate that stir-frying strengthened the capacity of RS to counteract diabetes-associated hepatic glycogen depletion across the entire dose range.

Masson’s trichrome staining revealed extensive collagen accumulation in the livers of model mice (Figure 5C). The collagen-positive area increased in the model group (*p* < 0.01). Both RS and SF-RS significantly reduced collagen deposition relative to untreated diabetic mice at all three doses (*p* < 0.01). Matched-dose analysis showed significantly less collagen deposition in SF-RS-H than in RS-H and in SF-RS-L than in RS-L (*p* < 0.05), whereas the difference between the medium-dose groups was not statistically significant. The particularly marked reduction observed at the low dose indicates that processing increased the ability of RS to limit collagen accumulation under conditions in which the raw preparation showed a comparatively weaker response.

Collectively, the histological findings demonstrate that both RS preparations alleviated diabetes-associated hepatic injury, but stir-frying modified the magnitude and dose profile of these effects. The processing-related advantage was most consistent for PAS-positive glycogen restoration, for which SF-RS outperformed raw RS at all three matched doses. Additional significant improvements were observed for hepatic lipid accumulation and collagen deposition at the high and low doses. Together, these findings provide direct histological evidence that stir-frying enhances selected hepatoprotective actions of *Raphani Semen* rather than simply extending the range of responses observed relative to untreated diabetic mice.

### 3.5. Proteomic Changes Associated with RS and SF-RS Treatments in T2DM Mice

#### 3.5.1. Quality Assessment and Global Characteristics of the Proteomic Dataset

Before the between-group comparisons, the quality and reproducibility of the LC–MS/MS proteomic data were evaluated. Peptide length, peptide number, protein molecular weight, and sequence coverage showed the expected distributions (Supplementary Figure S3). These results were consistent with effective protein digestion, broad peptide identification, and adequate proteome coverage. Most identified proteins had measurable sequence coverage, supporting their use in subsequent quantitative analyses.

Protein-level coefficients of variation, hierarchical clustering, and pairwise sample correlations were then examined to assess consistency among biological replicates. Most quantified proteins showed relatively low within-group variation. Samples from the same experimental group generally clustered together and had high pairwise correlations, whereas samples from different groups showed distinct global abundance patterns (Supplementary Figure S4). These findings indicate that the dataset had good internal consistency and was suitable for differential protein abundance and pathway enrichment analyses.

#### 3.5.2. Proteomic Changes and Pathway Alterations Associated with RS and SF-RS Treatment

**Figure 6:**
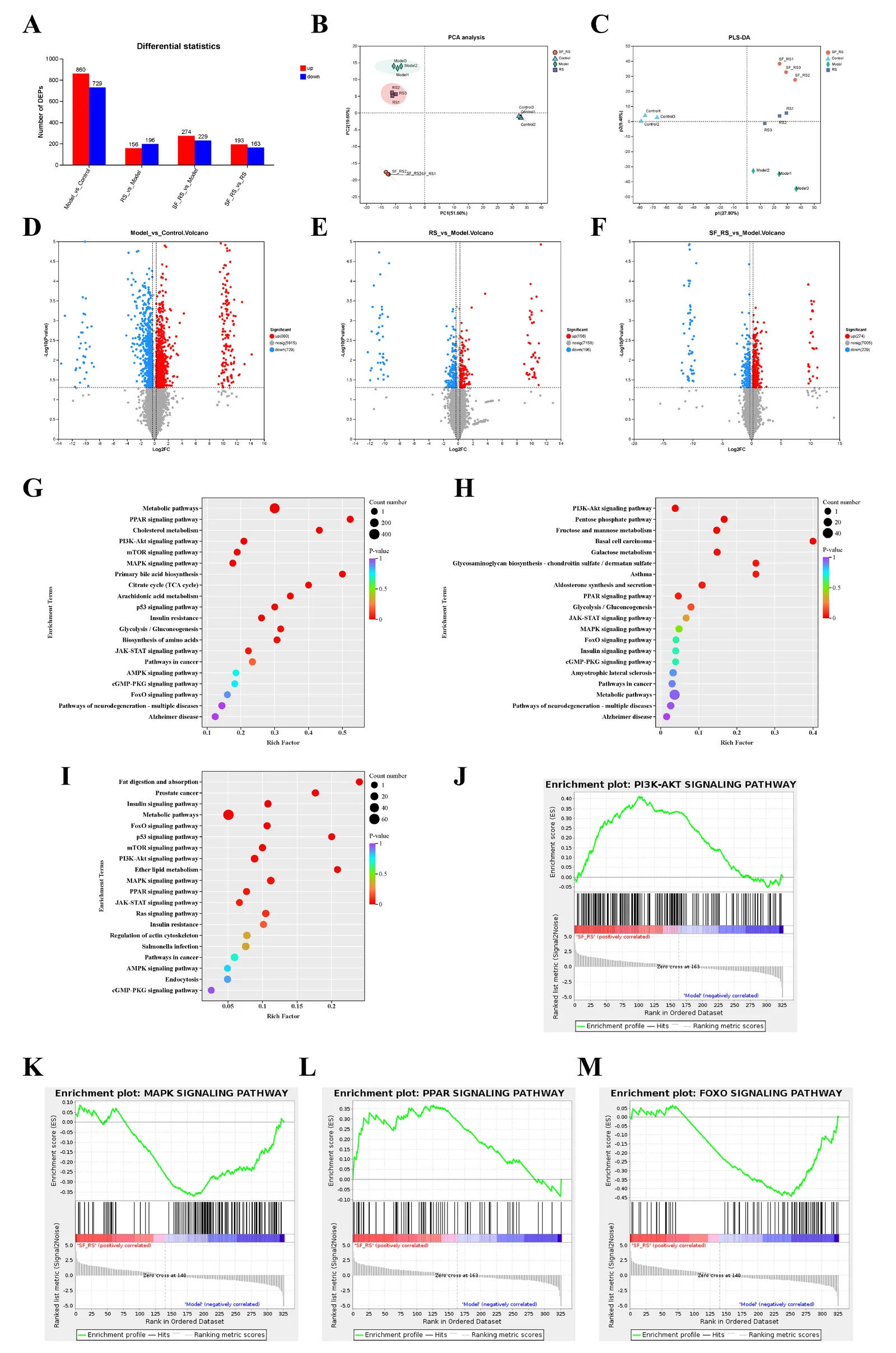
Multivariate analysis and functional enrichment of proteomic alterations induced by raw and stir-fried *Raphani Semen* in T2DM mice (n = 3). (**A**) Statistical analysis of the number of differential expressed proteins in different comparisons. (**B**) PCA illustrating global proteomic separation among control, model, RS, and SF-RS groups. (**C**) PLS-DA confirming clear group discrimination and treatment-dependent proteomic shifts. (**D**–**F**) Volcano plots showing differentially expressed proteins across experimental comparisons. (**G**–**I**) KEGG pathway enrichment analysis of control vs. model, model vs. RS, and model vs. SF-RS comparisons, respectively. (**J**–**M**) GSEA of the model vs. SF-RS comparison.

Multivariate analysis, differential protein abundance analysis, and pathway enrichment were used to characterize the hepatic proteomic changes associated with diabetes and the responses to raw and stir-fried RS. Principal component analysis (PCA) showed clear differences in the overall protein abundance profiles of the four groups (Figure 6B). PC1 and PC2 explained 38.6% and 21.4% of the total variance, respectively. Together, the first two components accounted for 60.0% of the variation in the dataset. The model group was separated from the control group mainly along PC1, indicating that diabetes induction was associated with broad changes in the hepatic proteome. Both treatment groups shifted away from the model group and toward the control group. The SF-RS samples were positioned closer to the control cluster than the RS samples, suggesting a larger shift toward a control-like proteomic profile after SF-RS treatment.

Partial least squares discriminant analysis (PLS-DA) was also used to examine treatment-related differences in protein abundance patterns (Figure 6C). The score plot separated the four experimental groups, with little overlap among them. The model samples were located at one end of the score space, whereas the RS- and SF-RS-treated samples shifted in the opposite direction. In agreement with the PCA pattern, the SF-RS group showed a larger descriptive shift toward the control group than the RS group. These results support the presence of distinct proteomic responses to raw and stir-fried RS. Differential protein abundance analysis further characterized the effects of diabetes and treatment (Figure 6A). Relative to the control group, the model group contained 860 proteins with higher abundance and 729 proteins with lower abundance, based on the predefined statistical criteria. This large number of altered proteins indicates that diabetes affected a wide range of hepatic metabolic and signaling processes. Raw RS shifted part of this diabetes-associated protein pattern in the opposite direction. A larger proportion of the altered proteins moved toward control levels after SF-RS treatment, suggesting that stir-frying was associated with a broader correction of the diabetes-related proteomic changes (Figure 6D–F).

KEGG enrichment analysis was then performed for the control-versus-model, model-versus-RS, and model-versus-SF-RS comparisons (Figure 6G–I). Proteins that differed between the control and model groups were enriched in pathways related to insulin resistance, glycolysis and gluconeogenesis, and fatty-acid metabolism. These findings were consistent with impaired glucose and lipid metabolism in the diabetic liver. Fewer pathways met the enrichment criteria in the model-versus-RS comparison, suggesting that raw RS affected a more limited set of metabolic and signaling processes. By contrast, a larger set of pathways was enriched in the model-versus-SF-RS comparison. These pathways included PI3K–Akt, AMPK, PPAR, FoxO, and mTOR signaling pathways. Thus, SF-RS treatment was associated with broader changes in proteins involved in insulin signaling, energy sensing, glucose regulation, and lipid metabolism. However, pathway enrichment indicates that altered proteins were overrepresented in these pathways and does not, by itself, establish pathway activation or inhibition.

GSEA was conducted for the model-versus-SF-RS comparison to assess coordinated changes across the full ranked protein list (Figure 6J–M). PI3K–Akt signaling showed significant positive enrichment, indicating that proteins assigned to this pathway were concentrated toward the SF-RS end of the ranked list. MAPK and FoxO signaling showed similar coordinated enrichment patterns, while PPAR signaling was also significantly enriched. These results suggest that SF-RS affected groups of proteins involved in insulin signaling, cellular stress responses, glucose metabolism, and lipid regulation. The GSEA findings were broadly consistent with the KEGG enrichment results.

Overall, diabetes induction produced extensive changes in the hepatic proteome, and both RS preparations shifted part of this altered protein pattern. SF-RS was associated with a broader movement toward the control proteomic state and affected more pathways related to insulin signaling and metabolic regulation than raw RS. These findings provide proteomic support for processing-dependent differences in the metabolic effects of RS, particularly in pathways involving PI3K–Akt, AMPK, PPAR, FoxO, and mTOR signaling.

### 3.6. Metabolomic Profiling Shows Distinct Metabolic Responses to RS and SF-RS in T2DM Mice

#### 3.6.1. Analytical Reproducibility and Stability of the LC-MS Metabolomic Dataset

To comprehensively characterize the metabolic alterations associated with RS and SF-RS intervention in diabetic mice, untargeted metabolomic profiling was performed using LC-MS/MS platforms under both positive and negative electrospray ionization modes. Prior to multivariate statistical analysis, pooled quality control (QC) samples were systematically evaluated to assess the stability and reproducibility of the analytical platform (Table S16). The QC evaluation demonstrated excellent instrumental performance throughout the metabolomic acquisition process. In positive ion mode, the RSD values of peak areas ranged from 1.58% to 6.08%, while retention time variations remained within 0.13–1.60%. Similarly, in negative ion mode, peak area RSD values ranged from 1.69% to 3.55%, accompanied by minimal retention time fluctuations (0.064–0.21%). These results indicate that the LC-MS/MS system maintained stable ionization efficiency, chromatographic separation, and mass spectrometric detection during the entire analytical sequence.

The low variation among QC injections confirmed that observed metabolic differences among experimental groups were primarily derived from biological perturbations rather than technical noise. Therefore, the acquired metabolomic dataset possessed sufficient reliability for subsequent unsupervised and supervised statistical analyses.

#### 3.6.2. PCA Revealed Global Metabolic Separation Between Diabetic Model Mice and RS/SF-RS-Treated Mice

**Figure 7:**
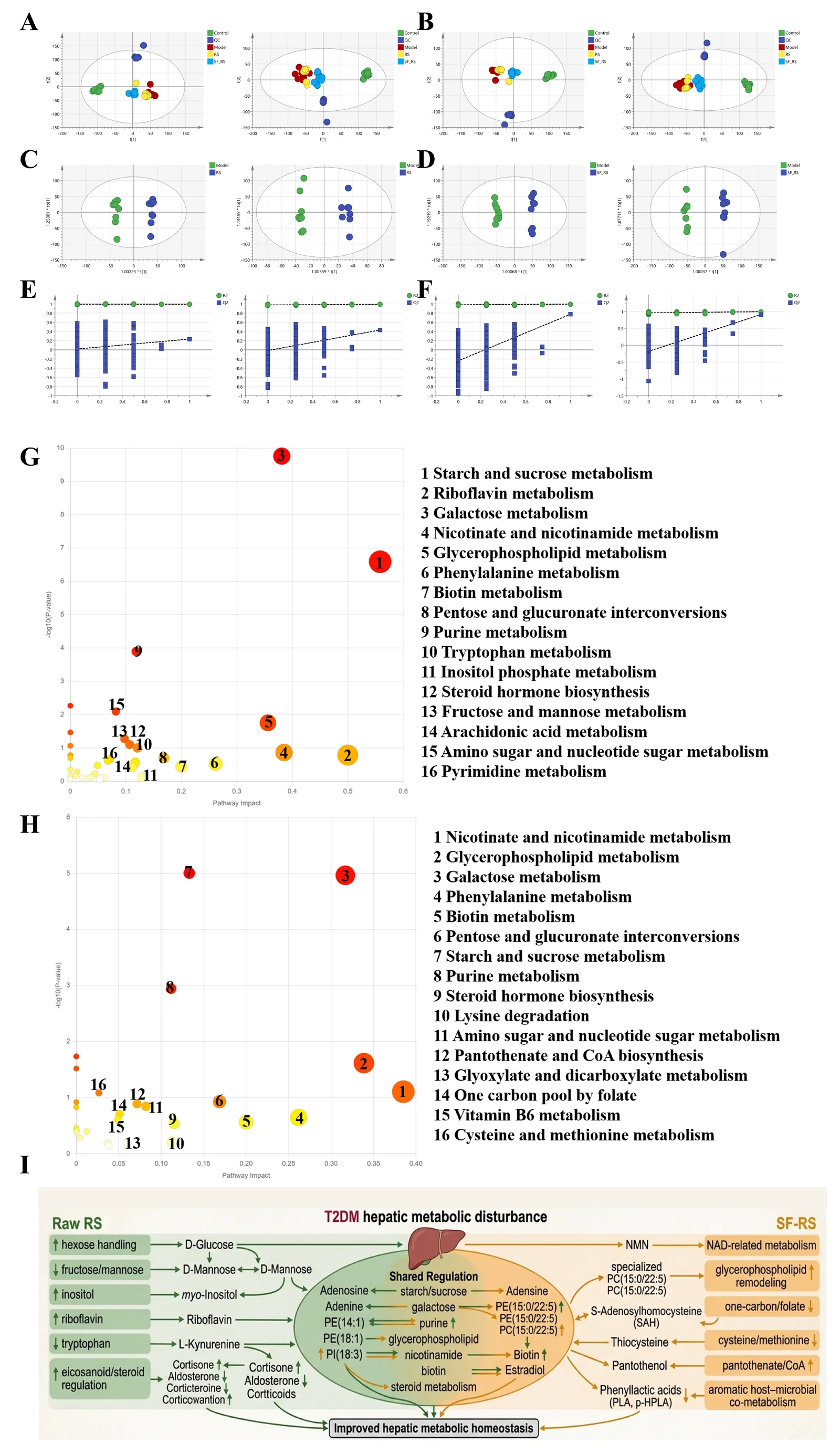
Hepatic metabolomic profiling and pathway-level metabolic regulation by raw and stir-fried *Raphani Semen* in T2DM mice (n = 8). (**A**) PCA score plots of hepatic metabolomic profiles under different acquisition modes. (**B**) PLS−DA score plots of hepatic metabolomic profiles under different acquisition modes. (**C,D**) OPLS−DA score plots under different acquisition modes. (**E**,**F**) Permutation tests of the OPLS-DA models. (**G**,**H**) Metabolic pathway enrichment and topology analysis of differential hepatic metabolites. (**I**) Integrated schematic representation of the hepatic metabolic regulatory networks associated with RS and SF-RS intervention in T2DM mice.

PCA was initially applied to evaluate overall metabolic distribution patterns and sample clustering. In both positive and negative ion modes, QC samples were tightly clustered, demonstrating strong analytical repeatability and confirming the robustness of the dataset. The PCA score plots showed clear separation between normal control animals and diabetic model animals, indicating that diabetes induction caused extensive remodeling of endogenous metabolic profiles (as shown in Figure 7A). Compared with the Model group, both RS and SF-RS treatment groups exhibited obvious shifts toward the metabolic characteristics of the Control group, suggesting that RS-derived interventions partially restored diabetes-associated metabolic disturbances. Notably, RS samples remained relatively close to the Model group and showed considerable overlap with Model samples, particularly in pairwise PCA analyses. In contrast, SF-RS samples exhibited a more pronounced displacement from the Model cluster and were positioned in an intermediate direction between the Model and Control metabolic phenotypes in both ionization modes. RS and SF-RS groups occupied distinct regions in PCA space rather than completely overlapping, suggesting that thermal processing altered the bioactive metabolic regulation pattern of *Raphani Semen*. This observation indicates that stir-frying did not simply enhance or weaken the metabolic effect of RS, but generated a remodeled intervention profile with unique biological characteristics.

#### 3.6.3. Supervised Multivariate Analysis Confirmed Distinct Metabolic Signatures Induced by RS and SF-RS

To further discriminate metabolic differences among groups, supervised PLS-DA and OPLS-DA models were constructed (as exhibited in Figure 7B–F). The PLS-DA score plots demonstrated clear discrimination between Model mice and RS/SF-RS-treated groups in both positive and negative ion modes (Figure 7B), indicating that intervention-associated metabolic alterations were sufficiently pronounced to distinguish treatment groups. The reliability of the supervised models was further validated by permutation testing. According to Table S17, the Model versus RS comparison showed satisfactory OPLS-DA performance, with positive ion mode exhibiting R^2^Y = 0.989 and Q^2^ = 0.432, while negative ion mode showed R^2^Y = 0.994 and Q^2^ = 0.235. The Model versus SF-RS comparison demonstrated even stronger model predictability, with positive ion mode showing R^2^Y = 0.996 and Q^2^ = 0.912, and negative ion mode showing R^2^Y = 0.995 and Q^2^ = 0.775. The high explanatory capacity and positive predictive ability of these models indicate that both RS and SF-RS markedly remodeled the metabolic phenotype of diabetic mice. However, the stronger separation and higher Q^2^ values observed in SF-RS-related models suggest that stir-frying generated a more pronounced metabolic regulatory effect compared with raw RS.

#### 3.6.4. RS and SF-RS Share a Core Metabolic Normalization Signature but Also Exhibit Extensive Processing-Dependent Divergence

A total of 194 differential metabolite annotations were compiled according to their directional changes among the Model, RS, and SF-RS groups (as listed in Table S18). Of these annotations, 122 were altered by both RS and SF-RS, whereas 46 were detected only in the RS comparison and 26 only in the SF-RS comparison. Among the 122 shared annotations, 97 changed in the opposite direction to the diabetes-associated alteration after both treatments, defining a common metabolic normalization signature. This shared signature encompassed multiple metabolite classes, including nicotinamide-related metabolites, purine and pyrimidine metabolites, vitamins and cofactors, steroid-related compounds, and numerous glycerophospholipid species. Representative metabolites included niacinamide, adenine, biotin, thiamine monophosphate, methionine sulfoxide, and multiple phosphatidylinositol, phosphatidylcholine, and phosphatidylethanolamine annotations. The common response, however, did not imply metabolic equivalence between the two treatments. Twenty-three shared annotations were shifted toward the control-associated direction by RS but changed further in the model-associated direction after SF-RS treatment. These annotations included pregnanediol-3-glucuronide, several prostaglandin- and leukotriene-related candidates, 5-methoxytryptamine, N-methylserotonin, phenylpyruvate-related candidates, and a series of highly unsaturated phosphatidylcholine species. Conversely, xanthine and 4-hydroxy-2-oxoglutaric acid showed differential responses that were more consistent with normalization by SF-RS than by RS. These findings demonstrate that stir-frying did not merely increase the intensity of the raw-RS response but reorganized the metabolic targets affected by treatment.

Among the 46 annotations uniquely altered by RS, most changed in the opposite direction to the diabetes-associated disturbance. A prominent feature was the regulation of a hexose-related LC–MS signal annotated with several isomeric candidates, including D-glucose, α-D-glucose, D-fructose, D-mannose, myo-inositol, and scyllo-inositol. This signal was increased in the Model group and decreased following RS treatment, whereas no significant change was detected after SF-RS treatment. RS-specific responses also involved riboflavin, L-carnitine, L-kynurenine, CDP-glucose, dIMP, dTDP-D-glucose, dTDP-D-galactose, selected phosphatidylcholine and phosphatidylethanolamine species, and steroid-related metabolites. Aldosterone- and cortisone-related annotations were elevated in the Model group and decreased selectively following RS administration. These metabolite-level changes were consistent with pathway analysis, in which RS-associated metabolites mapped to starch and sucrose metabolism, galactose metabolism, fructose and mannose metabolism, inositol phosphate metabolism, riboflavin metabolism, tryptophan metabolism, purine metabolism, glycerophospholipid metabolism, steroid hormone biosynthesis, and arachidonic acid metabolism. Together, these results indicate that raw RS preferentially affects hepatic substrate handling, hexose-related metabolism, selected vitamin-dependent reactions, and endocrine/lipid-signaling pathways.

The 26 annotations uniquely responsive to SF-RS displayed a highly coherent biochemical pattern. Several metabolites associated with sulfur amino-acid and one-carbon metabolism, including S-adenosylhomocysteine, thiocysteine, 3-mercaptolactic acid, and 10-formyldihydrofolate, were altered in the Model group and shifted in the opposite direction following SF-RS treatment, whereas raw RS produced no corresponding significant change. A second SF-RS-specific feature involved aromatic amino-acid-related metabolites. Phenyllactic acid-related candidates, including D-phenyllactic acid, L-3-phenyllactic acid, phenyllactic acid, and desaminotyrosine, were decreased in the Model group and increased after SF-RS treatment. In contrast, elevated homovanillic acid- and hydroxyphenyllactic acid-related signals were reduced by SF-RS. These coordinated alterations are compatible with extensive remodeling of aromatic amino-acid metabolism and are also consistent with modification of host–microbial co-metabolic processes, although the hepatic metabolomic dataset alone does not establish the microbial origin of individual metabolites. The pathway profile further distinguished SF-RS from raw RS. In addition to the pathways shared with RS, SF-RS-associated metabolites mapped to pantothenate and CoA biosynthesis, one-carbon pool by folate, vitamin B6 metabolism, cysteine and methionine metabolism, lysine degradation, and glyoxylate and dicarboxylate metabolism. This pattern indicates that stir-frying redirects the metabolic effects of RS toward a broader cofactor-dependent network involving one-carbon transfer, sulfur amino-acid turnover, CoA-associated metabolism, and redox-related biochemical processes.

Glycerophospholipid metabolism represented one of the most extensively affected metabolic systems in both treatment groups. Numerous phosphatidylcholine, phosphatidylethanolamine, phosphatidylinositol, lysophosphatidylcholine, and lysophosphatidic acid annotations were altered in the Model group and subsequently modulated by RS and/or SF-RS. Several phosphatidylinositol and phosphatidylcholine candidates that were decreased in the Model group increased after both treatments, whereas multiple phosphatidylethanolamine and lysophosphatidic acid candidates showing model-associated increases were reduced by both interventions. However, a distinct subset of highly unsaturated phosphatidylcholine species showed divergent treatment responses, with RS shifting these signals toward the Control-associated direction whereas SF-RS induced further changes in the Model-associated direction. Thus, both preparations targeted glycerophospholipid metabolism, but the individual lipid-species responses demonstrate substantial processing-dependent membrane-lipid remodeling. This divergence further supports the concept that stir-frying modifies the metabolic mode of action of RS rather than simply strengthening a quantitatively identical response.

Purine metabolism was consistently implicated by both the differential-metabolite dataset and pathway analysis. Adenine, adenosine-related metabolites, ADP, dGDP, dIMP, xanthine, deoxyadenosine, and several methylguanine-related annotations showed diabetes-associated perturbations and treatment-dependent regulation. Although many of these changes were shared by RS and SF-RS, the response of xanthine differed substantially between treatments, again indicating that the two interventions affect different nodes within the same metabolic pathway. Nicotinate and nicotinamide metabolism also represented a common metabolic target. The Model group showed decreased niacinamide and increased nicotinic acid mononucleotide, whereas both interventions shifted these metabolites in the opposite direction. The relatively prominent position of nicotinate and nicotinamide metabolism in the SF-RS pathway profile suggests that cofactor-related metabolic regulation may become more important following stir-frying. Steroid-related metabolites displayed a similar combination of convergence and divergence. Dehydroepiandrosterone-, testosterone-, epitestosterone-, and androstanedione-related signals were reduced in the Model group and increased by both treatments. In contrast, estradiol-related metabolites were selectively reduced by SF-RS, whereas aldosterone- and cortisone-related metabolites were selectively reduced by RS. These findings indicate that both preparations modulate steroid-associated metabolism while targeting partially distinct branches of the steroid metabolic network.

#### 3.6.5. Pathway-Level Integration Reveals a Shared Carbohydrate-Lipid Core but Distinct Metabolic Routes for RS and SF-RS

Pathway enrichment analysis identified a common metabolic core affected by both treatments (as shown in Figure 7G,H), including starch and sucrose metabolism, galactose metabolism, nicotinate and nicotinamide metabolism, glycerophospholipid metabolism, phenylalanine metabolism, biotin metabolism, pentose and glucuronate interconversions, purine metabolism, steroid hormone biosynthesis, and amino sugar and nucleotide sugar metabolism. Within the RS profile, galactose metabolism and starch and sucrose metabolism were among the most prominent pathways, accompanied by fructose and mannose metabolism, inositol phosphate metabolism, riboflavin metabolism, tryptophan metabolism, pyrimidine metabolism, and arachidonic acid metabolism. This pathway architecture was consistent with the RS-specific regulation of hepatic hexose-related signals, L-kynurenine, riboflavin, L-carnitine, steroid-related metabolites, and eicosanoid-related candidates. In contrast, SF-RS retained the major carbohydrate, purine, and glycerophospholipid components while additionally engaging one-carbon pool by folate, cysteine and methionine metabolism, pantothenate and CoA biosynthesis, vitamin B6 metabolism, lysine degradation, and glyoxylate and dicarboxylate metabolism. The enrichment pattern was supported by the SF-RS-specific regulation of S-adenosylhomocysteine, 10-formyldihydrofolate, thiocysteine, 3-mercaptolactic acid, and aromatic amino-acid-related metabolites.

Taken together, the metabolomic data indicate that RS and SF-RS converge on a common metabolic framework involving carbohydrate utilization, nucleotide turnover, glycerophospholipid homeostasis, and cofactor metabolism (as demonstrated in Figure 7I). However, raw RS exhibits a comparatively substrate-oriented metabolic response involving hexose, inositol, tryptophan, riboflavin, and selected steroid/eicosanoid pathways, whereas SF-RS produces a more extensive global reprogramming characterized by enhanced involvement of nicotinamide-related metabolism, glycerophospholipid remodeling, folate-dependent one-carbon transfer, sulfur amino-acid metabolism, CoA-associated metabolism, and aromatic host–microbial co-metabolism. These findings establish a processing-dependent metabolic signature that distinguishes stir-fried RS from its raw counterpart.

### 3.7. RS and SF-RS Are Associated with Distinct Gut Microbiota Profiles at the Study Endpoint in T2DM Mice

#### 3.7.1. ASV-Level Alpha- and Beta-Diversity Analysis

**Figure 8:**
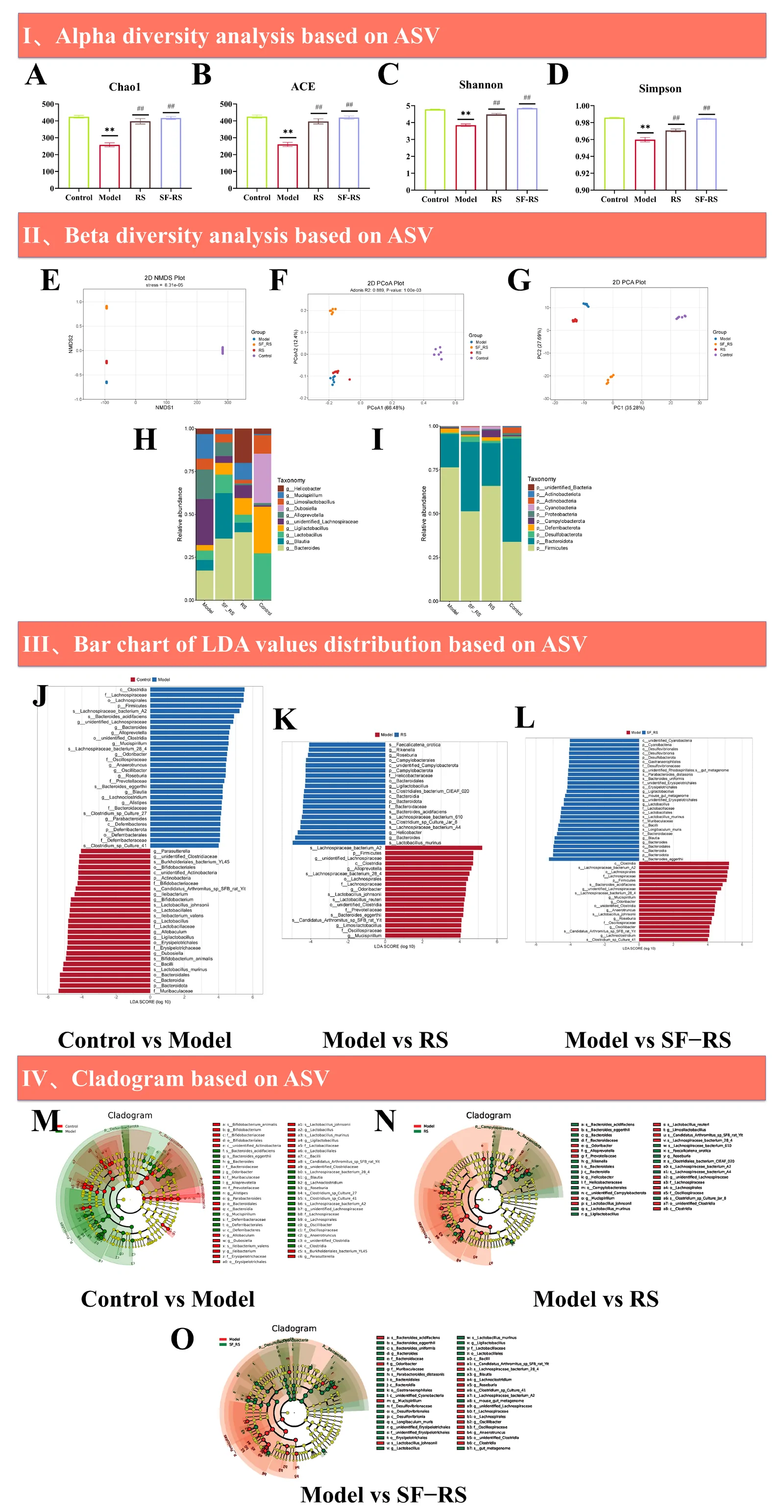
Stir-fried *Raphani Semen* produces broader gut microbiota remodeling than raw *Raphani Semen* in T2DM mice (n = 6). (**A**–**D**) Alpha diversity indices of the gut bacterial community. (**E**) NMDS based on Bray–Curtis dissimilarity. (**F**) PCoA based on unweighted UniFrac distance. (**G**) PCA of centered log-ratio-transformed microbial profiles. (**H**–**I**) Genus-level and Phylum-level composition of the gut microbiota. (**J**–**L**) LEfSe-derived LDA score plots. (**M**–**O**) Corresponding LEfSe cladograms for the control versus model, model versus RS, and model versus SF-RS comparisons. Data are presented as the mean ± SD, ** *p* < 0.01 versus the control group; ## *p* < 0.01 versus the model group; ns, not significant.

To determine whether RS and SF-RS modulated T2DM-associated gut microbial dysbiosis, fecal bacterial communities were characterized by 16 S rRNA gene sequencing. Compared with the control group, the model group exhibited markedly lower Chao1 and ACE richness estimates, together with reduced Shannon and Simpson diversity indices (*p* < 0.01, as shown in Figure 8A–D), indicating a pronounced loss of microbial richness and diversity following T2DM induction. Both RS and SF-RS significantly increased all four α-diversity indices relative to the model group (*p* < 0.01, as shown in Figure 8A–D). The values observed in the SF-RS group were numerically closest to those of the control group, whereas the RS group showed an intermediate response. β-diversity analyses further demonstrated marked group-dependent differences in overall microbial community structure. Non-metric multidimensional scaling (NMDS) based on Bray–Curtis dissimilarity clearly separated the four groups, with a two-dimensional stress value of 8.31 × 10^−5^ (as exhibited in Figure 8E).

Principal coordinate analysis (PCoA) based on the selected distance metric showed a similar pattern, with the first two coordinates explaining 66.48% and 12.40% of the total variation, respectively (Figure 8F). A global PERMANOVA confirmed significant differences in microbial composition among the groups (R^2^ = 0.889, *p* = 0.001). PCA of the centered abundance profiles also separated the four groups, with PC1 and PC2 accounting for 35.28% and 27.69% of the total variance, respectively (Figure 8G). The RS- and SF-RS-treated samples formed clusters that were distinct from both the model and control groups. This pattern suggests that the two treatments did not simply restore the microbiota to its control state but instead produced treatment-specific changes in community structure. Overall, RS and SF-RS attenuated the reduction in microbial richness and diversity associated with the diabetic phenotype and substantially altered bacterial community composition. The α-diversity measures in the SF-RS group were numerically closer to the control values than those in the RS group. The ordination plots also suggested a larger community-level shift after SF-RS treatment, although neither treatment resulted in complete overlap with the control group.

Taxonomic analysis further illustrated the different microbial responses to RS and SF-RS treatment (Figure 8H,I). At the genus level, the model group showed a clear change in the relative distribution of dominant taxa compared with the control group. Both treatments altered the abundance profiles of several common genera, including *Bacteroides*, *Blautia*, *Lactobacillus*, *Ligilactobacillus*, *Alloprevotella*, and unclassified members of the family *Lachnospiraceae* (Figure 8H). However, the patterns observed after RS and SF-RS treatment were not identical, indicating that stir-frying influenced the microbial response to RS. At the phylum level, *Firmicutes* and *Bacteroidota* were the dominant bacterial groups in all four groups. The model group showed a higher relative abundance of *Firmicutes* and a lower relative abundance of *Bacteroidota* than the control group. RS and SF-RS partly shifted these relative abundances toward the pattern observed in the control group, with a larger descriptive shift in the SF-RS group (Figure 8I). These taxonomic patterns were broadly consistent with the community-level analyses and suggest that stir-frying changed both the extent and the direction of RS-associated gut microbial remodeling.

#### 3.7.2. LEfSe-Based Identification of Differential Microbial Taxa

LEfSe analysis first defined the taxonomic signature associated with the diabetic state (as exhibited in Figure 8J–L). Relative to the Control group, Model mice were characterized by the overrepresentation of the *Firmicutes*–*Clostridia*–*Lachnospirales*/*Lachnospiraceae* axis, together with discriminant taxa such as *Mucispirillum*, *Odoribacter*, *Oscillibacter*, *Anaerotruncus*, *Alistipes*, *Parabacteroides*, *Bacteroides acidifaciens*, and Deferribacterota-related lineages. By contrast, the Control group was characterized by the *Actinobacteria*–*Bifidobacteriales*–*Bifidobacteriaceae*/*Bifidobacterium* branch, including *Bifidobacterium animalis*, as well as the *Bacilli*–*Lactobacillales*–*Lactobacillaceae*/*Lactobacillus* branch. Additional Control-associated biomarkers included *Lactobacillus johnsonii*, *Lactobacillus murinus*, *Ligilactobacillus*, *Muribaculaceae*, and several broad *Bacteroidota* lineages. These results established a diabetes-associated shift involving the loss of multiple Control-characteristic *lactobacilli*-, *bifidobacteria*-, and *Muribaculaceae*-related features.

Raw RS partially remodeled this diabetic taxonomic profile. Compared with the Model group, RS treatment was characterized by the enrichment of *Bacteroidota*, *Bacteroidia*, *Bacteroidales*, *Bacteroidaceae*, *Bacteroides*, *Ligilactobacillus*, and *L. murinus*. RS also selectively favored *Rikenella*, *Roseburia*, *Faecalicatena orotica*, and several *Lachnospiraceae*- and *Clostridiales*-related species. The recovery of *Bacteroidota*–*Bacteroidia*–*Bacteroidales*, *Ligilactobacillus*, and *L. murinus* represented the principal advantage of raw RS, because these features were also discriminant components of the Control microbiota. However, the RS-associated biomarker profile additionally contained *Campylobacterota*, *Campylobacterales*, *Helicobacteraceae*, and *Helicobacter*. Moreover, the Model-versus-RS comparison remained strongly defined by Model-associated *Firmicutes*, *Clostridia*, *Lachnospirales*, *Lachnospiraceae*, *Mucispirillum*, and *Odoribacter*. These findings indicate that raw RS induced a selective and taxon-specific correction rather than a comprehensive reorganization of the diabetic microbiota.

SF-RS generated a broader and phylogenetically more structured set of microbial biomarkers. Compared with untreated Model mice, SF-RS treatment was characterized by the coordinated enrichment of the complete *Bacilli*–*Lactobacillales*–*Lactobacillaceae*–*Lactobacillus* hierarchy, together with *Ligilactobacillus* and *L. murinus*. SF-RS also favored *Muribaculaceae*, *Bacteroidota*, *Bacteroidia*, *Bacteroidales*, *Bacteroidaceae*, *Bacteroides*, *Blautia*, *Bacteroides uniformis*, and *Parabacteroides distasonis*. Several of these lineages—including *Bacilli*, *Lactobacillales*, *Lactobacillaceae*, *Lactobacillus*, *Ligilactobacillus*, *L. murinus*, *Muribaculaceae*, and broad *Bacteroidota* taxa—were also characteristic of the Control group. The SF-RS intervention therefore reproduced a larger subset of Control-associated microbial features than raw RS.

Concurrently, the untreated Model group remained enriched relative to SF-RS in *Clostridia*, *Lachnospirales*, *Lachnospiraceae*, *Mucispirillum*, *Odoribacter*, *Anaerotruncus*, *Oscillibacter*, *Lachnoclostridium*, and several unidentified *Clostridia* and *Lachnospiraceae* taxa. This reciprocal pattern indicates that SF-RS not only recruited multiple Control-associated lineages but also reduced the dominance of a broad Model-associated taxonomic network. Notably, SF-RS also selected *Cyanobacteria*-related and *Desulfobacterota*–*Desulfovibrionia*-related lineages, as well as *Bacteroides eggerthii*. Therefore, the effect of SF-RS should be interpreted as the establishment of a distinct remodeled ecosystem rather than the uniform normalization of every bacterial taxon.

#### 3.7.3. Phylogenetic Distribution of Treatment-Associated Biomarkers

The LEfSe cladograms confirmed that the microbial differences were organized into phylogenetically coherent branches rather than being restricted to isolated ASVs (as shown in Figure 8M–O). In the Control-versus-Model comparison, Control-associated nodes were concentrated within the *Actinobacteria*–*Bifidobacteriales* and *Bacilli*–*Lactobacillales* branches, as well as *Muribaculaceae* and broad *Bacteroidota* lineages. Model-associated nodes were primarily distributed across the *Firmicutes*–*Clostridia*–*Lachnospirales*/*Lachnospiraceae* branch and the *Deferribacterota*-related lineage. This hierarchical organization reinforced the marked taxonomic restructuring caused by diabetes.

In the Model-versus-RS cladogram, RS-associated enrichment extended through the *Bacteroidota*–*Bacteroidia*–*Bacteroidales* axis and included *L. murinus* and *Ligilactobacillus*. These findings confirmed the ability of raw RS to recover selected bacterial branches that were also represented in the Control microbiota. However, a coherent *Campylobacterota*–*Campylobacterales*–*Helicobacteraceae*–*Helicobacter* branch was also associated with RS. Consequently, the phylogenetic response to raw RS combined the restoration of selected Control-associated lineages with the emergence of a distinct RS-specific taxonomic signature.

The Model-versus-SF-RS cladogram showed a more extensive multi-rank response. SF-RS-associated nodes covered both the *Bacteroidota*–*Bacteroidia*–*Bacteroidales* branch and the *Bacilli*–*Lactobacillales*–*Lactobacillaceae*–*Lactobacillus* branch, extending to *L. murinus* at the species level. *Muribaculaceae*, *B. uniformis*, *P. distasonis*, *Blautia*, and several *Erysipelotrichales*-related taxa were also incorporated into the SF-RS-associated network. In contrast, the untreated Model group retained a coherent *Firmicutes*–*Clostridia*–*Lachnospirales*–*Lachnospiraceae* branch and multiple *Mucispirillum*-, *Odoribacter*-, *Oscillibacter*-, and *Oscillospiraceae*-related nodes. The greater phylogenetic breadth of the SF-RS-associated clades, together with their stronger overlap with Control-associated bacterial branches, provides lineage-level evidence that stir-frying enhanced the microbiota-modulatory capacity of *Raphani Semen*.

#### 3.7.4. Integrated Comparison Between Raw and Stir-Fried *Raphani Semen*

Taken together, raw RS exhibited a clear but focused regulatory advantage. It substantially restored α-diversity and selectively expanded Bacteroidota-related bacteria, Ligilactobacillus, *L. murinus*, Rikenella, and Roseburia. Nevertheless, its β-diversity profile remained comparatively close to that of untreated Model mice, and its LEfSe signature contained a distinct Campylobacterota–Helicobacteraceae–Helicobacter component.

By comparison, SF-RS displayed a broader and more internally coherent regulatory profile. It restored all four α-diversity indices to values close to those of the Control group, induced a more pronounced compositional displacement from the untreated diabetic state, and recruited a larger set of Control-associated lineages. Particularly notable was the coordinated recovery of the *Bacilli*–*Lactobacillales*–*Lactobacillaceae*–*Lactobacillus* hierarchy, together with *Ligilactobacillus*, *L. murinus*, *Muribaculaceae*, *Bacteroidota*, *B. uniformis*, and *P. distasonis*. The absence of the RS-associated *Helicobacter*/*Campylobacterota* signature further distinguished the response to the processed preparation. These convergent ecological, compositional, and phylogenetic findings support the interpretation that stir-frying potentiated, rather than merely preserved, the gut microbiota-regulatory activity of *Raphani Semen* in T2DM mice.

### 3.8. Multi-Omics Correlation Analysis Identifies Associations Between Gut Microbial Remodeling and Hepatic Molecular Responses to SF-RS

To delineate the microbiota–liver molecular coupling associated with SF-RS intervention, phylum-level bacterial profiles were integrated with the hepatic proteomic and metabolomic matrices using Mantel tests (Figure S5). Six phyla, including unidentified *Bacteria*, *Actinobacteriota*, *Actinobacteria*, *Deferribacterota*, *Bacteroidota*, and *Firmicutes*, were significantly associated with both host omics layers (*p* < 0.05), indicating coordinated variation among the intestinal microbial community, hepatic protein expression, and hepatic metabolic phenotype. The proteomic profile showed particularly strong associations with unidentified *Bacteria*, *Actinobacteriota*, *Actinobacteria*, and *Bacteroidota* (Mantel r ≥ 0.8, *p* < 0.01). *Deferribacterota* was also strongly associated with the hepatic proteome (r = 0.6–0.8, *p* < 0.01), whereas *Firmicutes* exhibited an association of comparable magnitude with a lower level of statistical significance (r = 0.6–0.8, *p* = 0.01–0.05). In addition, *Campylobacterota* was selectively associated with the proteomic profile (r = 0.2–0.4, *p* = 0.01–0.05).

For the hepatic metabolome, strong associations were detected for *Deferribacterota*, *Bacteroidota*, and *Firmicutes* (r = 0.6–0.8, *p* < 0.01), while *Actinobacteria* showed a similarly strong Mantel correlation with a *p* value between 0.01 and 0.05. Unidentified *Bacteria* and *Actinobacteriota* displayed moderate but significant associations with the metabolomic profile (r = 0.4–0.6, *p* = 0.01–0.05). By contrast, *Cyanobacteria*, *Proteobacteria*, and *Desulfobacterota* were not significantly related to either hepatic omics matrix.

The phylum-level Spearman correlation structure further revealed two opposing microbial modules. *Actinobacteria* was strongly positively correlated with *Bacteroidota* but negatively correlated with *Deferribacterota* and *Firmicutes*. Conversely, *Deferribacterota* was positively correlated with *Firmicutes* and inversely correlated with *Bacteroidota*. A pronounced negative relationship was also observed between *Bacteroidota* and *Firmicutes* (r ≈ −1.0, *p* < 0.001). Consistent with the SF-RS-induced rebalancing of *Bacteroidota* and *Firmicutes* described above, the simultaneous associations of these phyla with both hepatic omics layers identify the *Bacteroidota*–*Firmicutes* axis as a major interface linking intestinal microbial remodeling to hepatic molecular responses. These associations indicate coordinated changes in the intestinal microbial community and hepatic molecular phenotype after SF-RS treatment. However, because the analysis is based on compositional microbiome data and cross-omics correlations, it does not establish causal gut-liver signaling, its directionality, or a mediating role of the microbiota.

To further investigate the coordinated alterations among hepatic metabolites, proteins, and gut microbiota, cross-omics correlation analyses were performed (Figure S6). The metabolite–protein correlation analysis revealed two distinct and largely reciprocal association patterns (Figure S6A). Multiple phosphatidylinositol (PI) and phosphatidylcholine (PC) species, together with steroid-related metabolites including androstenedione, epitestosterone, dehydroepiandrosterone, and testosterone, showed strong positive correlations with proteins such as P12710 and P70236, but negative correlations with P30285 and P35821, with several correlation coefficients reaching approximately ±0.8–1.0. In contrast, LysoPA species, PE(14:1(9Z)/14:1(9Z)), 6-ketoprostaglandin E1, 11-dehydro-thromboxane B2, D-erythrose 4-phosphate, and uridine diphosphate sugars exhibited an opposite correlation pattern.

Consistent relationships were observed between hepatic metabolites and gut microbiota (Figure S6B). The PI/PC- and steroid-associated metabolite cluster was strongly negatively correlated with *Deferribacterota* and *Firmicutes* (r values approaching −0.9), while showing strong positive correlations with *Actinobacteria* and *Bacteroidota* (r values approaching 0.9). Conversely, LysoPA, PE, 6-ketoprostaglandin E1, 11-dehydro-thromboxane B2, and D-erythrose 4-phosphate displayed largely inverse associations with these bacterial phyla. Protein–microbiota correlations further supported this pattern (Figure S6C), notably, P12710 and P70236 were positively correlated with *Actinobacteria* and *Bacteroidota* (r = 0.9) and negatively correlated with *Deferribacterota* and *Firmicutes* (r= −0.9), whereas P30285 and P35821 showed the opposite relationships. Integration of the three omics datasets generated a densely interconnected network involving microbial phyla, hepatic proteins, and metabolites related to phospholipid, steroid, eicosanoid, and carbohydrate metabolism (Figure S6D). Collectively, these cross-omics associations indicate coordinated changes in gut microbial composition, hepatic metabolic profiles, and protein expression following stir-fried *Raphani Semen* intervention in T2DM mice.

### 3.9. Molecular Validation Confirms Stronger Regulation of Hepatic Lipid Metabolism and Insulin-Related Signaling by SF-RS

**Figure 9:**
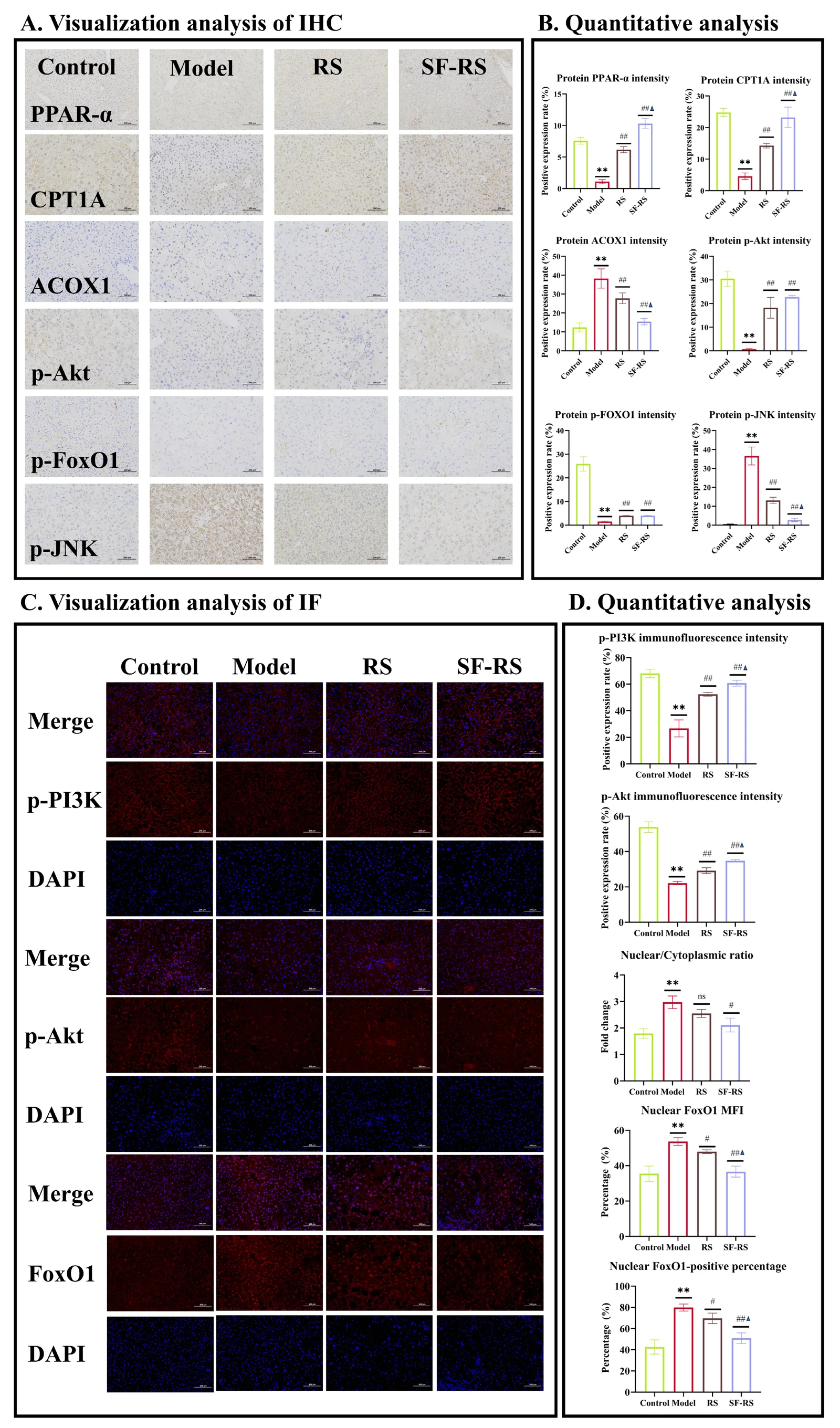
Effects of raw and stir-fried *Raphani Semen* on hepatic PPARα-associated fatty acid metabolism and PI3K/Akt/FoxO1/JNK signaling in diabetic mice. (**A**) Representative immunohistochemical images of PPAR-α, CPT1A, ACOX1, p-Akt, p-FoxO1, and p-JNK in liver sections. (**B**) Quantitative analysis of the positive expression rates of immunohistochemical images. (**C**) Representative immunofluorescence images of p-PI3K, p-Akt, and FoxO1 in liver sections. (**D**) Quantification of p-PI3K and p-Akt immunofluorescence, the nuclear-to-cytoplasmic FoxO1 fluorescence ratio, nuclear FoxO1 mean fluorescence intensity (MFI), and the percentage of FoxO1-positive nuclei. Data are presented as mean ± SD. ** *p* < 0.01 versus the Control group; # *p* < 0.05 and ## *p* < 0.01 versus the Model group; ns, not significant. The triangle indicates that, compared with the RS group, the *p* value of the SF-RS group is less than 0.05.

Proteomic enrichment analysis implicated PPAR, PI3K–Akt, FoxO, and MAPK signaling in the hepatic response to SF-RS. Key proteins representing these pathways were therefore examined by immunohistochemistry (IHC) and immunofluorescence (IF) to determine whether the pathway-level alterations were accompanied by corresponding changes at the protein and subcellular levels (Figure 9A–D).

IHC analysis first showed substantial disruption of proteins involved in hepatic fatty-acid metabolism (Figure 9A,B). Compared with the control group, diabetic mice exhibited markedly lower PPAR-α and CPT1A staining and substantially higher ACOX1 expression (*p* < 0.01), consistent with an altered balance between mitochondrial and peroxisomal fatty-acid oxidation. Both RS and SF-RS shifted these proteins toward the control-associated pattern. However, the effect was more pronounced after stir-frying: PPAR-α and CPT1A expression was significantly higher in the SF-RS group than in the RS group, whereas ACOX1 expression was significantly lower (*p* < 0.05 for the direct RS–SF-RS comparisons). These protein-level differences were consistent with the PPAR-related changes identified by proteomic analysis and indicate a stronger normalization of hepatic fatty-acid metabolic regulation following SF-RS treatment.

The insulin- and stress-signaling proteins examined by IHC were also markedly altered in diabetic liver. The model group showed pronounced reductions in p-Akt and p-FoxO1, together with a substantial increase in p-JNK, relative to the control group (*p* < 0.01; Figure 9A,B). Both preparations significantly reversed these diabetes-associated changes relative to the model group. Direct comparison between the two treatments showed significantly lower p-JNK staining after SF-RS administration than after RS treatment (*p* < 0.05). In contrast, although p-Akt and p-FoxO1 values were shifted further toward the control-associated levels in the SF-RS group, the corresponding RS–SF-RS differences were not statistically significant in the IHC analysis.

IF analysis provided additional evidence for regulation of the upstream PI3K/Akt axis (Figure 9C,D). Diabetes markedly decreased hepatic p-PI3K and p-Akt fluorescence intensity compared with the control condition (*p* < 0.01). RS treatment partially restored both signals, whereas SF-RS produced a greater recovery. Quantitative analysis confirmed significantly higher p-PI3K and p-Akt fluorescence intensities in the SF-RS group than in the RS group (*p* < 0.05), providing direct evidence that stir-frying strengthened the effect of RS on hepatic PI3K/Akt signaling.

FoxO1 subcellular distribution was subsequently examined because its nuclear localization provides a functional readout of insulin-related signaling. Diabetic mice displayed a marked increase in the FoxO1 nuclear-to-cytoplasmic ratio, nuclear FoxO1 mean fluorescence intensity (MFI), and the proportion of FoxO1-positive nuclei compared with control mice (*p* < 0.01, Figure 9C,D). RS reduced nuclear FoxO1 accumulation, although its effect on the nuclear-to-cytoplasmic ratio did not reach statistical significance relative to the model group. SF-RS significantly reduced the nuclear-to-cytoplasmic ratio and produced more pronounced decreases in both nuclear FoxO1 MFI and the percentage of FoxO1-positive nuclei. Direct RS–SF-RS comparisons further demonstrated significantly lower nuclear FoxO1 MFI and FoxO1-positive nuclear percentage following SF-RS treatment (*p* < 0.05), whereas the difference in the nuclear-to-cytoplasmic ratio between the two preparations was not significant.

Taken together, the targeted protein analyses were consistent with the pathway patterns identified by hepatic proteomics. Relative to raw RS, SF-RS exerted stronger effects on PPAR-α/CPT1A/ACOX1-associated fatty-acid metabolism, enhanced p-PI3K and p-Akt signaling, more effectively suppressed JNK phosphorylation, and reduced nuclear accumulation of FoxO1. Notably, the processing-related advantage was supported by direct RS–SF-RS comparisons for several key molecular endpoints, whereas p-Akt and p-FoxO1 measured by IHC and the FoxO1 nuclear-to-cytoplasmic ratio did not differ significantly between the two treatments. These results provide molecular evidence that stir-frying selectively strengthens the regulation of hepatic lipid metabolism and insulin-responsive signaling by *Raphani Semen*.

## 4. Discussion

The present study was designed to determine whether a representative stir-frying condition alters the chemical exposure profile and metabolic effects of RS, and to explore the biological processes associated with any processing-dependent differences. To minimize the possibility that the observed differences between RS and SF-RS reflected a single-batch processing artifact, the stir-frying procedure was independently repeated three times under the same predefined conditions. The resulting SF-RS batches showed comparable gross feature, fingerprint similarity and marker-compound contents, supporting the batch-to-batch reproducibility of the processing procedure within the conditions examined. These data strengthen the interpretation that the chemical changes associated with stir-frying were reproducible rather than unique to a single preparation. Nevertheless, the present three-batch assessment represents an initial reproducibility evaluation rather than a comprehensive process-validation study. Larger-scale validation across additional raw-material lots, operators, and processing runs will be necessary to establish the robustness and generalizability of the procedure. A more detailed analysis of the processing reproducibility will be reported separately.

It should also be noted that although processing reproducibility was assessed using three independent SF-RS preparations, the downstream in vivo experiment was performed using one representative batch. Thus, the present reproducibility assessment supports consistency of the preparation procedure but does not constitute an independent biological replication of the treatment effects across multiple SF-RS batches. Across the matched doses examined, the most intuitive difference between RS and SF-RS was observed. SF-RS produced lower terminal fasting glucose, circulating insulin, and HOMA-IR than the corresponding RS treatment, while an additional difference in glucose tolerance was evident at the low dose. In contrast, between-preparation differences in circulating lipid indices and liver-associated biochemical parameters were less uniform and were dependent on the specific endpoint and dose. These findings therefore support a processing-associated difference in metabolic phenotype, with the most reproducible distinction occurring in glucose- and insulin-related outcomes, rather than a uniform enhancement of all biological effects after stir-frying.

The chemical profiling data provide compositional context for, but do not establish the chemical basis of, the processing-associated pharmacological differences. Targeted HPLC-UV analysis provided an absolute quantitative reference for sinapine, with a content of 5.1–5.5 mg/g across three independently prepared SF-RS batches, the batch used for the pharmacological experiments contained 5.5 mg/g. In contrast, the remaining chemical comparisons were derived from non-targeted LC-MS peak-area responses without compound-specific calibration. Accordingly, the higher or lower signals observed for glucosinolate-, phenolamide-, flavonoid-, and other annotated features [13,32] should be interpreted as relative processing-associated analytical patterns rather than absolute concentration changes. The serum LC-MS data likewise identify differences in the presence and relative responses of drug-related features but do not establish quantitative systemic exposure or bioavailability. Therefore, although several glucosinolate-related features showed concordant relative signal patterns in SF-RS and serum, the present data do not demonstrate that these constituents quantitatively account for the stronger pharmacological responses observed with SF-RS. Targeted quantification of these candidate compounds in the administered preparation and pharmacokinetic measurements will be required to establish compound-specific exposure–effect relationships.

Integrated omics analysis and targeted tissue validation further linked the processing-dependent phenotype to insulin-responsive and lipid-metabolic pathways. In diabetic mice, impaired PI3K–Akt signaling was accompanied by reduced FoxO1 phosphorylation and increased nuclear accumulation of FoxO1, a pattern consistent with weakened insulin-mediated control of hepatic glucose metabolism. SF-RS produced a stronger recovery of p-PI3K and p-Akt than raw RS in the immunofluorescence analysis and significantly reduced nuclear FoxO1 MFI and the proportion of FoxO1-positive nuclei. Although the RS–SF-RS difference in the FoxO1 nuclear-to-cytoplasmic ratio and in p-FoxO1 measured by immunohistochemistry did not reach statistical significance, the combined signaling and localization data support more effective regulation of the PI3K–Akt–FoxO1 axis after stir-frying. FoxO1 activity depends strongly on its intracellular localization rather than on total protein abundance alone, and hepatic FoxO1 is a major regulator of insulin signaling and glucose homeostasis [33,34]. Gluconeogenic flux was not measured directly, and the present findings therefore do not establish a causal reduction in hepatic glucose production. Nevertheless, the consistent reductions in fasting glucose, insulin, and HOMA-IR, together with the improvement in glucose tolerance and the greater recovery of hepatic PAS-positive material, provide convergent functional evidence that the stronger glucose-regulating effect of SF-RS is associated with improved hepatic insulin responsiveness and FoxO1-dependent metabolic control.

The lipid-related molecular findings complement this glycemic phenotype. SF-RS produced significantly greater recovery of PPAR-α and CPT1A and a stronger reduction in the abnormally elevated ACOX1 signal than raw RS. This pattern suggests improved coordination between mitochondrial fatty-acid utilization and peroxisomal oxidation rather than a uniform stimulation of lipid catabolism. The recovery of PPAR-α and CPT1A is consistent with improved mitochondrial fatty-acid import and oxidation, whereas the elevated ACOX1 observed in untreated diabetic mice may represent a compensatory increase in peroxisomal oxidation under conditions of impaired mitochondrial lipid handling. Because peroxisomal fatty-acid oxidation can generate H_2_O_2_, excessive reliance on this pathway may also contribute to oxidative stress. The ability of SF-RS to restore PPAR-α and CPT1A while reducing ACOX1 toward the control-associated state is therefore consistent with a more balanced hepatic lipid-metabolic response. This interpretation is supported by evidence that FoxO1 and PPAR-α participate in an interconnected transcriptional network regulating hepatic adaptation to metabolic stress [35]. At the physiological level, these molecular changes were accompanied by reductions in hepatic lipid accumulation and collagen deposition, although the direct SF-RS advantage over RS was confined to selected doses rather than being universal across all lipid-related endpoints.

Proteomic and metabolomic analyses further supported a processing-dependent reorganization of hepatic metabolism. SF-RS was associated with broader changes in PI3K–Akt, FoxO, PPAR, MAPK, and related metabolic pathways, together with alterations in nicotinamide metabolism, CoA biosynthesis, one-carbon metabolism, sulfur amino-acid metabolism, and glycerophospholipid remodeling. These findings indicate that stir-frying did not simply intensify an otherwise identical response to raw RS. Instead, the processed material produced a distinct metabolic regulatory pattern, while retaining a common core involving carbohydrate utilization, nucleotide turnover, lipid homeostasis, and cofactor metabolism. Such changes are compatible with improved insulin responsiveness, energy metabolism, and lipid handling, although the individual omics features should be interpreted as components of an integrated metabolic state rather than as independent causal drivers.

Gut microbial analysis revealed a similarly processing-dependent response. Both preparations alleviated the loss of microbial richness and diversity associated with the diabetic phenotype, but SF-RS was associated with a broader compositional shift and recruited a larger set of lineages also represented in the control microbiota, including *Lactobacillales*/*Lactobacillus*-related taxa, *Muribaculaceae*, *Bacteroidota*, *Bacteroides uniformis*, and *Parabacteroides distasonis*. The microbiota after SF-RS treatment should not, however, be interpreted as a simple return to the control state, because the processed preparation generated a distinct remodeled microbial ecosystem. Associations of *Bacteroidota*, *Firmicutes*, and other phyla with both hepatic proteomic and metabolomic profiles further indicate that intestinal ecological changes occurred in parallel with hepatic metabolic regulation. Diet–microbiome interactions are increasingly recognized as important modifiers of cardiometabolic responses to foods [36]. Nevertheless, these associations remain correlative; compositional 16S data and cross-omics correlations cannot establish that the microbial changes caused the improvement in glucose metabolism. The gut microbiota should therefore be viewed here as a component associated with the processing-dependent metabolic phenotype rather than as a proven causal mediator.

The present findings are consistent with previous reports showing that *Raphanus sativus*-derived preparations can influence glucose metabolism, hepatic lipid accumulation, insulin sensitivity, and gut microbial composition [14,15,37,38]. The current study extends these observations by indicating that the metabolic effects of *Raphani Semen* may vary according to its processing state. In particular, the more consistent glucose- and insulin-related responses observed with SF-RS suggest that stir-frying may modify the functional profile of RS in a manner that is more evident for glucose regulation than for lipid metabolism or liver-associated biochemical outcomes.

Several factors should be considered when interpreting these findings. The serum pharmacochemistry and efficacy experiments were conducted in different rodent species for practical reasons, rats provided sufficient serum for comprehensive LC–MS profiling, whereas C57BL/6 mice were used for the diabetic efficacy study. Therefore, the rat serum data identify candidate systemically exposed constituents but do not establish their quantitative exposure in the diabetic mice. In addition, most chemical results were based on relative LC–MS abundance, and absolute concentrations and individual constituent doses were not determined for all detected compounds. Thus, the present data support processing-associated differences in chemical exposure but do not establish a quantitative exposure–effect relationship for any single constituent.

The STZ-induced diabetic model also represents a severe chemically induced metabolic phenotype. The repeated STZ regimen was selected because it produced the diabetic state required for the intervention study under our experimental conditions, but repeated STZ exposure may involve extra-pancreatic effects, including renal injury [39,40,41]. Accordingly, safety considerations should also be taken into account when interpreting the metabolic effects observed in this study. The serum ALT, AST, and ALP results did not show a treatment-related elevation at the highest tested doses of RS or SF-RS, providing no biochemical evidence of high-dose hepatotoxicity under the present experimental conditions. To further assess renal safety, we additionally measured serum Cr, BUN, and CysC. The diabetic model group showed marked elevations in Cr and BUN relative to the control group, whereas CysC remained unchanged, indicating that the renal biochemical changes associated with this model were not uniform across the three markers. Importantly, neither RS nor SF-RS treatment caused further increases in Cr or BUN. Instead, both markers were significantly lower in all treatment groups than in the model group, while CysC remained unchanged. At the high dose in particular, neither RS-H nor SF-RS-H showed evidence of worsening renal function-related biochemical indices. Taken together with the hepatic enzyme data, these findings provide no biochemical evidence that the observed metabolic improvement was driven by nonspecific systemic toxic stress. Nevertheless, the renal safety conclusions should be interpreted cautiously. Although Cr, BUN, and CysC provide complementary information on renal function, urinary albumin-to-creatinine ratio was not assessed and renal tissues suitable for histopathological evaluation were not available. Therefore, subclinical, structural, transient, or earlier renal injury cannot be excluded. Moreover, because repeated STZ administration itself may contribute to renal injury in addition to the effects of diabetes, the present serum measurements cannot distinguish potential direct STZ toxicity from hyperglycemia-related renal alterations. Accordingly, the current findings support the absence of an overt treatment-related deterioration in renal biochemical indices at the study endpoint, rather than establishing complete renal safety.

Accordingly, the metabolic and organ-related findings should be interpreted within the context of this model. Similarly, ALT, AST, and ALP provide useful liver-associated biochemical information but do not constitute a comprehensive safety assessment, particularly at the highest dose. Further studies using complementary models, quantitative pharmacokinetics, systematic variation in processing conditions, and more comprehensive hepatic and renal safety evaluations will be important for defining the reproducibility, mechanism, and translational relevance of the observed effects.

Collectively, the present data indicate that raw and stir-fried *Raphani Semen* produced distinguishable chemical-exposure and metabolic-response profiles under the conditions examined. The most consistent differences involved glucose- and insulin-related outcomes and occurred alongside changes in hepatic insulin-responsive signaling, lipid-metabolic regulation, systemic metabolic pathways, and gut microbial composition. These findings support an association between stir-frying, altered chemical exposure, and metabolic phenotype, while providing a basis for further investigation of how controlled thermal processing may modify the metabolic properties of *Raphani Semen*.

## 5. Conclusions

This study shows that raw and stir-fried *Raphani Semen* exhibit distinct chemical profiles, serum-detectable constituent patterns, and metabolic-response profiles under the experimental conditions examined. The stronger glucose- and insulin-regulated efficacy observed with SF-RS occurred in parallel with changes in serum-detectable chemical features, hepatic PI3K–Akt/FoxO- and PPAR-related regulation, metabolic networks, and gut microbial composition. But the individual chemical constituents responsible for this effect remain to be determined by targeted quantitative and pharmacokinetic studies. The significance of these findings lies in identifying processing state as a potentially important source of variation in the efficacy of *Raphani Semen*. This provides a scientific basis for selecting and optimizing processing conditions in future studies and offers candidate chemical, hepatic, metabolic, and microbial features for subsequent mechanistic investigation.

## Data Availability

The data that support the findings of this study are available within the article and its Supplementary Materials.

## Supplementary Materials

The following supporting information can be downloaded at https://www.mdpi.com/article/10.3390/foods15203587/s1, Figure S1: Representative chromatograms of the raw and stir-fried RS samples detected under the 225 nm wavelength; Figure S2: Representative chromatograms of the raw and stir-fried RS samples detected under the 325 nm wavelength; Figure S3: The visualization analysis of body weight and food intake; Figure S4: Quality assessment of the LC-MS/MS-based proteomic dataset; Figure S5: Mantel test-based integration of gut microbial phyla with hepatic proteomic and metabolomic profiles in T2DM mice; Figure S6: Integrated correlation analysis of hepatic metabolomic, hepatic proteomic, and gut microbiota profiles in T2DM mice following stir-fried *Raphani Semen* intervention; Table S1: UPLC-HRMS parameters for blood-absorbed constituents; Table S2: The LC-MS-related parameters for liver metabolomics; Table S3: Detailed information of antibodies used for immunohistochemistry; Table S4: Detailed information of antibodies used for immunofluorescence staining; Table S5: Experimental design, sample size, statistical unit, randomization and blinding; Table S6: Macroscopic and organoleptic characteristics of raw and stir-fried RS prepared under different processing conditions; Table S7: Summary of the reproducibility assessment of three independent SF-RS stir-frying preparations; Table S8: The identified compounds in RS samples; Table S9: The identified compounds in SF-RS samples; Table S10: Putatively annotated prototype constituents detected in serum after administration of RS; Table S11: Putatively annotated prototype constituents detected in serum after administration of SF-RS; Table S12: Animal mortality, exclusions, and numbers included in the analyses; Table S13: Individual blood glucose measurements at two pre-intervention assessments; Table S14: Multiplicity-adjusted *p* values for pairwise comparisons among experimental groups across biochemical parameters; Table S15: Serum renal function-related biomarkers and multiplicity-adjusted *p* values; Table S16: Quality assessment of LC-MS/MS metabolomic data based on pooled QC samples; Table S17: Validation parameters of OPLS-DA models for discrimination among experimental groups; Table S18: Differential metabolites identified by LC-MS/MS metabolomic profiling among diabetic model, RS-treated and SF-RS-treated groups.

## Abbreviations

ACOX1	Acyl-CoA oxidase 1
ALT	Alanine aminotransferase
ALP	Alkaline phosphatase
ANOVA	One-way analysis of variance
AST	Aspartate aminotransferase
CPT1A	Carnitine palmitoyltransferase 1A
DIA-LC–MS/MS	Data-independent acquisition liquid chromatography–tandem mass spectrometry
ELISA	Enzyme-linked immunosorbent assay kits
FDR	False discovery rate
FoxO1	Forkhead Box O1
GSEA	Gene set enrichment analysis
HDL-C	High-density lipoprotein cholesterol
HMDB	Human Metabolome Database
HPLC	High-performance liquid chromatograph
HSHFD	High-sugar and high-fat diet
IAM	Iodoacetemide
IDF	International diabetes federation
IR	Insulin resistance
LC-MS	Liquid chromatography-mass spectrometry
LDA	Linear discriminant analysis
LDL-C	Low-density lipoprotein cholesterol
LEfSe	Linear discriminant analysis effect size
MFI	Mean fluorescence intensity
MSI	Metabolomics Standards Initiative
NMDS	Non-metric multidimensional scaling
OGTT	Oral glucose tolerance test
OPLS-DA	Orthogonal partial least-squares discriminant analysis
PC	Phosphatidylcholine
PCA	Principal component analysis
PCoA	Principal coordinates analysis
PI	Phosphatidylinositol
PPAR-α	Peroxisome proliferator-activated receptor alpha
QC	Quality control
RS	*Raphani Semen*
SD	Standard deviation
SF-RS	Stir-fried *Raphani Semen*
SPF	Specific pathogen-free
STZ	Streptozocin
TC	Total cholesterol
T2DM	Type II diabetes mellitus
TEAB	Triethylammonium bicarbonate buffer
TFA	Trifluoroacetic acid
TG	Triglyceride
TIC	Total-ion chromatograms
UPLC-HRMS	Ultra-performance liquid chromatography–high-resolution tandem mass spectrometry
VIP	Variable importance in projection
