# Supplementary material for: Stir-Frying Reshapes the Chemical Profile and Metabolic Effects of Raphani Semen in Diabetic Mice: Integrated Analysis of Hepatic Metabolism and Gut Microbiota
Source: Foods. 2026 Oct 9;15(20):3587. doi: 10.3390/foods15203587 (PMC13703343; doi:10.3390/foods15203587)
Supplement: Supplementary file 1 [file foods-15-03587-s001.zip › Supplementary materials.pdf]

Stir-frying reshapes the chemical profile and metabolic effects of *Raphani Semen* in diabetic mice: integrated analysis of hepatic metabolism and gut microbiota

Table S1: UPLC-HRMS parameters for blood-absorbed constituents

**LC type**	Vanquish Horizon System (Thermo Scientific, USA)
**Column**	Waters ACQUITY UPLC BEH C18 column (100 mm × 2.1 mm, 1.7 μm)
**Flow rate**	0.4 mL/min
**Column temperature**	40°C
**Phase A**	Water (containing 0.1% formic acid)
**Phase B**	Acetonitrile (containing 0.1% formic acid)
**Elution program**	0-0.5 min, 2% B; 0.5-3.5 min, 2%-25% B; 3.5-7.5 min, 25%-35% B; 7.5-11 min, 35%-50% B; 11-13 min, 50%-95% B; 13-14.4 min, 95% B; 14.4-14.5 min, 95%-2% B; 14.5-16 min, 2% B
**MS type**	Q-Exactive (Thermo, USA)
**Ion Source type**	ESI
**Positive Ion**	3500V
**Negative Ion**	-3000V
**Sheath Gas**	50 Arb
**Aux Gas**	13 Arb
**Ion Source Heating Temperature**	450°C
**MS1 Resolution**	70000
**Scan Range (m/z)**	70-1050
**Cyclic collision energy**	20-40-60 V
**MS^2 resolution**	17500

Table S2. The LC-MS-related parameters for liver metabolomics

**LC type**	Dionex Ultimate 3000 UHPLC system (USA)
**Column**	Waters ACQUITY UPLC HSS T3 column
**Flow rate**	0.3 mL/min
**Column temperature**	40°C
**Phase A**	Water (containing 0.1% formic acid)
**Phase B**	Acetonitrile (containing 0.1% formic acid)
**Elution program**	0-1 min, 5% B; 1-5 min, 5%-70% B; 5-12 min, 70%-100% B; 12-13 min, 100%-5% B; 13-14 min, 5% B
**Ion Source type**	H-ESI
**Positive Ion**	3200V
**Negative Ion**	-2800V
**Sheath Gas**	20 Arb
**Aux Gas**	6 Arb
**Ion Transfer Tube Temperature**	300°C
**Vaporizer temperature**	320°C
**Detector type**	Orbitrap
**Orbitrap Resolution**	60000
**Scan Range (m/z)**	100-1000
**HCD collision energy**	15%, 25%, 35%
**dd MS^2 resolution**	15000

Table S3. Detailed information of antibodies used for immunohistochemistry

Primary antibody	Species	Supplier	Catalog No.	Dilution
PPAR-α	rabbit	Servicebio	GB11163	1:500
ACOX1	rabbit	Proteintech	10957-1-AP	1:300
CPT1A	rabbit	Proteintech	15184-1-AP	1:1000
p-FoxO1	rabbit	Servicebio	GB113974	1:300
p-Akt	rabbit	Servicebio	GB150002	1:500
p-JNK	rabbit	Bioss antibodies	bsm-52462R	1:200

Table S4. Detailed information of antibodies used for immunofluorescence staining

Primary antibody	Species	Supplier	Catalog No.	Dilution
p-PI3K	rabbit	Servicebio	GB150210	1:500
p-Akt	rabbit	Servicebio	GB150002	1:500
FoxO1	rabbit	Servicebio	GB154935	1:10000

Table S5. Experimental design, sample size, statistical unit, randomization and blinding

Experiment	Initial n	Analyzed n	Experimental unit	Randomization	Blinding	Exclusions
Processing screening	1 run/condition	1	Independent processing run	N/A	Samples randomly coded for sensory assessment	None
Serum-entrance compound	5 rats/group	5	Individual rat	Random-number table	N/A	None
Mouse phenotype	8 mice/group	8	Individual mouse	Random-number table	N/A	None
Histology	6 mice/group	6	Individual mouse	N/A	N/A	None
Proteomics	3	3	Individual mouse	N/A	N/A	None
Metabolomics	8	8	Individual mouse	N/A	N/A	None
16S rRNA	6	6	Individual mouse	N/A	N/A	None
IHC/IF	3	3	Individual mouse	N/A	N/A	None

Table S6. Macroscopic and organoleptic characteristics of raw and stir-fried RS prepared under different processing conditions

Sample code	Surface color	Crispness	Roasted aroma	Burnt odour
Raw RS	0	0	0	0
RS-140-1	0	0	1	0
RS-140-3	1	0	1	0
RS-140-5	1	1	1	0
RS-140-7	2	1	2	0
RS-140-9	2	2	3	1
RS-160-1	2	1	2	0
RS-160-3	2	2	3	0
RS-160-5	2	2	3	1
RS-160-7	3	3	1	2
RS-160-9	3	3	1	2
RS-180-1	3	3	1	1
RS-180-3	4	3	0	3
RS-180-5	4	4	0	3
RS-180-7	4	4	0	3
RS-180-9	4	4	0	3

Notes: Values are presented as the median (interquartile range) of scores independently assigned by three trained assessors. Surface color was scored as 0, reddish-brown, corresponding to the raw material; 1, slightly darkened reddish-brown; 2, brown; 3, dark brown; and 4, brown-black or nearly black. Crispness was scored as 0, hard or non-crisp; 1, slightly crisp; 2, crisp; and 3, highly crisp. Roasted aroma and burnt odour were independently scored as 0, absent; 1, faint; 2, moderate; and 3, strong.

Table S7. Summary of the reproducibility assessment of three independent SF-RS stir-frying preparations

Parameter	Batch 1	Batch 2	Batch 3
**Surface color**	2	2	2
**Crispness**	2	2	2
**Roasted aroma**	3	3	3
**Burnt odour**	0	0	0
**Sinapine (mg/g)**	5.2	5.5	5.1
**Fingerprint similarity**	0.93	0.96	0.92

Table S12. Animal mortality, exclusions, and numbers included in the analyses

**Part I Modeling and screening stage**			
**Group**	**Mortality**	**Exclusion**	**Numbers included**
Control	0	0	8
Model	0	0	64
**Part II Grouping and intervention phase**			
**Group**	**Mortality**	**Exclusion**	**Numbers included**
Control	0	0	8
Model	0	0	8
Metformin	0	0	8
RS-H	0	0	8
RS-M	0	0	8
RS-L	0	0	8
SF-RS-H	0	0	8
SF-RS-M	0	0	8
SF-RS-L	0	0	8

Notes: During both the modeling stage and the later group intervention stage, we recorded the death and removal situations of the mice. Fortunately, during both stages, no deaths were observed in the mice, nor was any elimination process recorded. The detailed blood glucose data are shown in Table S13.

Table S13. Individual blood glucose measurements at two pre-intervention assessments

Group	FBG 1	FBG2	Group	FBG 1	FBG2	Group	FBG 1	FBG2
Control	7.9	8.2	Model	19	16	Metformin	16.4	15.2
	7.7	7.5		18.9	15.3		17	16.6
	7.3	7.6		18.4	21.6		18.2	15.3
	7	7.3		21.4	22.6		17.7	16.4
	7.2	7.3		17.4	15.6		15.3	13.4
	7.7	7.5		18.2	16.8		14.5	12.7
	8.2	7.9		15.8	13.4		18.9	16.4
	7.4	7.7		14.7	12.5		19.2	21.3
RS-H	17	15.9	RS-M	18.5	17	RS-L	20.7	18.5
	24.1	22.5		16.4	15.6		23.4	21.1
	18.6	16		23.2	20.1		15.1	13.2
	17.2	16.4		14.5	15		16.4	18.1
	18.8	16.7		16.1	14.6		14.5	12.8
	15.3	15.5		16.2	15		17.2	16
	13.6	15.4		15.8	16		15.4	13.6
	20.4	17.3		17.7	15.9		21.5	22.1
SF-RS-H	17.8	15.9	SF-RS-M	20.4	16.1	SF-RS-L	15.4	13.5
	16.5	17.3		16.3	16.9		16.7	15.6
	15.8	13.9		15.1	14.3		15.8	16.3
	23.5	22.1		18.8	17.6		16.9	17.5
	18.2	17.1		17.5	16.1		19	16.8
	15.4	16.8		14	15.1		24.1	21.6
	16.7	18.3		18.6	20.7		12.4	11.8
	14.6	15.5		15.6	13.9		14	13.6

Table S14. Multiplicity-adjusted P values for pairwise comparisons among experimental groups across biochemical parameters

Biochemical parameter	Comparison	Adjusted P value	Biochemical parameter	Comparison	Adjusted P value
Last Blood Glucose	Control vs Model	<0.001	Insulin levels	Control vs Model	<0.001
	Model vs Metformin	<0.001		Model vs Metformin	<0.001
	Model vs RS-H	<0.001		Model vs RS-H	<0.001
	Model vs RS-M	0.084		Model vs RS-M	<0.001
	Model vs RS-L	0.717		Model vs RS-L	<0.001
	Model vs SF-RS-H	<0.001		Model vs SF-RS-H	<0.001
	Model vs SF-RS-M	<0.001		Model vs SF-RS-M	<0.001
	Model vs SF-RS-L	<0.001		Model vs SF-RS-L	<0.001
	RS-H vs SF-RS-H	0.007		RS-H vs SF-RS-H	0.012
	RS-M vs SF-RS-M	<0.001		RS-M vs SF-RS-M	0.699
	RS-L vs SF-RS-L	<0.001		RS-L vs SF-RS-L	0.049
HOMA-IR	Control vs Model	<0.001	AUC	Control vs Model	<0.001
	Model vs Metformin	<0.001		Model vs Metformin	<0.001
	Model vs RS-H	<0.001		Model vs RS-H	<0.001
	Model vs RS-M	<0.001		Model vs RS-M	0.045
	Model vs RS-L	<0.001		Model vs RS-L	0.627
	Model vs SF-RS-H	<0.001		Model vs SF-RS-H	<0.001
	Model vs SF-RS-M	<0.001		Model vs SF-RS-M	0.002
	Model vs SF-RS-L	<0.001		Model vs SF-RS-L	0.009
	RS-H vs SF-RS-H	<0.001		RS-H vs SF-RS-H	0.723
	RS-M vs SF-RS-M	<0.001		RS-M vs SF-RS-M	0.151
	RS-L vs SF-RS-L	<0.001		RS-L vs SF-RS-L	0.010
TC	Control vs Model	<0.001	TG	Control vs Model	<0.001
	Model vs Metformin	<0.001		Model vs Metformin	<0.001
	Model vs RS-H	0.001		Model vs RS-H	<0.001
	Model vs RS-M	0.040		Model vs RS-M	<0.001
	Model vs RS-L	0.675		Model vs RS-L	0.010
	Model vs SF-RS-H	<0.001		Model vs SF-RS-H	<0.001
	Model vs SF-RS-M	<0.001		Model vs SF-RS-M	<0.001
	Model vs SF-RS-L	0.082		Model vs SF-RS-L	<0.001
	RS-H vs SF-RS-H	0.151		RS-H vs SF-RS-H	0.207
	RS-M vs SF-RS-M	0.227		RS-M vs SF-RS-M	0.591
	RS-L vs SF-RS-L	0.183		RS-L vs SF-RS-L	0.308
LDL-C	Control vs Model	<0.001	HDL-C	Control vs Model	0.949
	Model vs Metformin	<0.001		Model vs Metformin	0.260
	Model vs RS-H	<0.001		Model vs RS-H	0.556
	Model vs RS-M	<0.001		Model vs RS-M	0.684
	Model vs RS-L	0.010		Model vs RS-L	0.576

	**Model vs SF-RS-H**	<0.001		**Model vs SF-RS-H**	0.429
	**Model vs SF-RS-M**	<0.001		**Model vs SF-RS-M**	0.873
	**Model vs SF-RS-L**	<0.001		**Model vs SF-RS-L**	0.755
	**RS-H vs SF-RS-H**	0.251		**RS-H vs SF-RS-H**	0.838
	**RS-M vs SF-RS-M**	0.633		**RS-M vs SF-RS-M**	0.571
	**RS-L vs SF-RS-L**	0.254		**RS-L vs SF-RS-L**	0.804
**ALT**	**Control vs Model**	<0.001	**AST**	**Control vs Model**	<0.001
	**Model vs Metformin**	<0.001		**Model vs Metformin**	<0.001
	**Model vs RS-H**	0.020		**Model vs RS-H**	<0.001
	**Model vs RS-M**	0.045		**Model vs RS-M**	0.001
	**Model vs RS-L**	0.721		**Model vs RS-L**	0.002
	**Model vs SF-RS-H**	<0.001		**Model vs SF-RS-H**	<0.001
	**Model vs SF-RS-M**	0.004		**Model vs SF-RS-M**	<0.001
	**Model vs SF-RS-L**	0.390		**Model vs SF-RS-L**	0.010
	**RS-H vs SF-RS-H**	0.128		**RS-H vs SF-RS-H**	0.448
	**RS-M vs SF-RS-M**	0.340		**RS-M vs SF-RS-M**	0.565
	**RS-L vs SF-RS-L**	0.614		**RS-L vs SF-RS-L**	0.849
**ALP**	**Control vs Model**	<0.001	**Oil red O**	**Control vs Model**	<0.001
	**Model vs Metformin**	<0.001		**Model vs Metformin**	<0.001
	**Model vs RS-H**	<0.001		**Model vs RS-H**	<0.001
	**Model vs RS-M**	<0.001		**Model vs RS-M**	<0.001
	**Model vs RS-L**	0.030		**Model vs RS-L**	0.524
	**Model vs SF-RS-H**	<0.001		**Model vs SF-RS-H**	<0.001
	**Model vs SF-RS-M**	<0.001		**Model vs SF-RS-M**	<0.001
	**Model vs SF-RS-L**	<0.001		**Model vs SF-RS-L**	0.003
	**RS-H vs SF-RS-H**	0.236		**RS-H vs SF-RS-H**	0.101
	**RS-M vs SF-RS-M**	0.107		**RS-M vs SF-RS-M**	0.131
	**RS-L vs SF-RS-L**	0.004		**RS-L vs SF-RS-L**	0.018
**PAS**	**Control vs Model**	<0.001	**Masson**	**Control vs Model**	<0.001
	**Model vs Metformin**	<0.001		**Model vs Metformin**	<0.001
	**Model vs RS-H**	<0.001		**Model vs RS-H**	<0.001
	**Model vs RS-M**	<0.001		**Model vs RS-M**	<0.001
	**Model vs RS-L**	<0.001		**Model vs RS-L**	<0.001
	**Model vs SF-RS-H**	<0.001		**Model vs SF-RS-H**	<0.001
	**Model vs SF-RS-M**	<0.001		**Model vs SF-RS-M**	<0.001
	**Model vs SF-RS-L**	<0.001		**Model vs SF-RS-L**	<0.001
	**RS-H vs SF-RS-H**	<0.001		**RS-H vs SF-RS-H**	0.004
	**RS-M vs SF-RS-M**	<0.001		**RS-M vs SF-RS-M**	0.007
	**RS-L vs SF-RS-L**	<0.001		**RS-L vs SF-RS-L**	<0.001

Notes: For the prespecified dose-matched RS-versus-SF-RS comparisons (RS-L vs SF-RS-L, RS-M vs SF-RS-M, and RS-H vs SF-RS-H), P values were adjusted using the Holm step-down procedure to control the family-wise error rate. P values for other post hoc group comparisons were adjusted using Dunnett's procedure. Adjusted p values < 0.05 were considered statistically

significant.

Table S15. Serum renal function-related biomarkers and multiplicity-adjusted P values

Group	Cr (μmol/L)		BUN (mmol/L)	CysC (mg/L)	
Control	40.87±1.01		5.95±0.35	4.29±0.23	
Model	100.34±15.99**		9.90±1.47**	4.23±0.43	
Metformin	60.22±14.93##		6.10±0.94##	4.20±0.36	
RS-H	63.21±7.74##		6.27±1.02##	4.31±0.21	
RS-M	67.84±9.11##		7.35±1.41##	4.15±0.60	
RS-L	68.38±3.45##		7.64±0.67##	4.14±0.31	
SF-RS-H	49.69±10.67## △		5.28±0.69##	4.20±0.48	
SF-RS-M	56.11±7.19##		6.61±0.90##	4.45±0.54	
SF-RS-L	62.75±10.64##		7.17±1.47##	4.12±0.23	
**Indicator**	**Comparison**	**Adjusted P value**	**Indicator**	**Comparison**	**Adjusted P value**
Cr	Control vs Model	<0.001	BUN	Control vs Model	<0.001
	Model vs Metformin	<0.001		Model vs Metformin	<0.001
	Model vs RS-H	<0.001		Model vs RS-H	<0.001
	Model vs RS-M	<0.001		Model vs RS-M	0.001
	Model vs RS-L	<0.001		Model vs RS-L	0.002
	Model vs SF-RS-H	<0.001		Model vs SF-RS-H	<0.001
	Model vs SF-RS-M	<0.001		Model vs SF-RS-M	<0.001
	Model vs SF-RS-L	<0.001		Model vs SF-RS-L	<0.001
	RS-H vs SF-RS-H	0.029		RS-H vs SF-RS-H	0.145
	RS-M vs SF-RS-M	0.054		RS-M vs SF-RS-M	0.701
	RS-L vs SF-RS-L	0.293		RS-L vs SF-RS-L	0.418
CysC	Control vs Model	0.801			
	Model vs Metformin	0.899			
	Model vs RS-H	0.776			
	Model vs RS-M	0.758			
	Model vs RS-L	0.723			

	**Model vs SF-RS-H**	0.887			
	**Model vs SF-RS-M**	0.401			
	**Model vs SF-RS-L**	0.647			
	**RS-H vs SF-RS-H**	0.670			
	**RS-M vs SF-RS-M**	0.253			
	**RS-L vs SF-RS-L**	0.918			

Notes: $^{**}P<0.01$ compared with control group; $^{\#\#}P<0.01$ compared with model group; the triangle indicates the significant difference in the following comparisons: RS-H versus SF-RS-H, RS-M versus SF-RS-M, RS-L versus SF-RS-L. **Notes**: For the prespecified dose-matched RS-versus-SF-RS comparisons (RS-L vs SF-RS-L, RS-M vs SF-RS-M, and RS-H vs SF-RS-H), P values were adjusted using the Holm step-down procedure. P values for other post hoc group comparisons were adjusted using Dunnett's procedure. Adjusted P values < 0.05 were considered statistically significant.

Table S16. Quality assessment of LC-MS/MS metabolomic data based on pooled QC samples

**Ion mode**	**No.**	**Peak area**		**Retention time**	
		**Mean ± SD**	**RSD (%)**	**Mean ± SD**	**RSD (%)**
**Positive**	**1**	141949557.3±8635428.7	6.08	1.06±0.014	1.28
	**2**	44969718±710064.1	1.58	2.44±0.039	1.60
	**3**	32848252±1366360.5	4.16	5.08±0.012	0.24
	**4**	149538970±3352964.9	2.24	7.45±0.0094	0.13
	**5**	103443094±2713697.2	2.62	8.87±0.020	0.22
	**6**	68930684±3218007.5	4.67	9.23±0.023	0.25
**Negative**	**7**	50384724±1786879	3.55	1.10±0.0023	0.21
	**8**	51496073±1698691	3.30	4.68±0.0071	0.15
	**9**	81003980±2657064	3.28	5.09±0.0072	0.14
	**10**	64403435±1086814	1.69	7.41±0.0062	0.083
	**11**	43431913±1507636	3.47	8.72±0.0074	0.085
	**12**	41170755±925923.1	2.25	10.53±0.0067	0.064

Table S17. Validation parameters of OPLS-DA models for discrimination among experimental groups

Parameters	OPLS-DA			Permutation tests	
	R^2X	R^2Y	Q^2	R^2	Q^2
**Control vs Model POS**	0.487	0.998	0.984	0.86	-0.28
**Control vs Model NEG**	0.581	1	0.985	0.977	-0.204
**Model vs RS POS**	0.372	0.989	0.432	0.98	-0.0157
**Model vs RS NEG**	0.331	0.994	0.235	0.994	-0.0177
**Model vs SF-RS POS**	0.484	0.996	0.912	0.962	-0.179
**Model vs SF-RS NEG**	0.369	0.995	0.775	0.982	-0.238

Table S18. Differential metabolites identified by LC-MS/MS metabolomic profiling among diabetic model, RS-treated and SF-RS-treated groups

HMDB ID	Formula	Metabolite	Adjusted *P*	*m/z*	Trend 1	Trend 2	Trend 3
HMDB0000151	$C_{18}H_{24}O_2$	Estradiol	0.02072	307.1472	↑	—	↓
HMDB0000429	$C_{18}H_{24}O_2$	17a-Estradiol	0.02072	307.1472	↑	—	↓
HMDB0002005	$C_5H_{11}NO_3S$	Methionine sulfoxide	0.02866	329.0838	↓	↑	↑
HMDB0003585	$C_3H_7NO_2S_2$	Thiocysteine	3.92E-11	364.9960	↑	—	↓
HMDB0000939	$C_{14}H_{20}N_6O_5S$	S-Adenosylhomocysteine	8.97E-10	767.2395	↑	—	↓
HMDB0002127	$C_3H_6O_3S$	3-Mercaptolactic acid	0.0002796	303.0204	↑	—	↓
HMDB0000118	$C_9H_{10}O_4$	Homovanillic acid	0.01011	217.0271	↑	—	↓
HMDB0000755	$C_9H_{10}O_4$	Hydroxyphenyllactic acid	0.01011	217.0271	↑	—	↓
HMDB0000292	$C_5H_4N_4O_2$	Xanthine	0.0001305	133.0159	↑	↑	↓
HMDB0001342	$C_{10}H_{17}N_2O_{14}P_3$	Thymidine 5'-triphosphate	2.08E-06	462.9722	↓	↑	—
HMDB0001406	$C_6H_6N_2O$	Niacinamide	9.12E-09	303.1110	↓	↑	↑
HMDB0001132	$C_{11}H_{16}NO_9P$	Nicotinic acid mononucleotide	0.003289	318.0385	↑	↓	↓
HMDB0000684	$C_{10}H_{12}N_2O_3$	L-Kynurenine	7.08E-06	189.0679	↓	↑	—
HMDB0008858	$C_{35}H_{66}NO_8P$	PE(14:1(9Z)/16:1(9Z))	3.84E-05	694.4219	↑	—	↓
HMDB0008954	$C_{35}H_{66}NO_8P$	PE(16:1(9Z)/14:1(9Z))	3.84E-05	694.4219	↑	—	↓
HMDB0000244	$C_{17}H_{20}N_4O_6$	Riboflavin	6.73E-06	797.2748	↑	↓	—
HMDB0008855	$C_{33}H_{62}NO_8P$	PE(14:1(9Z)/14:1(9Z))	1.84E-09	666.3904	↑	↓	↓
HMDB0004148	$C_8H_{11}NO_5S$	Dopamine 4-sulfate	5.46E-05	525.0842	↑	↓	↓
HMDB0006275	$C_8H_{11}NO_5S$	Dopamine 3-O-sulfate	5.46E-05	525.0842	↑	↓	↓
HMDB0006049	$C_9H_{12}NO_6P$	O-Phosphotyrosine	3.62E-05	521.0725	↑	↓	↓
HMDB0009147	$C_{39}H_{72}NO_7P$	PE(18:3(6Z,9Z,12Z)/P-16:0)	1.07E-05	678.4845	↑	↓	↓
HMDB0009180	$C_{39}H_{72}NO_7P$	PE(18:3(9Z,12Z,15Z)/P-16:0)	1.07E-05	678.4845	↑	↓	↓
HMDB0006766	$C_{24}H_{32}O_9$	Estriol-16-Glucuronide	4.58E-06	499.1717	↑	↓	—
HMDB0010333	$C_{24}H_{32}O_9$	Estriol-17-glucuronide	4.58E-06	499.1717	↑	↓	—
HMDB0000698	$C_{26}H_{43}NO_4$	Lithocholic acid glycine conjugate	1.20E-08	414.3010	↑	↓	—

HMDB0009861	$C_{49}H_{83}O_{13}P$	PI(18:3(6Z,9Z,12Z)/22:3(10Z,13Z,16Z))	0.0003394	891.5425	↓	↑	↑
HMDB0009865	$C_{49}H_{83}O_{13}P$	PI(18:3(9Z,12Z,15Z)/22:3(10Z,13Z,16Z))	0.0003394	891.5425	↓	↑	↑
HMDB0009884	$C_{49}H_{83}O_{13}P$	PI(20:3(5Z,8Z,11Z)/20:3(5Z,8Z,11Z))	0.0003394	891.5425	↓	↑	↑
HMDB0009885	$C_{49}H_{83}O_{13}P$	PI(20:3(5Z,8Z,11Z)/20:3(8Z,11Z,14Z))	0.0003394	891.5425	↓	↑	↑
HMDB0009891	$C_{49}H_{83}O_{13}P$	PI(20:3(8Z,11Z,14Z)/20:3(5Z,8Z,11Z))	0.0003394	891.5425	↓	↑	↑
HMDB0009892	$C_{49}H_{83}O_{13}P$	PI(20:3(8Z,11Z,14Z)/20:3(8Z,11Z,14Z))	0.0003394	891.5425	↓	↑	↑
HMDB0009910	$C_{49}H_{83}O_{13}P$	PI(22:3(10Z,13Z,16Z)/18:3(6Z,9Z,12Z))	0.0003394	891.5425	↓	↑	↑
HMDB0009911	$C_{49}H_{83}O_{13}P$	PI(22:3(10Z,13Z,16Z)/18:3(9Z,12Z,15Z))	0.0003394	891.5425	↓	↑	↑
HMDB0009920	$C_{49}H_{83}O_{13}P$	PI(22:6(4Z,7Z,10Z,13Z,16Z,19Z)/18:0)	0.0003394	891.5425	↓	↑	↑
HMDB0009116	$C_{41}H_{76}NO_7P$	PE(18:2(9Z,12Z)/P-18:1(11Z))	0.04124	706.5191	↑	↓	↓
HMDB0009117	$C_{41}H_{76}NO_7P$	PE(18:2(9Z,12Z)/P-18:1(9Z))	0.04124	706.5191	↑	↓	↓
HMDB0009148	$C_{41}H_{76}NO_7P$	PE(18:3(6Z,9Z,12Z)/P-18:0)	0.04124	706.5191	↑	↓	↓
HMDB0009181	$C_{41}H_{76}NO_7P$	PE(18:3(9Z,12Z,15Z)/P-18:0)	0.04124	706.5191	↑	↓	↓
HMDB0009345	$C_{41}H_{76}NO_7P$	PE(20:3(5Z,8Z,11Z)/P-16:0)	0.04124	706.5191	↑	↓	↓
HMDB0009378	$C_{41}H_{76}NO_7P$	PE(20:3(8Z,11Z,14Z)/P-16:0)	0.04124	706.5191	↑	↓	↓
HMDB0000050	$C_{10}H_{13}N_5O_4$	Adenosine	0.02621	593.2079	↑	↓	↓
HMDB0000085	$C_{10}H_{13}N_5O_4$	Deoxyguanosine	0.02621	593.2079	↑	↓	↓
HMDB0001563	$C_{11}H_{15}N_5O_5$	1-Methylguanosine	0.02621	593.2079	↑	↓	↓
HMDB0005972	$C_{21}H_{34}O_4$	Tetrahydrodeoxycortisol	7.96E-11	385.2164	↑	↓	↓
HMDB0007852	$C_{21}H_{39}O_7P$	LysoPA(0:0/18:2(9Z,12Z))	2.04E-10	415.2268	↑	↓	↓
HMDB0007856	$C_{21}H_{39}O_7P$	LysoPA(18:2(9Z,12Z)/0:0)	2.04E-10	415.2268	↑	↓	↓
HMDB0001308	$C_8H_{15}N_2O_9P$	5'-Phosphoribosyl-N-formylglycinamide	0.0014656	359.0509	↑	↓	↓
HMDB0000034	$C_5H_5N_5$	Adenine	0.02796	180.0527	↓	↑	↑
HMDB0003369	$C_{15}H_{25}N_3O_{16}P_2$	CDP-glucose	0.005857	610.0668	↓	↑	—
HMDB0000235	$C_{12}H_{17}N_4OS+$	Thiamine	3.96E-10	246.0955	↑	↓	↓
HMDB0001319	$C_8H_{12}NO_6P$	Pyridoxine 5'-phosphate	0.003241	497.0712	↑	↓	↓
HMDB0008457	$C_{46}H_{82}NO_7P$	PC(20:4(5Z,8Z,11Z,14Z)/P-18:1(11Z))	0.02591	836.5779	↓	↑	—
HMDB0008458	$C_{46}H_{82}NO_7P$	PC(20:4(5Z,8Z,11Z,14Z)/P-18:1(9Z))	0.02591	836.5779	↓	↑	—
HMDB0008490	$C_{46}H_{82}NO_7P$	PC(20:4(8Z,11Z,14Z,17Z)/P-18:1(11Z))	0.02591	836.5779	↓	↑	—
HMDB0008491	$C_{46}H_{82}NO_7P$	PC(20:4(8Z,11Z,14Z,17Z)/P-	0.02591	836.5779	↓	↑	—

		18:1(9Z))					
HMDB0008522	$C_{46}H_{82}NO_7P$	PC(20:5(5Z,8Z,11Z,14Z,17Z)/P-18:0)	0.02591	836.5779	↓	↑	—
HMDB0008685	$C_{46}H_{82}NO_7P$	PC(22:5(4Z,7Z,10Z,13Z,16Z)/P-16:0)	0.02591	836.5779	↓	↑	—
HMDB0008718	$C_{46}H_{82}NO_7P$	PC(22:5(7Z,10Z,13Z,16Z,19Z)/P-16:0)	0.02591	836.5779	↓	↑	—
HMDB0000122	$C_6H_{12}O_6$	D-Glucose	0.001348	359.1208	↑	↓	—
HMDB0000169	$C_6H_{12}O_6$	D-Mannose	0.001348	359.1208	↑	↓	—
HMDB0000211	$C_6H_{12}O_6$	myo-Inositol	0.001348	359.1208	↑	↓	—
HMDB0000660	$C_6H_{12}O_6$	D-Fructose	0.001348	359.1208	↑	↓	—
HMDB0003345	$C_6H_{12}O_6$	Alpha-D-Glucose	0.001348	359.1208	↑	↓	—
HMDB0006088	$C_6H_{12}O_6$	scyllo-Inositol	0.001348	359.1208	↑	↓	—
HMDB0003426	$C_{11}H_{22}N_2O_4S$	Pantetheine	3.87E-07	615.2724	↑	↓	↓
HMDB0006555	$C_{10}H_{13}N_4O_7P$	dIMP	1.55E-09	313.0351	↓	↑	—
HMDB0000062	$C_7H_{15}NO_3$	L-Carnitine	0.01862	367.2087	↑	↓	—
HMDB0006485	$C_{20}H_{21}N_7O_7$	10-Formyldihydrofolate	0.03492	506.1196	↑	—	↓
HMDB0002013	$C_{11}H_{21}NO_4$	Butyrylcarnitine	0.003371	461.2864	↑	↓	↓
HMDB0002666	$C_{12}H_{17}N_4O_4PS$	Thiamine monophosphate	0.01877	379.0395	↓	↑	↑
HMDB0000030	$C_{10}H_{16}N_2O_3S$	Biotin	2.50E-07	547.1923	↓	↑	↑
HMDB0001514	$C_6H_{13}NO_5$	Glucosamine	0.01695	214.0482	↓	↑	↑
HMDB0000012	$C_9H_{12}N_2O_5$	Deoxyuridine	0.03480	227.0684	↓	↑	↑
HMDB0000563	$C_9H_{10}O_3$	D-Phenyllactic acid	0.0009992	331.1180	↓	—	↑
HMDB0000748	$C_9H_{10}O_3$	L-3-Phenyllactic acid	0.0009992	331.1180	↓	—	↑
HMDB0000779	$C_9H_{10}O_3$	Phenyllactic acid	0.0009992	331.1180	↓	—	↑
HMDB0002199	$C_9H_{10}O_3$	Desaminotyrosine	0.0009992	331.1180	↓	—	↑
HMDB0001178	$C_{15}H_{23}N_5O_{14}P_2$	Adenosine diphosphate ribose	0.03020	540.0533	↓	↑	↑
HMDB0004827	$C_7H_{13}NO_2$	Proline betaine	6.36E-08	331.1878	↑	↓	↓
HMDB0000895	$C_7H_{16}NO_2+$	Acetylcholine	2.51E-09	337.2349	↑	↓	↓
HMDB0010318	$C_{27}H_{44}O_8$	Pregnanediol-3-glucuronide	3.57E-06	541.3005	↓	↑	↓
HMDB0009611	$C_{45}H_{80}NO_7P$	PE(22:4(7Z,10Z,13Z,16Z)/P-18:1(11Z))	0.001134	812.5370	↑	↓	—
HMDB0009612	$C_{45}H_{80}NO_7P$	PE(22:4(7Z,10Z,13Z,16Z)/P-18:1(9Z))	0.001134	812.5370	↑	↓	—
HMDB0009643	$C_{45}H_{80}NO_7P$	PE(22:5(4Z,7Z,10Z,13Z,16Z)/P-18:0)	0.001134	812.5370	↑	↓	—
HMDB0009676	$C_{45}H_{80}NO_7P$	PE(22:5(7Z,10Z,13Z,16Z,19Z)/P-18:0)	0.001134	812.5370	↑	↓	—
HMDB0002710	$C_{20}H_{30}O_4$	Prostaglandin J2	0.006828	379.2123	↓	↑	↓
HMDB0002752	$C_{20}H_{30}O_4$	Prostaglandin A2	0.006828	379.2123	↓	↑	↓
HMDB0004095	$C_{11}H_{14}N_2O$	5-Methoxytryptamine	0.006828	379.2123	↓	↑	↓
HMDB0004234	$C_{20}H_{30}O_4$	12-Keto-leukotriene B4	0.006828	379.2123	↓	↑	↓
HMDB0004236	$C_{20}H_{30}O_4$	Prostaglandin B2	0.006828	379.2123	↓	↑	↓

HMDB0004238	$C_{20}H_{30}O_4$	Delta-12-Prostaglandin J2	0.006828	379.2123	↓	↑	↓
HMDB0004369	$C_{11}H_{14}N_2O$	N-Methylserotonin	0.006828	379.2123	↓	↑	↓
HMDB0010391	$C_{28}H_{56}NO_7P$	LysoPC(20:1(11Z))	4.29E-09	584.3499	↑	↓	—
HMDB0011148	$C_{28}H_{56}NO_7P$	PC(18:1(9Z)e/2:0)	4.29E-09	584.3499	↑	↓	—
HMDB0004231	$C_9H_{19}NO_4$	Pantothenol	4.22E-15	455.2613	↑	↓	↓
HMDB0009511	$C_{45}H_{90}NO_7P$	PE(22:0/P-18:0)	3.81E-05	822.6153	↑	↓	↓
HMDB0009741	$C_{45}H_{90}NO_7P$	PE(24:0/P-16:0)	3.81E-05	822.6153	↑	↓	↓
HMDB0008822	$C_{33}H_{64}NO_8P$	PE(14:0/14:1(9Z))	6.50E-10	668.4056	↑	↓	↓
HMDB0008854	$C_{33}H_{64}NO_8P$	PE(14:1(9Z)/14:0)	6.50E-10	668.4056	↑	↓	↓
HMDB0001328	$C_{16}H_{26}N_2O_{16}P_2$	dTDP-D-glucose	0.005601	603.0412	↓	↑	—
HMDB0006876	$C_{16}H_{26}N_2O_{16}P_2$	dTDP-D-galactose	0.005601	603.0412	↓	↑	—
HMDB0001368	$C_3H_4O_3S$	3-Mercaptopyruvic acid	1.89E-14	240.9835	↑	↓	—
HMDB0001030	$C_{12}H_{20}O_{11}$	3'-Ketolactose	0.03381	305.0872	↑	↓	↓
HMDB0000401	$C_5H_5N_5O_2$	2,8-Dihydroxyadenine	0.04285	190.0340	↑	↓	—
HMDB0002032	$C_5H_5N_5O_2$	8-Hydroxyguanine	0.002565	190.0340	↑	↓	↓
HMDB0000197	$C_{10}H_9NO_2$	Indoleacetic acid	0.0007881	351.1353	↑	↓	↓
HMDB0004073	$C_{10}H_9NO_2$	5-Hydroxyindoleacetaldehyde	0.002501	351.1353	↓	↑	—
HMDB0001202	$C_9H_{14}N_3O_7P$	dCMP	5.46E-10	272.0427	↓	↑	—
HMDB0002070	$C_5H_6O_6$	4-Hydroxy-2-oxoglutaric acid	0.01228	185.0060	↓	↓	↑
HMDB0000037	$C_{21}H_{28}O_5$	Aldosterone	1.39E-08	378.2264	↑	↓	—
HMDB0002802	$C_{21}H_{28}O_5$	Cortisone	1.39E-08	378.2264	↑	↓	—
HMDB0008189	$C_{48}H_{78}NO_8P$	PC(18:3(6Z,9Z,12Z)/22:6(4Z,7Z,10Z,13Z,16Z,19Z))	5.40E-09	866.5136	↓	↑	↓
HMDB0008222	$C_{48}H_{78}NO_8P$	PC(18:3(9Z,12Z,15Z)/22:6(4Z,7Z,10Z,13Z,16Z,19Z))	5.40E-09	866.5136	↓	↑	↓
HMDB0008253	$C_{48}H_{78}NO_8P$	PC(18:4(6Z,9Z,12Z,15Z)/22:5(4Z,7Z,10Z,13Z,16Z))	5.40E-09	866.5136	↓	↑	↓
HMDB0008254	$C_{48}H_{78}NO_8P$	PC(18:4(6Z,9Z,12Z,15Z)/22:5(7Z,10Z,13Z,16Z,19Z))	5.40E-09	866.5136	↓	↑	↓
HMDB0008445	$C_{48}H_{78}NO_8P$	PC(20:4(5Z,8Z,11Z,14Z)/20:5(5Z,8Z,11Z,14Z,17Z))	5.40E-09	866.5136	↓	↑	↓
HMDB0008478	$C_{48}H_{78}NO_8P$	PC(20:4(8Z,11Z,14Z,17Z)/20:5(5Z,8Z,11Z,14Z,17Z))	5.40E-09	866.5136	↓	↑	↓
HMDB0008509	$C_{48}H_{78}NO_8P$	PC(20:5(5Z,8Z,11Z,14Z,17Z)/20:4(5Z,8Z,11Z,14Z))	5.40E-09	866.5136	↓	↑	↓
HMDB0008510	$C_{48}H_{78}NO_8P$	PC(20:5(5Z,8Z,11Z,14Z,17Z)/20:4(8Z,11Z,14Z,17Z))	5.40E-09	866.5136	↓	↑	↓
HMDB0008667	$C_{48}H_{78}NO_8P$	PC(22:5(4Z,7Z,10Z,13Z,16Z)/18:4(6Z,9Z,12Z,15Z))	5.40E-09	866.5136	↓	↑	↓
HMDB0008700	$C_{48}H_{78}NO_8P$	PC(22:5(7Z,10Z,13Z,16Z,19Z)/18:4(6Z,9Z,12Z,15Z))	5.40E-09	866.5136	↓	↑	↓
HMDB0008731	$C_{48}H_{78}NO_8P$	PC(22:6(4Z,7Z,10Z,13Z,16Z,19Z)/18:3(6Z,9Z,12Z))	5.40E-09	866.5136	↓	↑	↓

HMDB0008732	$C_{48}H_{78}NO_8P$	PC(22:6(4Z,7Z,10Z,13Z,16Z,19Z)/18:3(9Z,12Z,15Z))	5.40E-09	866.5136	↓	↑	↓
HMDB0008896	$C_{38}H_{70}NO_8P$	PE(15:0/18:3(6Z,9Z,12Z))	0.009882	738.4486	↑	↓	—
HMDB0008897	$C_{38}H_{70}NO_8P$	PE(15:0/18:3(9Z,12Z,15Z))	0.009882	738.4486	↑	↓	—
HMDB0009120	$C_{38}H_{70}NO_8P$	PE(18:3(6Z,9Z,12Z)/15:0)	0.009882	738.4486	↑	↓	—
HMDB0009153	$C_{38}H_{70}NO_8P$	PE(18:3(9Z,12Z,15Z)/15:0)	0.009882	738.4486	↑	↓	—
HMDB0007883	$C_{42}H_{76}NO_8P$	PC(14:0/20:4(5Z,8Z,11Z,14Z))	7.77E-16	792.4933	↓	↑	↑
HMDB0007884	$C_{42}H_{76}NO_8P$	PC(14:0/20:4(8Z,11Z,14Z,17Z))	7.77E-16	792.4933	↓	↑	↑
HMDB0007914	$C_{42}H_{76}NO_8P$	PC(14:1(9Z)/20:3(5Z,8Z,11Z))	7.77E-16	792.4933	↓	↑	↑
HMDB0007915	$C_{42}H_{76}NO_8P$	PC(14:1(9Z)/20:3(8Z,11Z,14Z))	7.77E-16	792.4933	↓	↑	↑
HMDB0007976	$C_{42}H_{76}NO_8P$	PC(16:0/18:4(6Z,9Z,12Z,15Z))	7.77E-16	792.4933	↓	↑	↑
HMDB0008007	$C_{42}H_{76}NO_8P$	PC(16:1(9Z)/18:3(6Z,9Z,12Z))	7.77E-16	792.4933	↓	↑	↑
HMDB0008008	$C_{42}H_{76}NO_8P$	PC(16:1(9Z)/18:3(9Z,12Z,15Z))	7.77E-16	792.4933	↓	↑	↑
HMDB0008167	$C_{42}H_{76}NO_8P$	PC(18:3(6Z,9Z,12Z)/16:1(9Z))	7.77E-16	792.4933	↓	↑	↑
HMDB0008200	$C_{42}H_{76}NO_8P$	PC(18:3(9Z,12Z,15Z)/16:1(9Z))	7.77E-16	792.4933	↓	↑	↑
HMDB0008232	$C_{42}H_{76}NO_8P$	PC(18:4(6Z,9Z,12Z,15Z)/16:0)	7.77E-16	792.4933	↓	↑	↑
HMDB0008362	$C_{42}H_{76}NO_8P$	PC(20:3(5Z,8Z,11Z)/14:1(9Z))	7.77E-16	792.4933	↓	↑	↑
HMDB0008395	$C_{42}H_{76}NO_8P$	PC(20:3(8Z,11Z,14Z)/14:1(9Z))	7.77E-16	792.4933	↓	↑	↑
HMDB0008426	$C_{42}H_{76}NO_8P$	PC(20:4(5Z,8Z,11Z,14Z)/14:0)	7.77E-16	792.4933	↓	↑	↑
HMDB0008459	$C_{42}H_{76}NO_8P$	PC(20:4(8Z,11Z,14Z,17Z)/14:0)	7.77E-16	792.4933	↓	↑	↑
HMDB0008910	$C_{42}H_{76}NO_8P$	PE(15:0/22:4(7Z,10Z,13Z,16Z))	7.77E-16	792.4933	↓	↑	↑
HMDB0000205	$C_9H_8O_3$	Phenylpyruvic acid	2.54E-07	203.0099	↓	↑	↓
HMDB0002035	$C_9H_8O_3$	4-Hydroxycinnamic acid	2.54E-07	203.0099	↓	↑	↓
HMDB0002641	$C_9H_8O_3$	2-Hydroxycinnamic acid	2.54E-07	203.0099	↓	↑	↓
HMDB0000286	$C_{15}H_{24}N_2O_{17}P_2$	Uridine diphosphate glucose	1.61E-07	549.0511	↑	↓	↓
HMDB0000302	$C_{15}H_{24}N_2O_{17}P_2$	Uridine diphosphategalactose	1.61E-07	549.0511	↑	↓	↓
HMDB0000897	$C_6H_7N_5O$	7-Methylguanine	4.44E-16	188.0550	↑	↓	↓
HMDB0001566	$C_6H_7N_5O$	3-Methylguanine	4.44E-16	188.0550	↑	↓	↓
HMDB0003282	$C_6H_7N_5O$	1-Methylguanine	4.44E-16	188.0550	↑	↓	↓
HMDB0006040	$C_6H_7N_5O$	N2-Methylguanine	4.44E-16	188.0550	↑	↓	↓
HMDB0004058	$C_8H_7NO_2$	5,6-Dihydroxyindole	0.02003	299.1023	↑	↓	↓
HMDB0000840	$C_9H_9NO_4$	Salicyluric acid	0.008404	218.0423	↑	↓	↓
HMDB0001321	$C_4H_9O_7P$	D-Erythrose 4-phosphate	0.01005	218.0423	↑	↓	↓
HMDB0000061	$C_{10}H_{15}N_5O_{10}P_2$	Adenosine 3',5'-diphosphate	5.02E-08	410.0270	↓	↑	↑
HMDB0000960	$C_{10}H_{15}N_5O_{10}P_2$	dGDP	5.02E-08	410.0270	↓	↑	↑
HMDB0001341	$C_{10}H_{15}N_5O_{10}P_2$	ADP	5.02E-08	410.0270	↓	↑	↑
HMDB0000489	$C_{12}H_{21}N_3O_8$	Aspartylglycosamine	1.74E-14	318.1299	↑	↓	—
HMDB0007956	$C_{45}H_{80}NO_8P$	PC(15:0/22:5(4Z,7Z,10Z,13Z,16Z))	7.71E-11	811.5945	↓	—	↑
HMDB0007957	$C_{45}H_{80}NO_8P$	PC(15:0/22:5(7Z,10Z,13Z,16Z,19Z))	7.71E-11	811.5945	↓	—	↑
HMDB0008658	$C_{45}H_{80}NO_8P$	PC(22:5(4Z,7Z,10Z,13Z,16Z)/15:0)	7.71E-11	811.5945	↓	—	↑

HMDB0008691	$C_{45}H_{80}NO_8P$	PC(22:5(7Z,10Z,13Z,16Z,19Z)/15:0)	7.71E-11	811.5945	↓	—	↑
HMDB0009010	$C_{45}H_{80}NO_8P$	PE(18:0/22:5(4Z,7Z,10Z,13Z,16Z))	7.71E-11	811.5945	↓	—	↑
HMDB0009011	$C_{45}H_{80}NO_8P$	PE(18:0/22:5(7Z,10Z,13Z,16Z,19Z))	7.71E-11	811.5945	↓	—	↑
HMDB0009042	$C_{45}H_{80}NO_8P$	PE(18:1(11Z)/22:4(7Z,10Z,13Z,16Z))	7.71E-11	811.5945	↓	—	↑
HMDB0009075	$C_{45}H_{80}NO_8P$	PE(18:1(9Z)/22:4(7Z,10Z,13Z,16Z))	7.71E-11	811.5945	↓	—	↑
HMDB0009140	$C_{45}H_{80}NO_8P$	PE(18:3(6Z,9Z,12Z)/22:2(13Z,16Z))	7.71E-11	811.5945	↓	—	↑
HMDB0009173	$C_{45}H_{80}NO_8P$	PE(18:3(9Z,12Z,15Z)/22:2(13Z,16Z))	7.71E-11	811.5945	↓	—	↑
HMDB0009205	$C_{45}H_{80}NO_8P$	PE(18:4(6Z,9Z,12Z,15Z)/22:1(13Z))	7.71E-11	811.5945	↓	—	↑
HMDB0009236	$C_{45}H_{80}NO_8P$	PE(20:0/20:5(5Z,8Z,11Z,14Z,17Z))	7.71E-11	811.5945	↓	—	↑
HMDB0001316	$C_6H_{13}O_{10}P$	6-Phosphogluconic acid	6.67E-10	277.0325	↑	↑	—
HMDB0000510	$C_6H_{11}NO_4$	Aminoadipic acid	4.14E-13	323.1438	↓	↑	↑
HMDB0000048	$C_{12}H_{22}O_{11}$	Melibiose	1.67E-07	360.1510	↑	↓	↓
HMDB0000055	$C_{12}H_{22}O_{11}$	Cellobiose	1.67E-07	360.1510	↑	↓	↓
HMDB0000163	$C_{12}H_{22}O_{11}$	D-Maltose	1.67E-07	360.1510	↑	↓	↓
HMDB0000186	$C_{12}H_{22}O_{11}$	Alpha-Lactose	1.67E-07	360.1510	↑	↓	↓
HMDB0000258	$C_{12}H_{22}O_{11}$	Sucrose	1.67E-07	360.1510	↑	↓	↓
HMDB0000740	$C_{12}H_{22}O_{11}$	Lactulose	1.67E-07	360.1510	↑	↓	↓
HMDB0000975	$C_{12}H_{22}O_{11}$	Trehalose	1.67E-07	360.1510	↑	↓	↓
HMDB0002923	$C_{12}H_{22}O_{11}$	Isomaltose	1.67E-07	360.1510	↑	↓	↓
HMDB0005826	$C_{12}H_{22}O_{11}$	Galactinol	1.67E-07	360.1510	↑	↓	↓
HMDB0006792	$C_{12}H_{22}O_{11}$	Epimelibiose	1.67E-07	360.1510	↑	↓	↓
HMDB0000268	$C_{21}H_{34}O_4$	Tetrahydrocorticosterone	3.35E-14	373.2344	↑	↑	—
HMDB0000101	$C_{10}H_{13}N_5O_3$	Deoxyadenosine	1.98E-07	252.1082	↑	↑	—
HMDB0001983	$C_{10}H_{13}N_5O_3$	5'-Deoxyadenosine	1.98E-07	252.1082	↓	↑	↑
HMDB0002224	$C_{10}H_{15}N_3O_4$	5-Methyldeoxycytidine	1.12E-14	259.1401	↓	↑	↑
HMDB0003235	$C_{20}H_{32}O_6$	Prostaglandin G2	4.63E-13	386.2525	↑	↓	—
HMDB0004241	$C_{20}H_{32}O_6$	6-Ketoprostaglandin E1	4.63E-13	386.2525	↑	↓	↓
HMDB0004242	$C_{20}H_{32}O_6$	11-Dehydro-thromboxane B2	4.63E-13	386.2525	↑	↓	↓
HMDB0000077	$C_{19}H_{28}O_2$	Dehydroepiandrosterone	3.30E-07	311.1983	↓	↑	↑
HMDB0000234	$C_{19}H_{28}O_2$	Testosterone	3.30E-07	311.1983	↓	↑	↑
HMDB0000628	$C_{19}H_{28}O_2$	Epitestosterone	3.30E-07	311.1983	↓	↑	↑
HMDB0000899	$C_{19}H_{28}O_2$	Androstanedione	3.30E-07	311.1983	↓	↑	↑
HMDB0001513	$C_{12}H_{26}O_7P_2$	Dolichyl diphosphate	7.03E-10	383.0788	↑	↓	—

Trend1 indicates the trend of changes in metabolites that are different in the model group compared

to the control group. **Trend 2** represents the trend of changes in metabolites that are different in the RS group compared to the model group. **Trend 3** demonstrates the trend of changes in metabolites that are different in the SF-RS group compared to the model group.

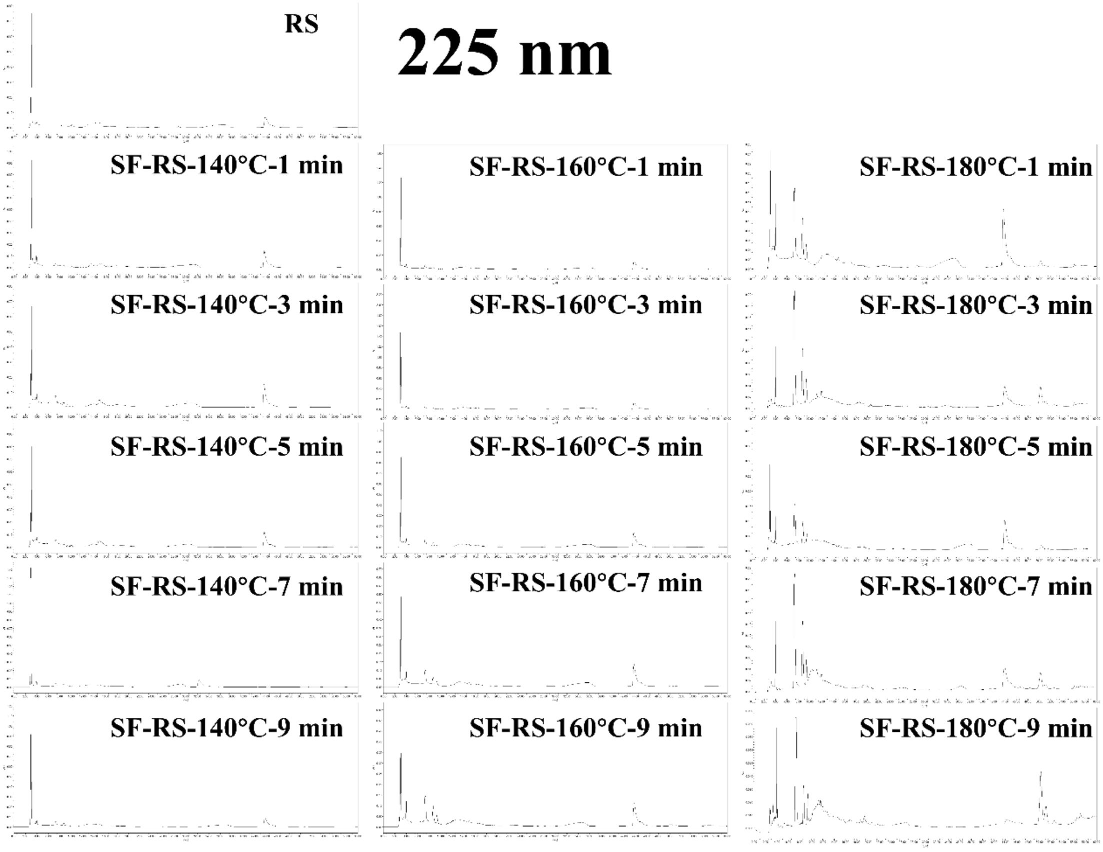

Figure S1. Representative chromatograms of the raw and stir-fried RS samples detected under the 225 nm wavelength

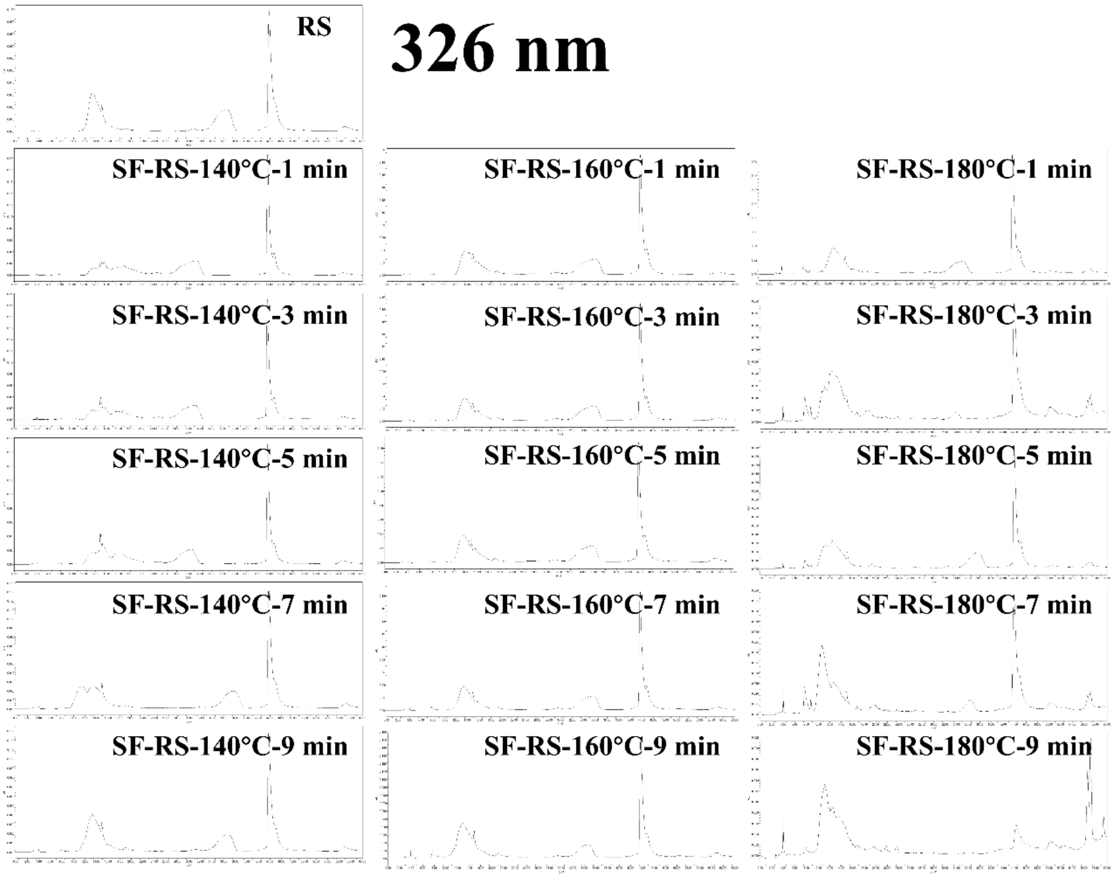

Figure S2. Representative chromatograms of the raw and stir-fried RS samples detected under the 325 nm wavelength

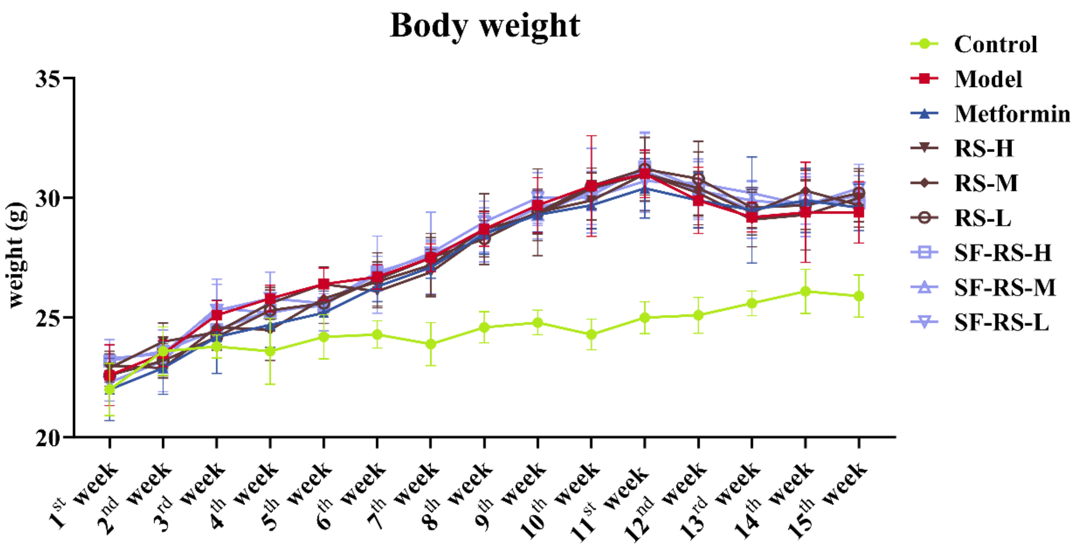

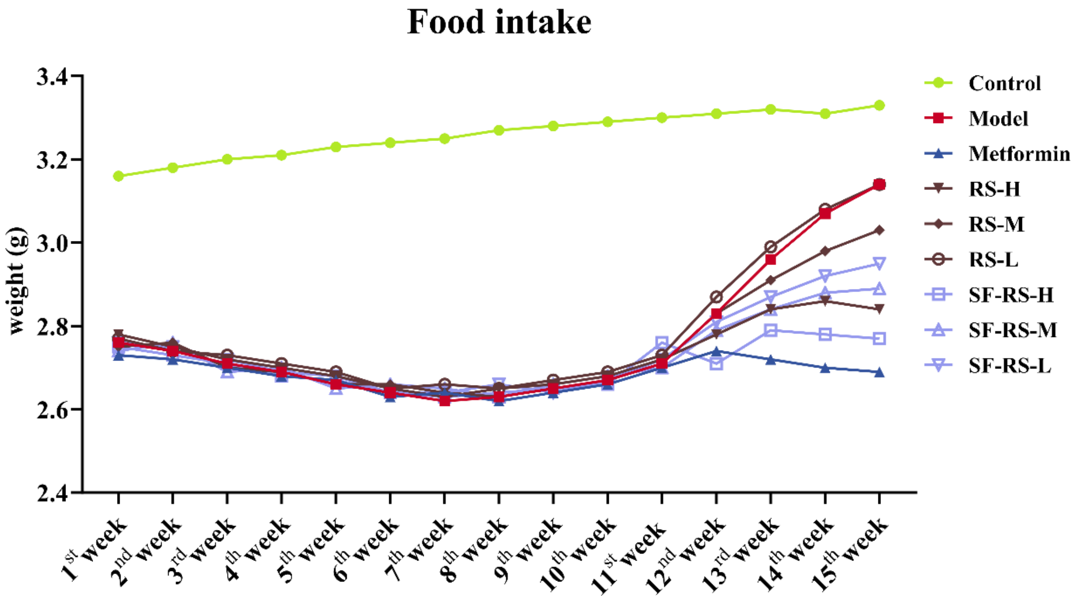

Figure S3. The visualization analysis of body weight and food intake

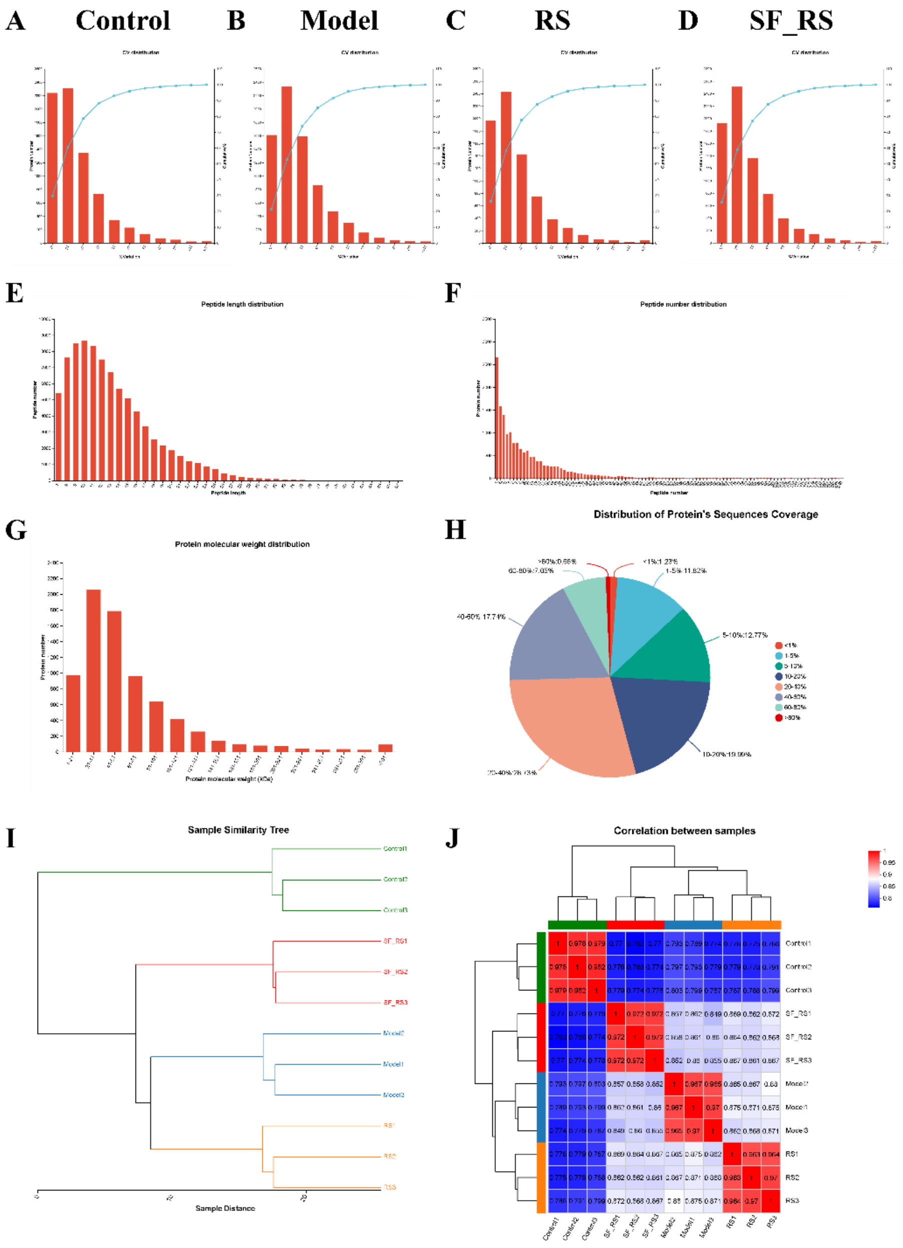

Figure S4. Quality assessment of the LC-MS/MS-based proteomic dataset. Comprehensive quality control of the proteomic data was performed prior to downstream analysis. The distribution of peptide length, peptide number per protein, protein molecular weight, and sequence coverage demonstrated efficient enzymatic digestion and broad proteome coverage. Coefficient of variation (CV) analysis indicated low variability among biological replicates. Hierarchical clustering and Pearson correlation analysis further confirmed high intra-group consistency and clear inter-group separation, supporting the robustness and reproducibility of the dataset for subsequent comparative proteomic analysis.

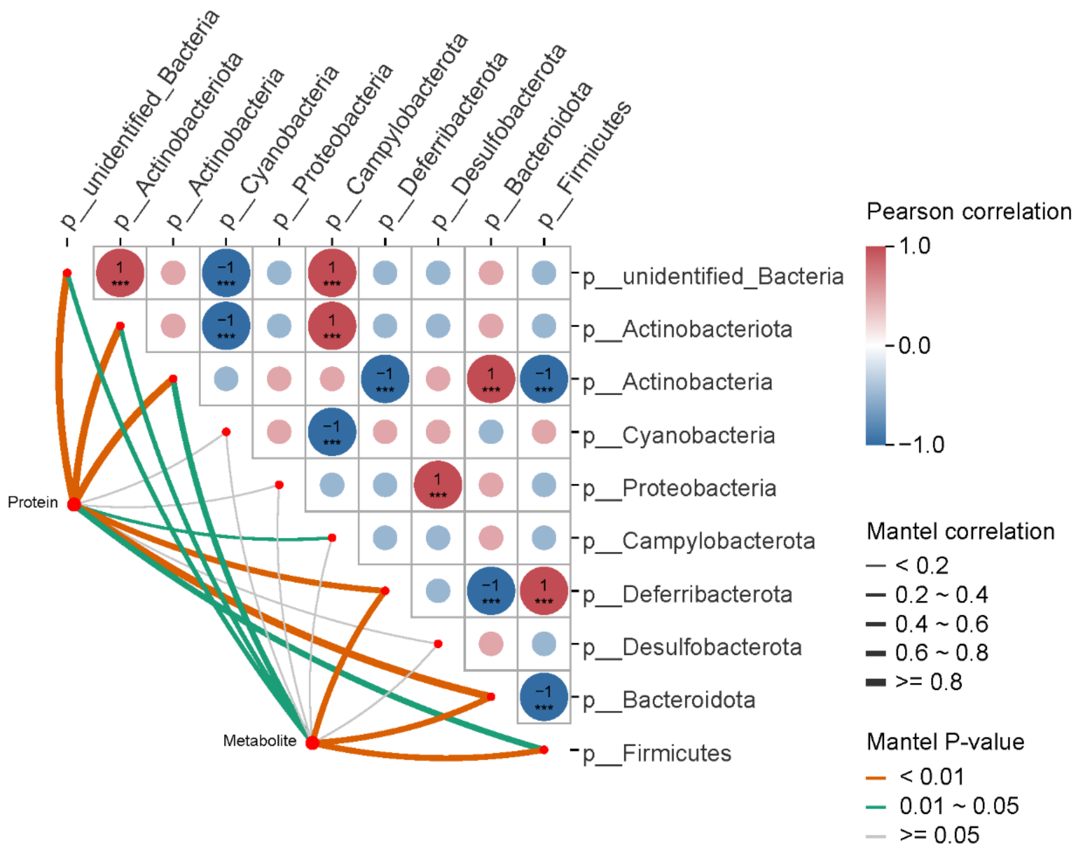

Figure S5. Mantel test-based integration of gut microbial phyla with hepatic proteomic and metabolomic profiles in T2DM mice. Pairwise Pearson correlations among the relative abundances of the dominant bacterial phyla are displayed in the triangular correlation matrix. Red and blue circles represent positive and negative correlations, respectively, whereas circle size and color intensity reflect the absolute magnitude of the Pearson correlation coefficient. Where shown, the values inside the circles indicate Pearson's *r*, and asterisks denote statistical significance. Mantel tests were performed to evaluate the correspondence between distance matrices derived from individual bacterial phylum abundances and those generated from the hepatic protein or metabolite abundance profiles. Connections from the Protein and Metabolite nodes to individual bacterial phyla represent the corresponding Mantel associations.

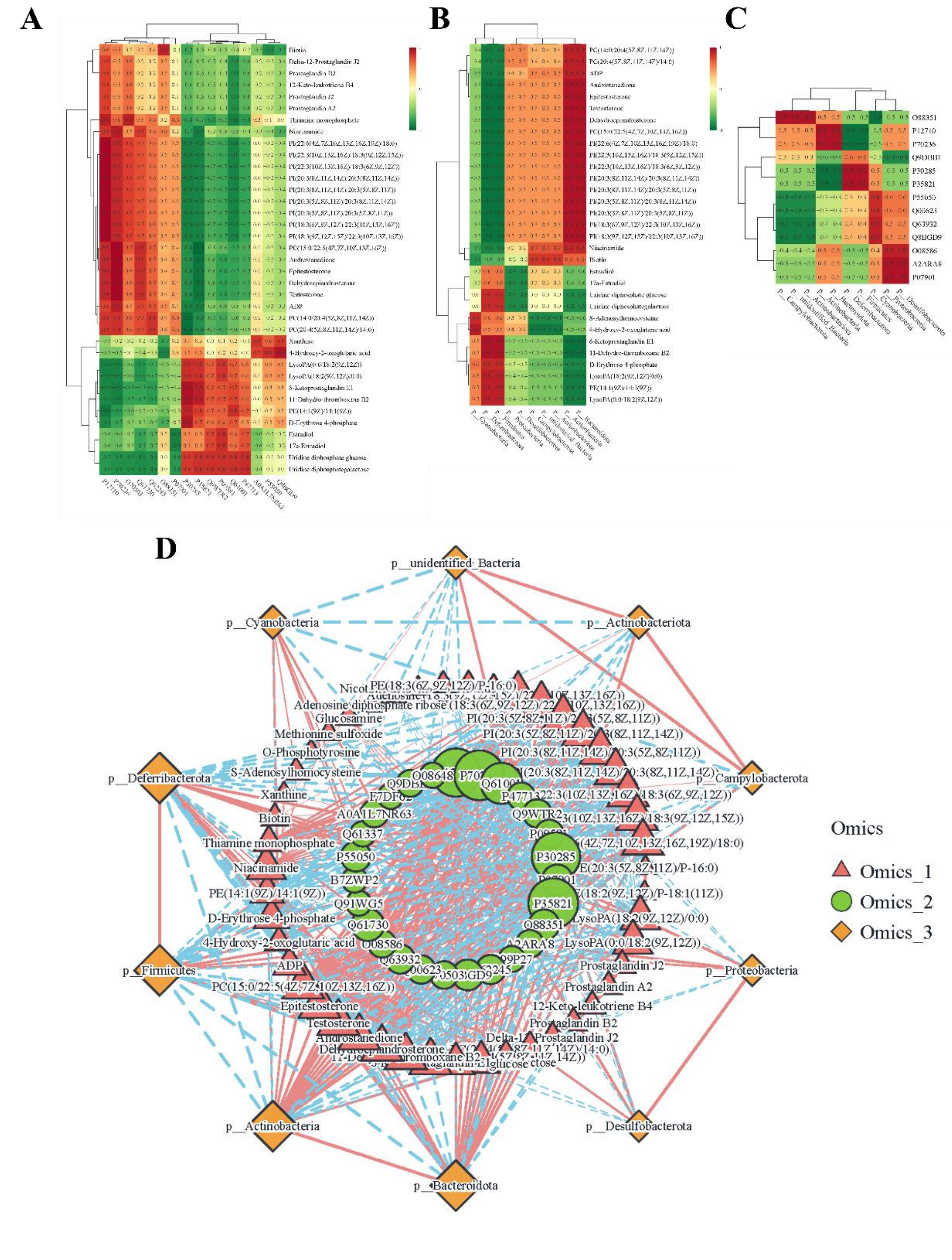

Figure S6. Integrated correlation analysis of hepatic metabolomic, hepatic proteomic, and gut microbiota profiles in T2DM mice following stir-fried *Raphani Semen* intervention. (A) Correlation analysis between hepatic metabolites and proteins. (B) Correlation analysis between hepatic metabolites and gut microbial phyla. (C) Correlation analysis between hepatic proteins and gut microbial phyla. (D) Integrated interaction network of metabolites, proteins, and gut microbiota.
